# Supplementary material for: Interrogating RNA and protein spatial subcellular distribution in smFISH data with DypFISH
Source: Cell Rep Methods. 2021 Sep 13;1(5):100068. doi: 10.1016/j.crmeth.2021.100068 (PMC9017151; doi:10.1016/j.crmeth.2021.100068)
Supplement: Document S1. Figures S1–S8 [file mmc1.pdf]

Cell Reports Methods, Volume *1*

Supplemental information

Interrogating RNA and protein spatial subcellular distribution in smFISH data with DypFISH

Anca F. Savulescu, Robyn Brackin, Emmanuel Bouilhol, Benjamin Dartigues, Jonathan H. Warrell, Mafalda R. Pimentel, Nicolas Beaume, Isabela C. Fortunato, Stephane Dallongeville, Mikaël Boulle, Hayssam Soueidan, Fabrice Agou, Jan Schmoranzer, Jean-Christophe Olivo-Marin, Claudio A. Franco, Edgar R. Gomes, Macha Nikolski, and Musa M. Mhlanga

SUPPLEMENTARY FIGURES

Figure S1, related to Figure 2

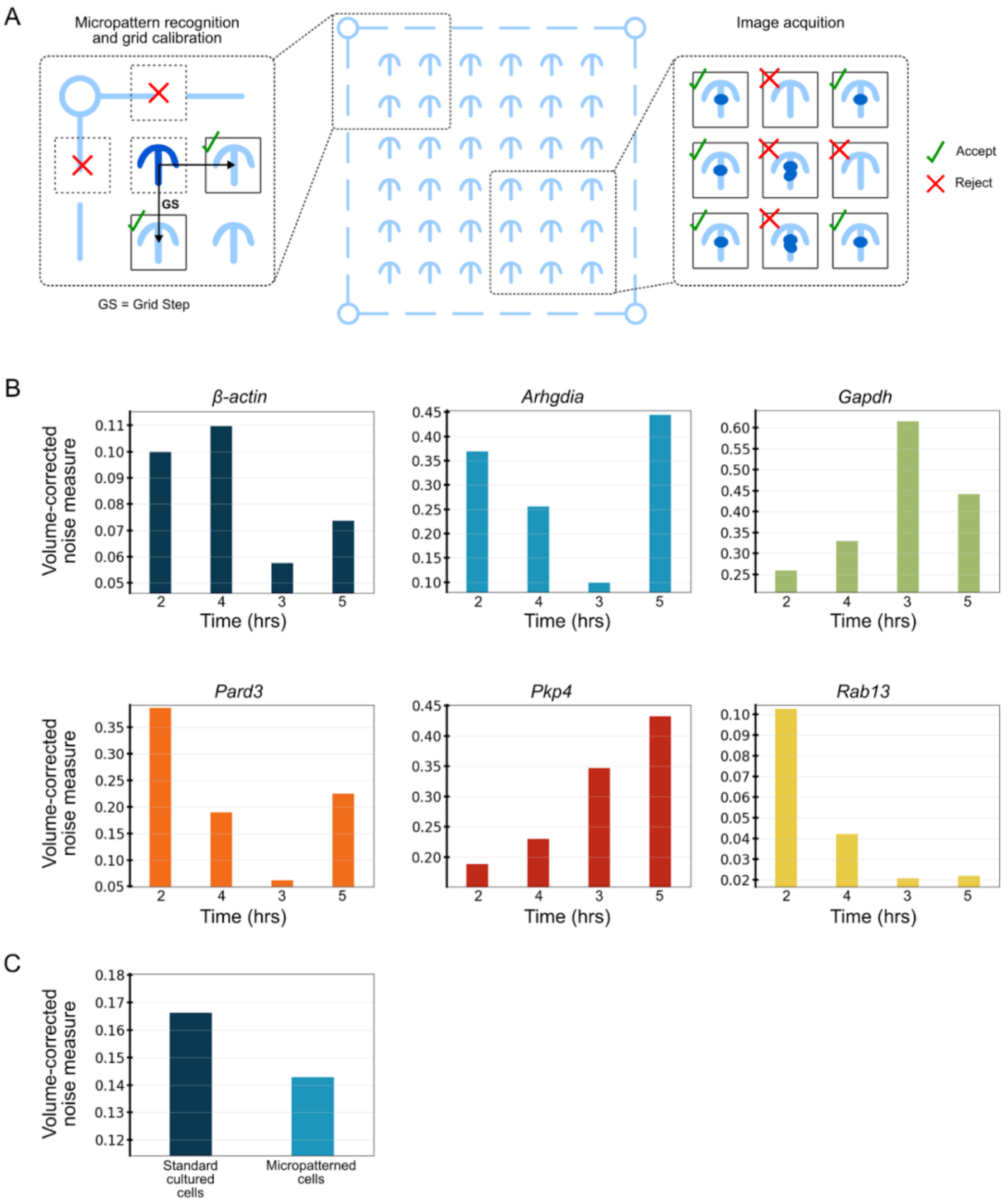

Figure S1, related to Figure 2 | Automated image acquisition and effects of micropatterning on noise. (A) Each coverslip was micro-fabricated to contain multiple 12 by 12 grids of micropatterns to which the cells adhered, facilitating the development of an algorithm to automate the process of image acquisition. The algorithm initially performed grid calibration in which the location of the upper-left micropattern was automatically detected, followed by grid orientation and grid step size determination (left). Images were then collected at each grid position across the full 12 by 12 grid. A 2-class support vector machine was trained to classify cells which have grown normally on the micropatterns versus micropatterns containing no cells, multiple cells, or cells which have failed to fill the micropattern, allowing the automatic rejection of grid positions which cannot be used (right). (B) Mouse fibroblasts were plated on fibronectin-coated micropatterns and induced to polarize by addition of serum. Cells were fixed and single molecule FISH was performed to target mRNAs of interest. The volume-corrected noise measure (Padovan-Merhar et al., 2015) was compared

across time for 6 mRNAs at 2, 3, 4 and 5h time points. (C) The stochasticity, which remains after correcting for the linear relationship between cell-size and transcript number using the volume-corrected noise measure (Padovan-Merhar et. al. 2015).

Figure S2, related to Figure 3

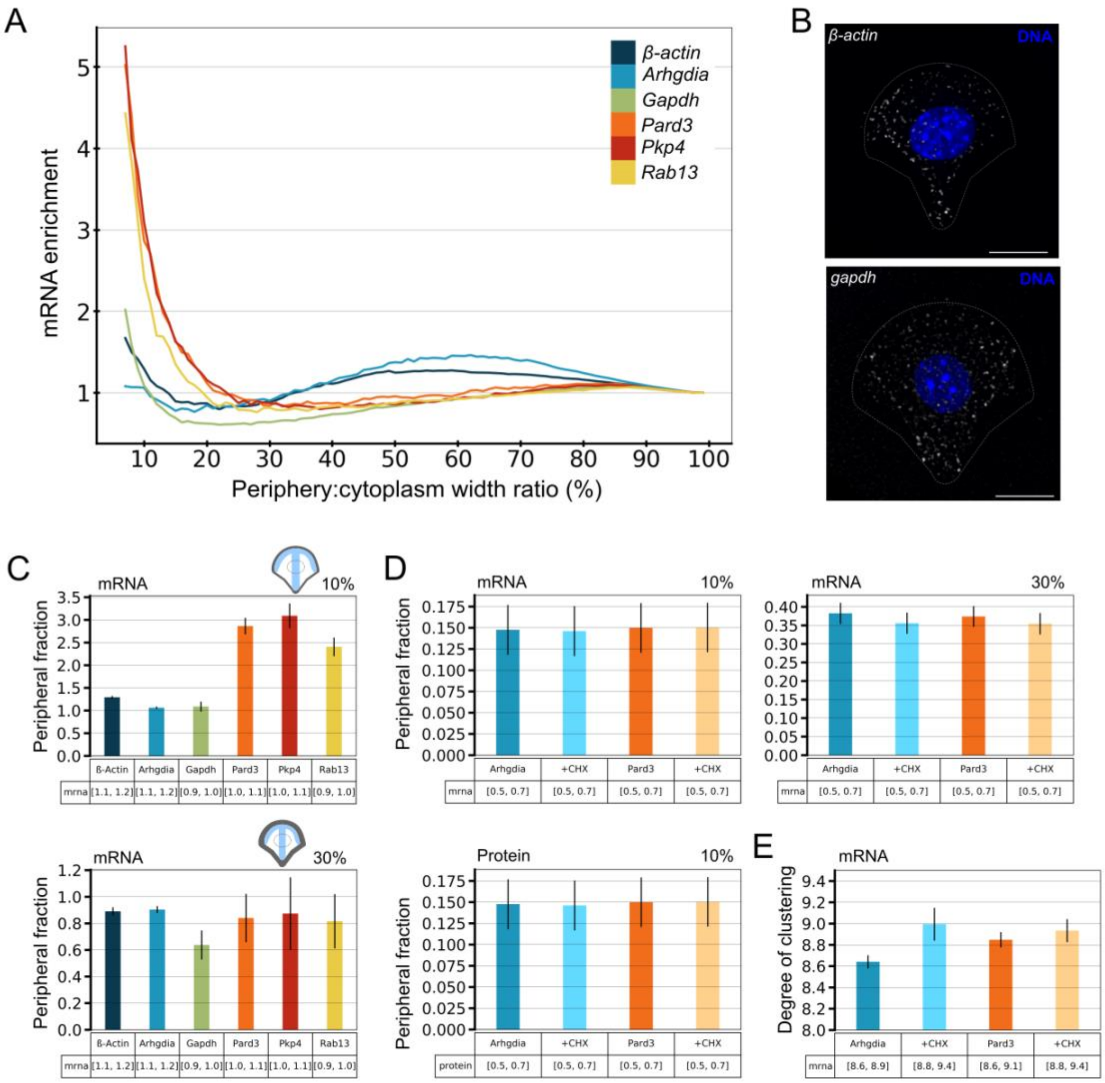

Figure S2, related to Figure 3 | Peripheral enrichment and clustering dynamics of mRNA-protein pairs. (A) Comparison of the enrichment of 5 mRNAs in a peripheral cellular region whose width varies from 0-100% of the radial distance from the plasma membrane to the nucleus. (B) Representative smFISH images for *β-Actin* and *Gapdh* transcripts. (C) Distributions of fractional values for peripheral enrichment of all mRNA transcripts at 10% and 30% of the radial distance from the plasma membrane to the nucleus. (D) Distributions of absolute fractional values for peripheral enrichment of Arhgdia and Pard3 mRNA and proteins in cells treated with CHX compared to control cells at 10% and 30% (only for mRNAs, corresponding graph for proteins in main Figure) of the radial distance from the plasma membrane to the nucleus. (E) Degree of clustering for *Arhgdia* and *Pard3* in cells treated with CHX compared to control cells. Differences in degree of clustering were assessed using a Mann-Whitney test p-value (*Arhgdia* vs *Arhgdia*+CHX = 0.092; Pard3 vs Pard3+CHX = 0.71). Error bars show the standard error of the median.

Figure S3, related to Figure 4

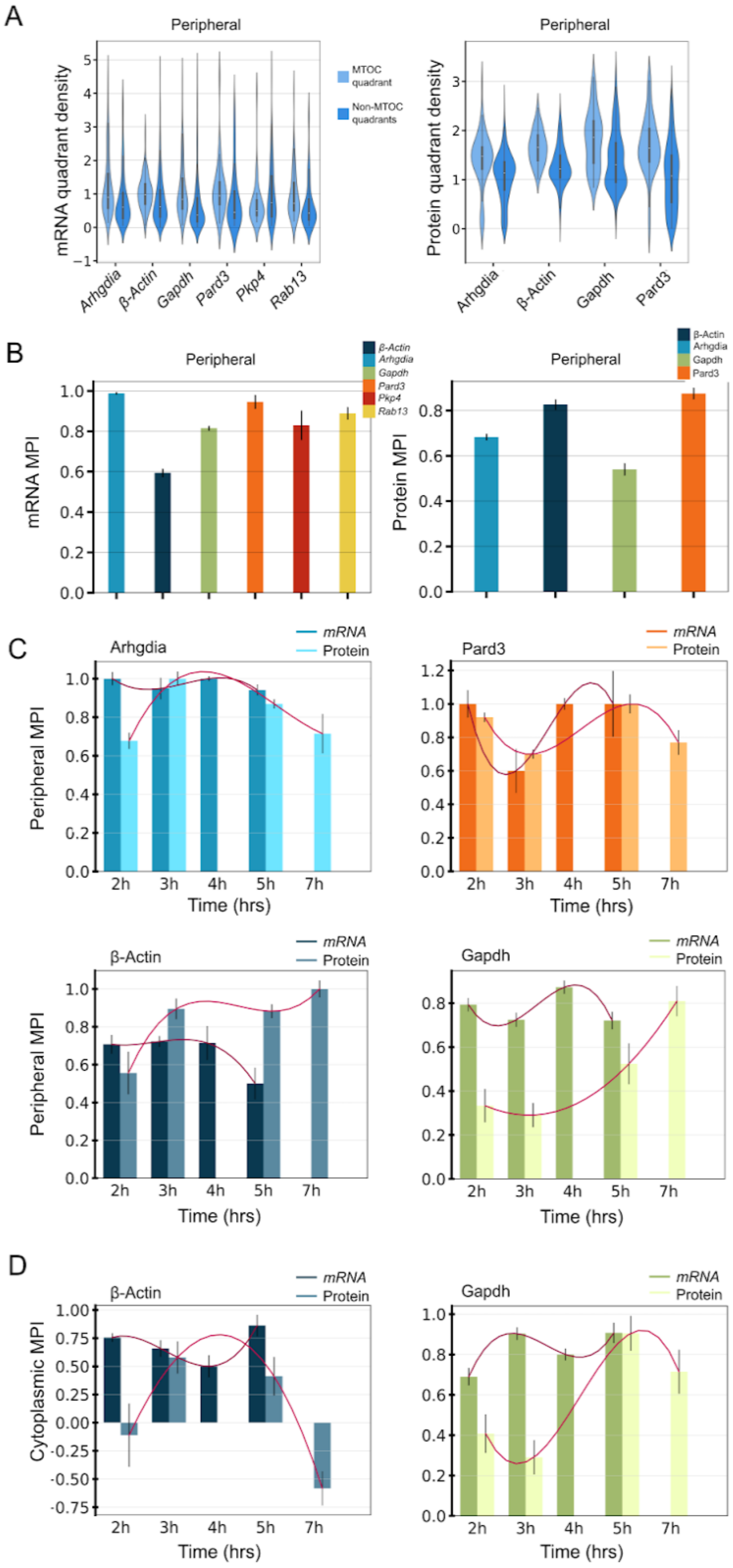

Figure S3, related to Figure 4 | Relationship between peripheral mRNA and protein distributions and MTOC position. (A) The cytoplasmic mRNA and protein relative density in non-MTOC containing quadrants (dark blue) and MTOC-containing quadrants (light blue), enrichment in the MTOC-containing quadrant is defined by differences of means of these distributions. (B) Comparison of MPI values for mRNAs and proteins in peripheral populations (all time points). (C) Comparison of peripheral MPI dynamics for β-Actin, Gapdh

Arhgdia and Pard3 mRNA-protein pairs. (D) Comparison of cytoplasmic MPI dynamics for Gapdh and β-Actin mRNA-protein pairs. Bar graphs in (B) show median and standard deviation from the median error bars for 100 bootstrapped MPI estimates. Graphs in (C) and (D) show median surrounded by envelope indicating standard deviation from the median error bars of 100 bootstrapped estimates fitted to cubic splines.

Figure S4, related to Figure 4

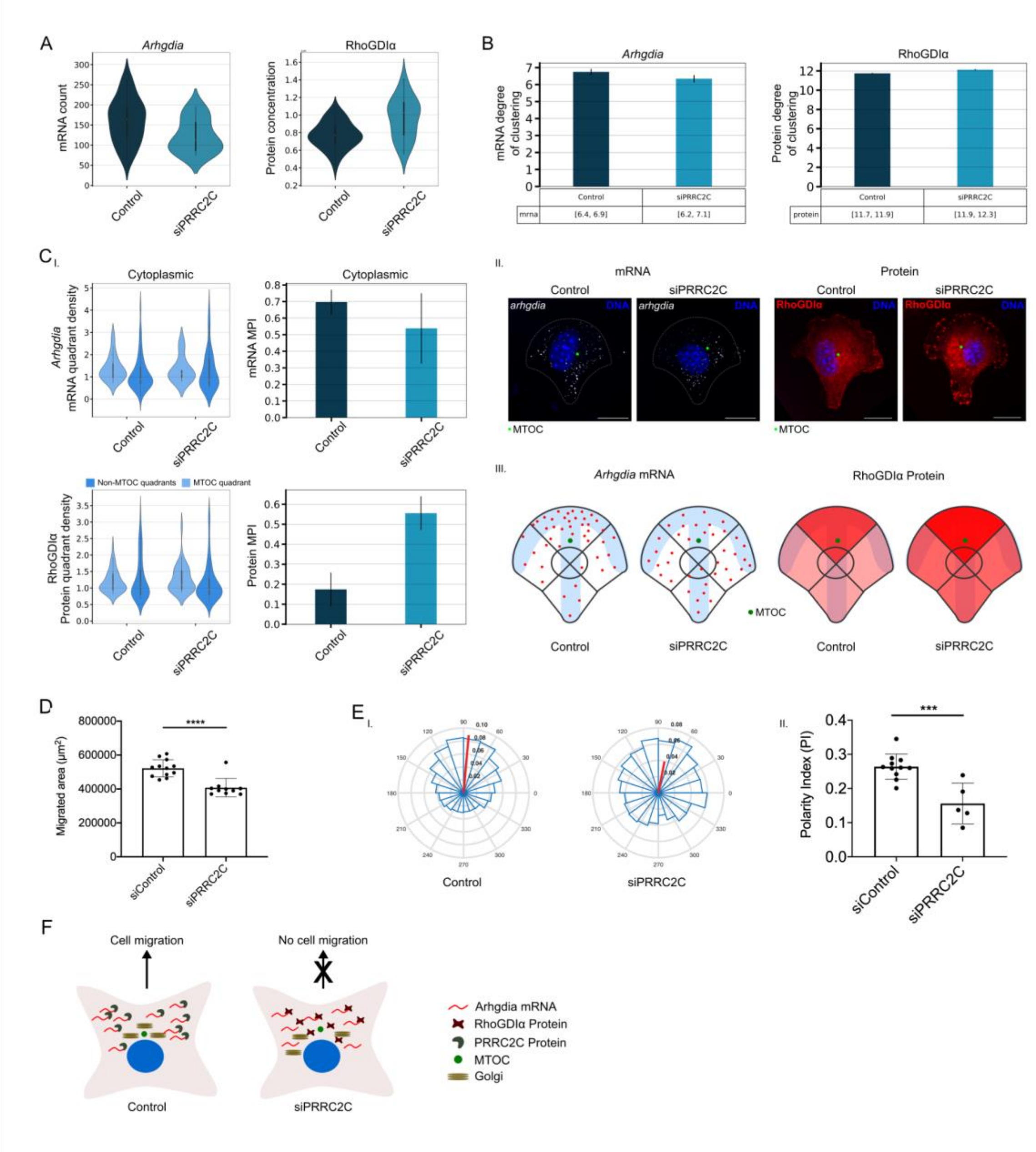

Figure S4, related to Figure 4 | Depletion of PRRC2C, an *Arhgdia* binding protein, decreases *Arhgdia*'s MTOC polarity and causes defects in cell migration. (A) Total *Arhgdia* mRNA count and RhoGDIα (Arhgdia protein) concentration in control and PRRC2C siRNA depleted 3T3 fibroblasts at 3 h post induction of polarization. (B) Comparison of degree of clustering for *Arhgdia* mRNA and protein (RhoGDIα) at 3 h (log values shown after scaling by natural log for mRNAs and proteins). Error bars show the standard error of the mean. (C) The cytoplasmic *Arhgdia* mRNA and protein (RhoGDIα) quadrant density and MPI values in control and PRRC2C-depleted cells (i). Representative images are shown for *Arhgdia* smFISH and RhoGDIα

immunofluorescence, as well as DAPI stain, scale bar 10 µM (ii). The cartoon in (iii) represents the cytoplasmic MPI values in control and PRRC2C-depleted cells for *Arhgdia* and RhoGDIα. (D) Quantification of migrated area from siControl and siPRRC2C transfected HUVECs after 16 hours of migration (n=1; mean ± SD of 3 replicates/condition). (E) Rose plots showing the polarization angles distribution of 1st to 4th rows from siControl (n=2; mean ± SD of 11 replicates) and siPRRC2C (n=2; mean ± SD of 5 replicates) transfected HUVECs. Bar plots with individual data points representing the polarity index of each replicate from siControl (n=2; mean ± SD of 11 replicates) and siPRRC2C (n=2; mean ± SD of 5 replicates) transfected HUVECS. (F) Model describing the effect of PRRC2C depletion on *Arghdia* MTOC polarity, subcellular localization and degree of clustering, RhoGDIα translation and subsequent cell migration.

Figure S5, related to Figure 4

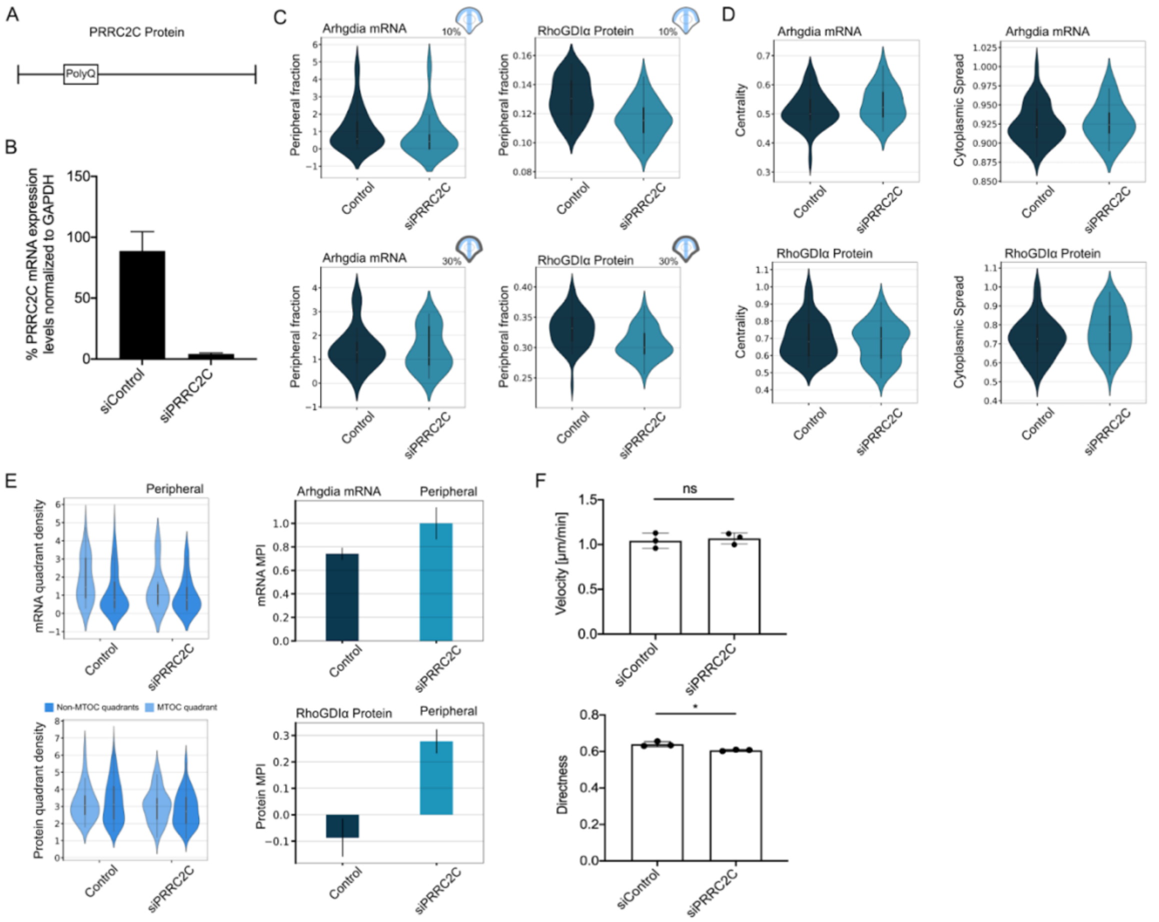

Figure S5, related to Figure 4 | Depletion of PRRC2C, an *Arhgdia* binding protein, decreases *Arhgdia*'s MTOC polarity and causes defects in cell migration. (A) The PRRC2C protein contains a PolyQ motif. (B) Quantification of PRRC2C mRNA levels normalized to GAPDH from siControl (n=1; mean ± SD of 2 replicates) and siPRRC2C (n=1; mean ± SD of 3 replicates) transfected HUVECs. (C) Distributions of fractional values for peripheral enrichment of *Arhgdia* mRNA and protein (RhoGDI) at 3 h post induction of polarization, at 10% and 30% of the radial distance from the plasma membrane to the nucleus. (D) Centrality and cytoplasmic spread of *Arhgdia* transcript and protein (RhoGDI) at 3 h post induction of polarization. The centrality is defined as a statistics measuring the evenness of a molecule spread across the cell, with the value 1 for even distribution. (E) The peripheral *Arhgdia* mRNA and protein (RhoGDI) quadrant density and MPI values in control and PRRC2C-depleted cells. Error bars show the standard error of the mean. (F) Quantification of cell velocity from siControl and siPRRC2C transfected HUVECs during 16 hours of migration (n=1; mean± SD of 3 replicates/condition) (top) and of migration directness from siControl- and siPRRC2C-transfected HUVECs during 16 hours of migration (n=1; mean± SD of 3 replicates/condition) (bottom).

Figure S6, related to Figure 5

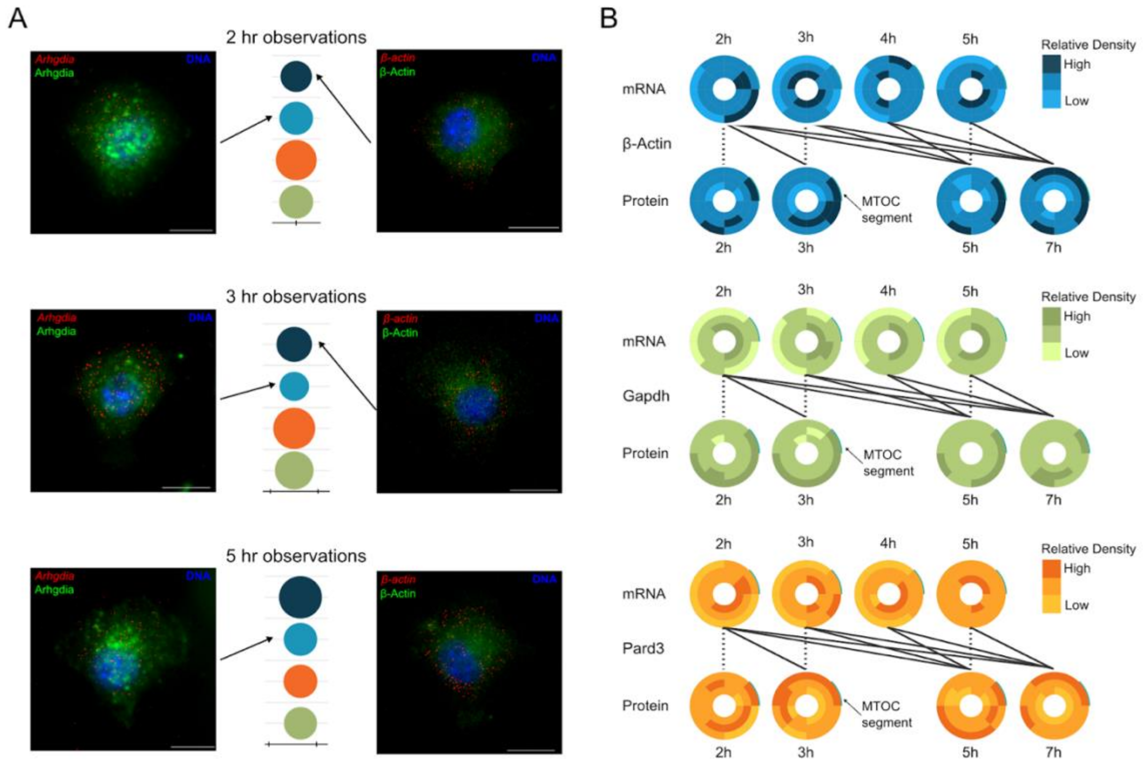

Figure S6, related to Figure 5 | Interdependency of localization dynamics for corresponding mRNAs and proteins. (A) Representative images for Arhgdia and β-Actin where smFISH and IF were performed in the same cell at 2 h, 3 h and 5 h post induction of polarization, scale bar 10 μM. (B) Forward-leading' time point pairs, defined as t1<t2 (connected by solid lines), were used for calculating Colocalization Score values. The 'forward-leading' time points were chosen as we considered the additional time for translation to occur once the mRNA is localized. Cytoplasmic density maps representing relative density vectors based on fine-grained quantization for Gapdh, Pard3 and β-Actin mRNA and protein for each time point are shown. Celular regions are dark blue if the local relative density is greater than the mean density of all segments in the cytoplasm + standard deviation, light blue if the local relative density is smaller than the mean of all segments in the cytoplasm - standard deviation, intermediate values correspond to the relative density within the [-σ, σ]interval. The MTOC-containing quadrant is highlighted by a thin turquoise stripe.

Figure S7, related to Figure 6

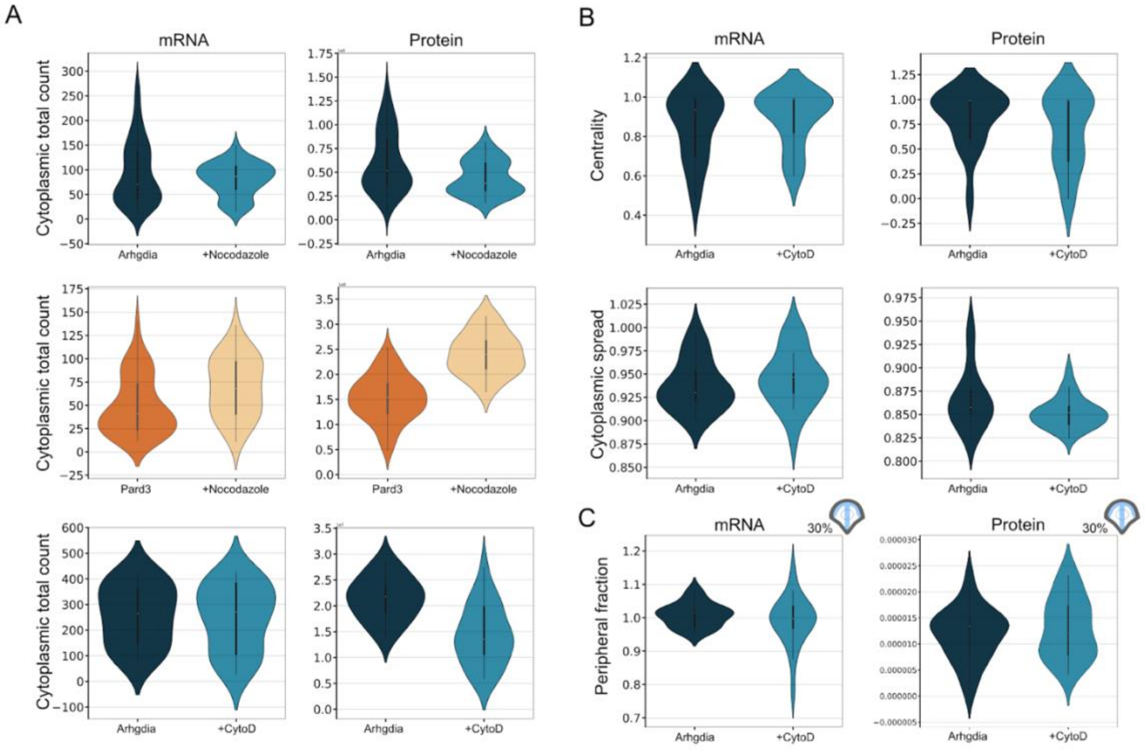

Figure S7, related to Figure 6 | Effects of cytoskeleton disturbance on mRNA-protein centrality, cytoplasmic spread, mRNA and protein counts and peripheral fraction. (A) + (B) Effects of nocodazole and CytoD treatment on cytoplasmic total count, centrality and cytoplasmic spread descriptors for Arhgdia and Pard3 mRNAs and proteins at 3-5 h time-points. Error bars show the standard error of the mean. (C) The peripheral fraction of Arhgdia transcript and protein (at 3 and 5 h combined) were calculated similarly to Figure 6C.

Figure S8, related to Figure 7

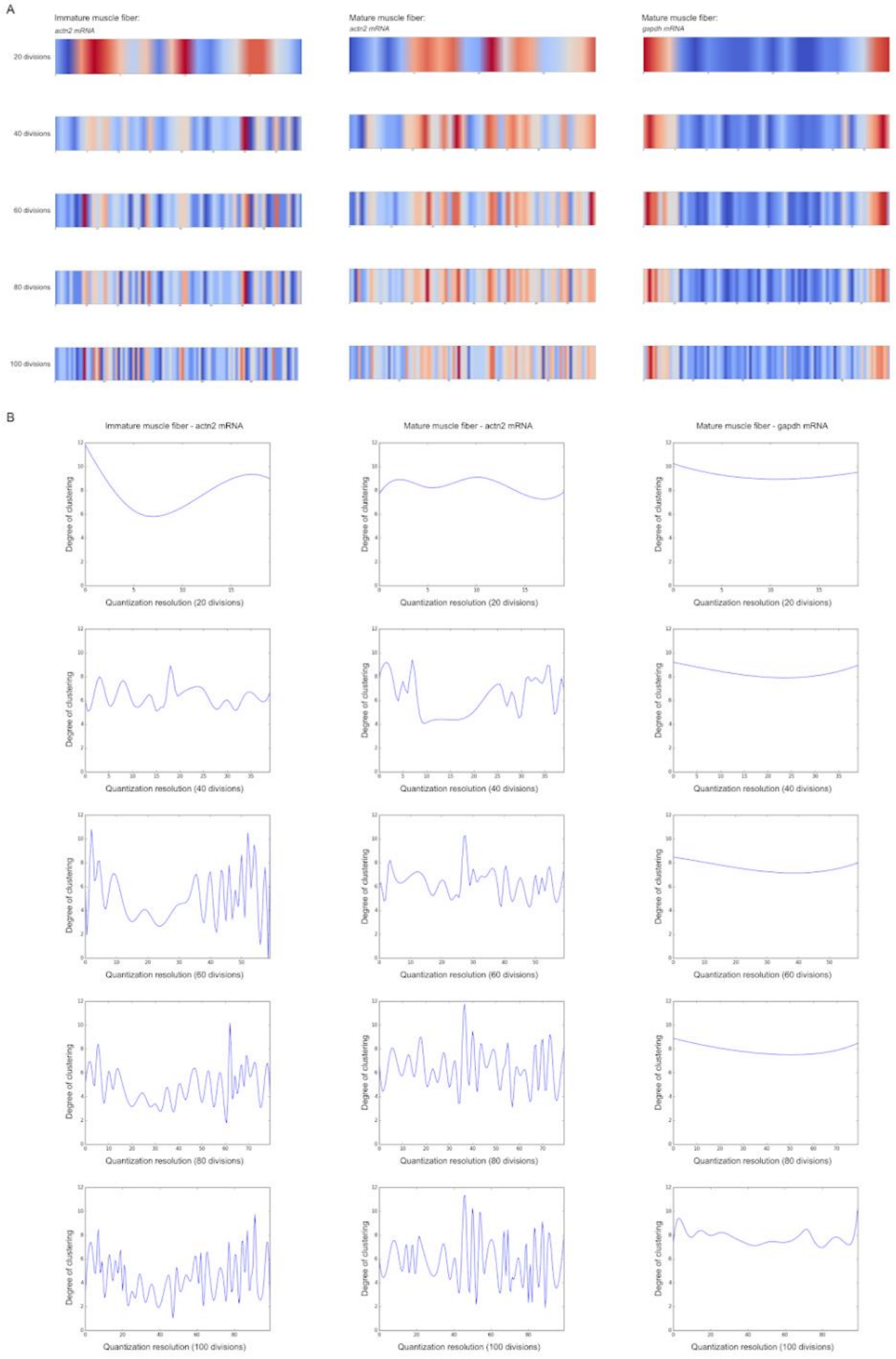

Figure S8, related to Figure 7 | Sarcomeric mRNAs cluster in a striated pattern in differentiated myofibers. (A) + (B) The mRNA local density was computed between two nuclei. Each cell was quantized in vertical quadrants and relative concentration of mRNA in each quadrant was computed by normalizing the counts by the relevant surface. A wave-like clustering is observed for *actn2* in mature compared to immature fibers. No clustering is observed for *Gapdh*.
