# Supplementary material for: Interrogating RNA and protein spatial subcellular distribution in smFISH data with DypFISH
Source: Cell Rep Methods. 2021 Sep 13;1(5):100068. doi: 10.1016/j.crmeth.2021.100068 (PMC9017151; doi:10.1016/j.crmeth.2021.100068)
Supplement: Document S2. Article plus supplemental information [file mmc5.pdf]

Cell Reports
Methods

Article

Interrogating RNA and protein spatial subcellular distribution in smFISH data with DypFISH

Graphical abstract

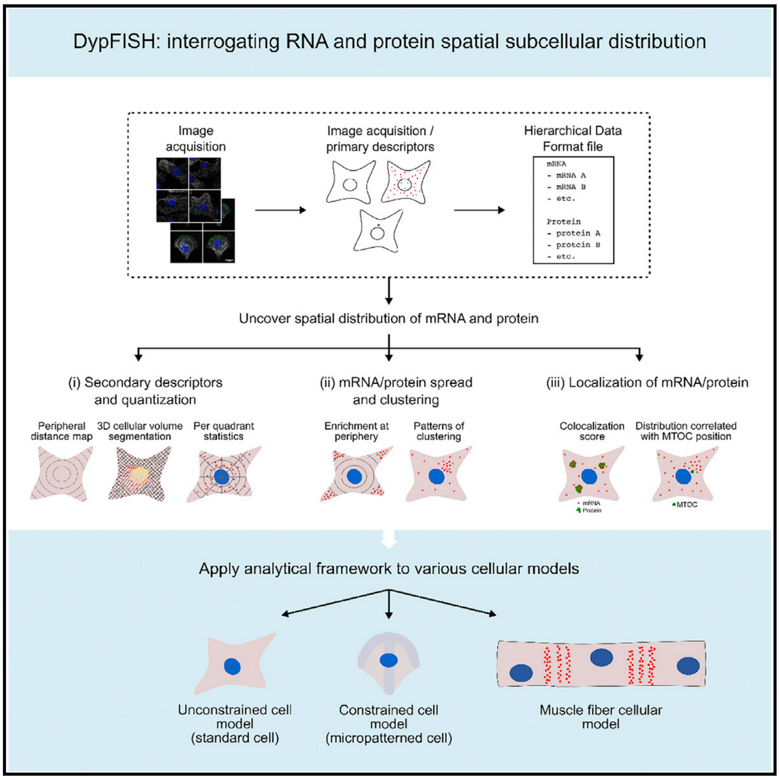

Highlights

- Micropatterning of cells enhances reproducibility of mRNA subcellular distributions
- The DypFISH method is suited to study clustering patterns of mRNA and protein
- By using DypFISH, mRNA peripheral enrichment and in relation to the MTOC was found
- DypFISH revealed colocalization patterns of biomolecules in relation to each other

Authors

Anca F. Savulescu, Robyn Brackin, Emmanuel Bouilhol, ..., Edgar R. Gomes, Macha Nikolski, Musa M. Mhlanga

Correspondence

ankasi100@gmail.com (A.F.S.),
macha.nikolski@u-bordeaux.fr (M.N.),
belenus@mhlangalalab.org (M.M.M.)

In brief

Savulescu et al. present DypFISH, a method to quantitatively investigate the subcellular spatial localization of RNA and proteins. DypFISH introduces a range of analytical techniques and statistics to interrogate single-molecule RNA FISH data in combination with protein immunolabeling.

Savulescu et al., 2021, Cell Reports Methods *1*, 100068
September 27, 2021 © 2022 The Authors.
https://doi.org/10.1016/j.crmeth.2021.100068

CellPress

Cell Reports Methods

CellPress
OPEN ACCESS

Article

Interrogating RNA and protein spatial subcellular distribution in smFISH data with DypFISH

Anca F. Savulescu,[1,*] Robyn Brackin,[5] Emmanuel Bouilhol,[2,7] Benjamin Dartigues,[2] Jonathan H. Warrell,[6] Mafalda R. Pimentel,[4] Nicolas Beaume,[1] Isabela C. Fortunato,[4] Stephane Dallongeville,[3] Mikaël Boulle,[8,9] Hayssam Soueidan,[2] Fabrice Agou,[8,10] Jan Schmoranzer,[5] Jean-Christophe Olivo-Marin,[3] Claudio A. Franco,[4] Edgar R. Gomes,[4] Macha Nikolski,[2,7,*] and Musa M. Mhlanga[11,12,13,14,*]

[1]Division of Chemical, Systems & Synthetic Biology, Institute for Infectious Disease & Molecular Medicine, Faculty of Health Sciences, University of Cape Town, 7295 Cape Town, South Africa
[2]Université de Bordeaux, Bordeaux Bioinformatics Center, 33000 Bordeaux, France
[3]Unite D'Analyse D'Images Biologiques, Institut Pasteur, 75015 Paris, France
[4]Instituto de Medicina Molecular, Faculdade de Medicina Universidade de Lisboa, 1649-028 Lisbon, Portugal
[5]Advanced Medical Bioimaging, Charité – Universitätsmedizin, 10-117 Berlin, Germany
[6]Molecular Biophysics & Biochemistry, Yale University, New Haven, CT 06520, USA
[7]Université de Bordeaux, CNRS, IBGC, UMR 5095, 33077 Bordeaux, France
[8]Chemogenomic and Biological Screening Core Facility, C2RT, Department of Structural Biology and Chemistry, Institut Pasteur, 25 rue du Dr. Roux, 75724 Paris Cedex 15, France
[9]Université de Paris, Sorbonne Paris Cité, Paris, France
[10]Department of Structural Biology and Chemistry, URA 2185, Pasteur Institute, Paris, France
[11]Radboud Institute for Molecular Life Sciences (RIMLS), Radboud University Medical Center, 6525 GA Nijmegen, the Netherlands
[12]Epigenomics & Single Cell Biophysics Group, Department of Cell Biology, FNWI, Radboud University, 6525 GA Nijmegen, the Netherlands
[13]Department of Human Genetics, Radboud University Medical Center, 6525 GA Nijmegen, the Netherlands
[14]Lead contact
*Correspondence: ankasi100@gmail.com (A.F.S.), macha.nikolski@u-bordeaux.fr (M.N.), belenus@mhlangalalab.org (M.M.M.)
https://doi.org/10.1016/j.crmeth.2021.100068

MOTIVATION Subcellular spatial localization of RNAs and proteins, in addition to their expression levels, is widely acknowledged to be important for characterization of cell types and states. Alongside developments in single-cell RNA sequencing (scRNA-seq) techniques, there is a growing need to develop technologies that are able to provide quantitative information on the spatial location of molecules on a single-cell level. Thus, we developed DypFISH, an experimental and computational approach providing quantitative spatial measurements of molecules on a subcellular level, including clustering statistics, their location in relation to specific organelles, and colocalization between transcripts and their corresponding proteins. DypFISH provides a versatile toolbox and aims to enable the investigation of subcellular localization of molecules at fine-grained spatial resolution in single cells for a variety of cell types and topologies.

SUMMARY

Advances in single-cell RNA sequencing have allowed for the identification of cellular subtypes on the basis of quantification of the number of transcripts in each cell. However, cells might also differ in the spatial distribution of molecules, including RNAs. Here, we present DypFISH, an approach to quantitatively investigate the subcellular localization of RNA and protein. We introduce a range of analytical techniques to interrogate single-molecule RNA fluorescence *in situ* hybridization (smFISH) data in combination with protein immunolabeling. DypFISH is suited to study patterns of clustering of molecules, the association of mRNA-protein subcellular localization with microtubule organizing center orientation, and interdependence of mRNA-protein spatial distributions. We showcase how our analytical tools can achieve biological insights by utilizing cell micropatterning to constrain cellular architecture, which leads to reduction in subcellular mRNA distribution variation, allowing for the characterization of their localization patterns. Furthermore, we show that our method can be applied to physiological systems such as skeletal muscle fibers.

This is an open access article under the CC BY license (http://creativecommons.org/licenses/by/4.0/).

INTRODUCTION

The need to incorporate subcellular spatial information of central dogma molecules into traditional omics approaches has led to the call for spatially resolved omics of various kinds (Crosetto et al., 2015). This has become more urgent as projects such as the Human Cell Atlas use technologies such as single-cell RNA sequencing (scRNA-seq) to characterize subtypes of cells on the basis of their molecular signatures by counting the number of RNA transcripts. Although much progress has been made in spatially resolved transcriptomics (reviewed in Medioni and Besse, 2018; Strell et al., 2019), incorporating spatial information into omics approaches carries with it several difficulties, such as coping with biological heterogeneity and noise. Standard approaches such as RNA-seq to measure gene expression typically neglect spatial information. By revealing three-dimensional (3D) subcellular positions of RNA transcripts, cell states as defined by scRNA-seq might be altered or redefined, thus gaining higher resolution and information content.

The importance of subcellular localization of mRNA transcripts as a means to spatially and temporally restrict translation has been demonstrated in a wide variety of cell types (Bashirullah et al., 1998; Besse and Ephrussi, 2008; Jansen, 2001; Kloc et al., 2002; Martin and Ephrussi, 2009; Zappulo et al., 2017; Chouaib et al., 2020). Localizing specific mRNA transcripts to distinct subcellular localizations and subsequent local translation serves as an important determinant of protein localization and is often influential to cell function (Mardakheh et al., 2015; Moor et al., 2017; Zappulo et al., 2017). Although the number of fully characterized localized mRNAs is currently small, emerging studies have demonstrated that the RNA localization phenomenon is more widespread than previously assumed and might in fact be relevant for the majority of mRNA transcripts, including long non-coding RNAs (Bouvrette et al., 2018; Cabili et al., 2015; La Manno et al., 2018; Lécuyer et al., 2007; Moor et al., 2017; Sharp et al., 2011; Weis et al., 2013; Zappulo et al., 2017). Although a few studies have attempted to quantify spatial distribution of RNA (Briley et al., 2015; Park et al., 2012; Samacoits et al., 2018; Stueland et al., 2019; Yamagishi et al., 2009), the majority of studies investigating subcellular localization of numerous RNAs and proteins have been generally qualitative, lacking detailed quantitative approaches to systematically describe the positions of RNAs and proteins. They have typically been limited to systems in which spatial heterogeneity is controlled and subcellular partitions are easily defined, such as developmental models (Macdonald and Struhl, 1988; Tautz and Pfeifle, 1989), neuronal systems (Batish et al., 2012; Buxbaum et al., 2014), and polarized cells (Martin and Ephrussi 2009; Mili et al., 2008; Clatterbuck-Soper et al., 2017). Thus, a method that is able to capture and quantify dynamic RNA subcellular positioning, complementary to scRNA-seq, would help in the further identification and characterization of different cell states and subpopulations. In sum, to unravel the mechanisms of RNA spatial and temporal distribution, quantitative analytical tools that probe these relationships systematically need to be developed.

Here, we describe DypFISH, a spatially resolved high-throughput computational approach overcoming the aforementioned limitations by quantitatively measuring and analyzing the spatial colocalization of mRNA and protein distributions at fine-grained spatial resolution in single cells. On the experimental side, we make use of micropatterning of cells, which have been shown to lead to reproducible cell size and shape (reviewed in Théry, 2010), as well as spatial organization of organelles (Schauer et al., 2010), allowing the averaging of high numbers of cells. By using micropatterns, we are able to showcase the analysis of distinct patterns of subcellular localization of molecules of interest.

On the computational side, DypFISH introduces analytical techniques that allow joint analysis of discrete point-based small-molecule fluorescence *in situ* hybridization (smFISH) mRNA data and continuous intensity immunofluorescence (IF) protein data. The computational techniques include a generalized approach to identifying clustering patterns across different time points, an approach to identifying dependencies of mRNA and protein spatial distributions on organelle positioning, and an approach to identifying correlated spatial distributions between mRNA transcripts and their corresponding protein products globally and at specific subcellular locations. The DypFISH computational framework allows the uncovering of fine-grained aspects of localization patterns for different time points of RNA and proteins. DypFISH probes the dependencies uncovered through perturbation studies, thus allowing one to test for possible mechanisms underlying subcellular localization dynamics.

RESULTS

Analytical method

DypFISH is designed as a library for writing analysis scripts to study subcellular spatial distributions of molecules. The preliminary step for using the DypFISH framework is processing cellular images and storing the extracted information in an hdf5 file format, structured in a hierarchical way (Figure 1A, top box). In our case the relevant categories were the molecule name and type, acquisition time points, and the images themselves. Each image in the hdf file is required to have primary image descriptors (Figure 1A). These include cell and nucleus masks as well as position of a landmark of interest, in our case the microtubule organizing center (MTOC), which is indicative of the cell polarity; descriptors encoding the signal (spots' positions for smFISH images and signal intensities for IF images); and for 3D images, the 3D volume segmentation, as well as the zero level that indicates the last Z slice within the focus of the confocal microscope.

The DypFISH analytical framework consists of three major components and is presented schematically in Figure 1A.

(1) The first functionality of the DypFISH pipeline is computation of secondary descriptors (Figure 1Ai), stored in a separate hdf5 file. First, we compute the peripheral distance map composed of equally spaced isolines between the cell periphery and the nucleus. Second, each cell is subdivided into quadrants that are given a standard order, starting from the MTOC-containing quadrant. Finally, isolines and quadrants taken together allow the quantization of the cell in two and three dimensions and computation

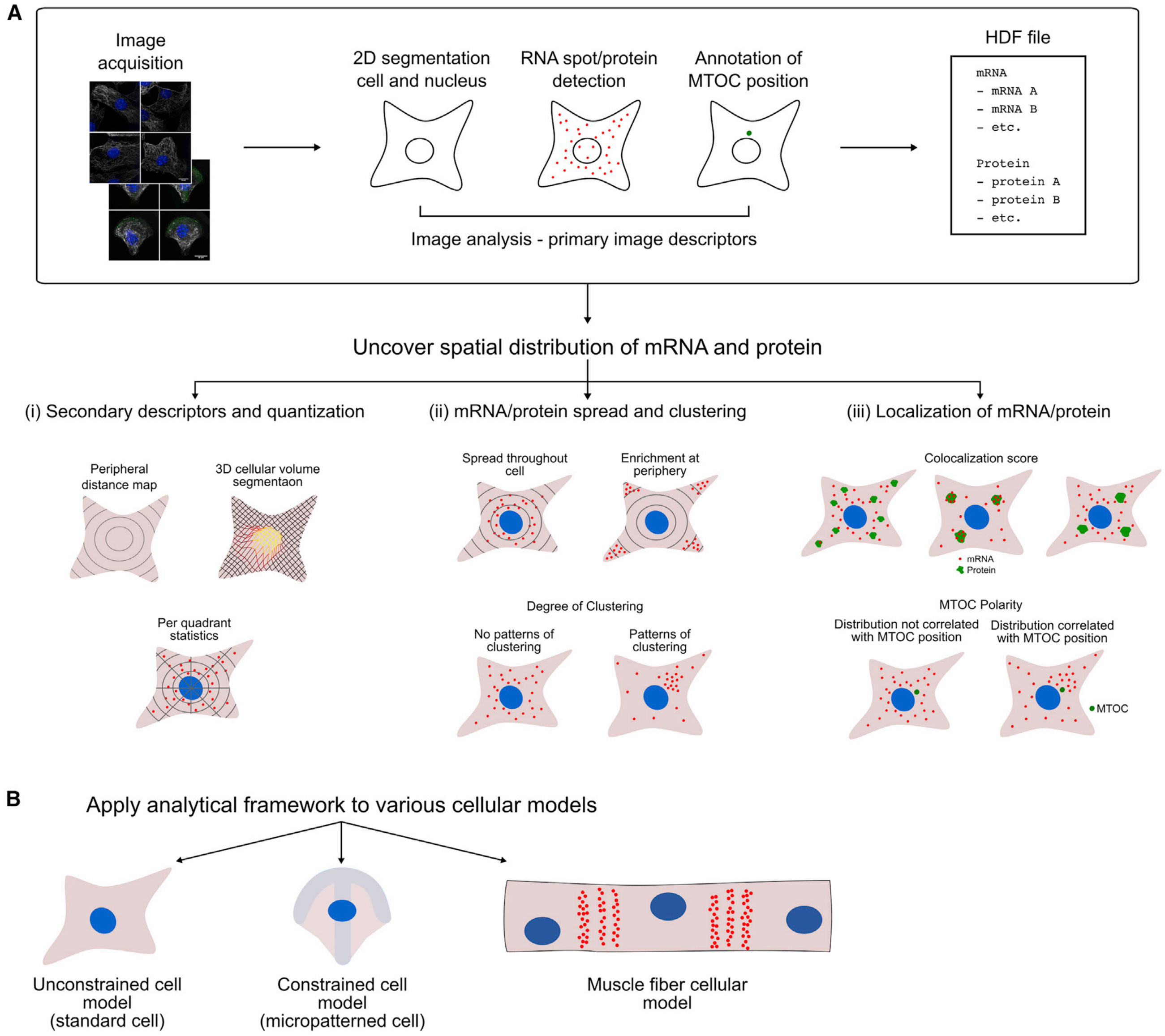

Figure 1. Analytical steps of the DypFISH method

(A) Steps in processing of smFISH and IF microscopy images, followed by various statistics computed by the DypFISH analytical framework either for (i) individual processed images or (ii) and (iii) sets of processed images.

(B) Cellular models in which the DypFISH analytical framework has been applied.

of relative signal densities for each of the segments (Figure 1Ai). Labeling of the MTOC enables cell-to-cell alignment, allowing for comparison of the resulting relative density vectors and segment-per-segment combination in the downstream statistical analyses.

(2) mRNA spread and clustering are statistics computed for sets of images based on secondary descriptors (Figure 1Aii). Among possible implemented analyses is the enrichment of molecule density at the cell periphery and, conversely, the measure of molecule spread toward the nucleus envelope (centrality). Basic entropy Kozachenko-Leonenko statistics, which measure how evenly the mRNA/protein signal is spread, are complemented by our generalized Ripley K function, which allows us to estimate the degree of clustering for mRNA and protein signal in a uniform way, despite differences in the nature of the signal (point-based and continuous).

(3) Specific analyses are implemented to study how mRNA/protein clusters are organized in the cells (Figure 1Aiii). First, labeling of the MTOC combined with cell quantization allows us to measure the enrichment of mRNAs and proteins in the MTOC-containing quadrants in relation to the rest of the cell. Second, colocalization score measures whether two different sets of images, for example

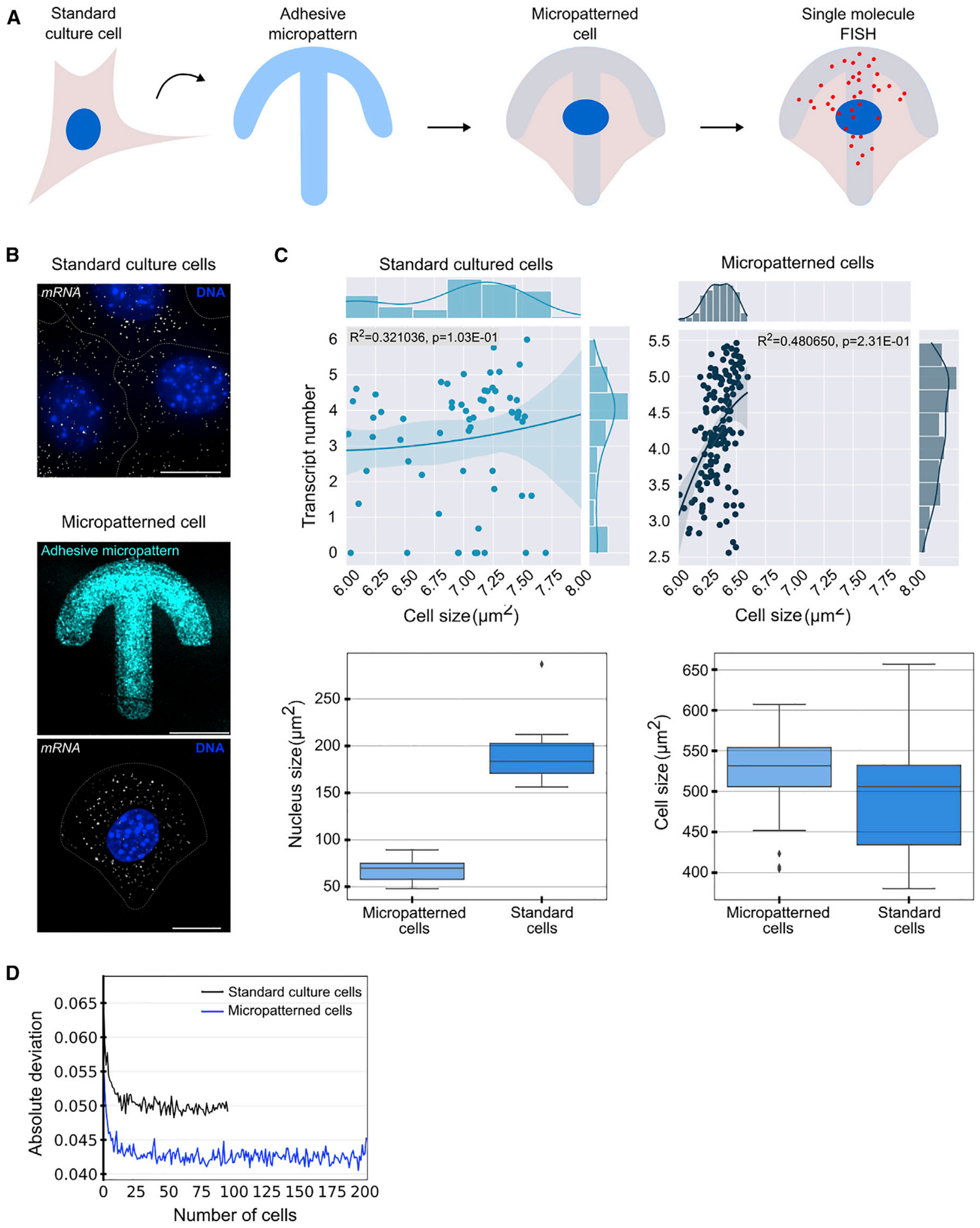

Figure 2. Reproducibility of mRNA and protein distributions in micropatterned cells

(A) Mouse fibroblasts were plated on fibronectin-coated micropatterns and induced to polarize by addition of serum. Cells were fixed and smFISH was performed to target mRNAs of interest.

(B) *Arhgdia* mRNA was visualized by using smFISH (gray) in standard and crossbow-shaped micropatterned mouse fibroblasts, micropatterns were visualized by coating with labeled fibrinogen (cyan), and DNA was stained with Hoechst (blue). Scale bar, 10 μm.

(legend continued on next page)

CellPress
OPEN ACCESS

mRNA at a given time point and its encoded protein at a later time point, exhibit similar patterns of their respective cellular density distributions.

The DypFISH analytical framework was applied to several cellular models, including those with constrained geometry—micropatterned mouse fibroblasts and skeletal muscle fibers—as well as standard unconstrained mouse fibroblasts for several analyses (Figure 1B).

Micropatterning of cells enhances reproducibility of mRNA subcellular distributions

We were interested in the ability to characterize subcellular positioning of RNA and protein in various cellular contexts and states. We selected a fibroblast system that allows the investigation of RNA positioning in relation to its polarity state (Mili et al., 2008) and combined it with micropatterning of cells to reduce cell-to-cell variability. We chose mRNA transcripts that had previously been identified as enriched in lamellipodia of fibroblasts upon polarization and cell migration (Hengst et al., 2009; Mili et al., 2008; Schmoranzer et al., 2009).

Micropatterning has been shown to lead to stereotypical localization of organelles, such as the centrosome, early endosomes, lysosomes, and the Golgi apparatus (Théry et al., 2005, Théry et al., 2006; Schauer et al., 2010). We were interested in establishing whether micropatterning can similarly reduce variation in mRNA spatial distributions and enable us to construct a quantitative framework for measuring reproducible subcellular spatial localization of RNA and protein. To this end, mouse fibroblasts were induced to polarize on crossbow-shaped micropatterns shown to be suitable for the study of polarizing cells (Théry et al., 2006; Schauer et al., 2010) and fixed at different time points after induction (Figure 2A). Each slide contained multiple 12 × 12 grids of crossbow-shaped micropatterns to which the cells adhered (Figure S1A). We developed an autonomous image acquisition and semi-automated image analysis pipeline (Figure 1A) that was able to scan each microfabricated slide and autonomously acquire images of individual cells. We used standard wide-field fluorescence microscopy, spinning-disk confocal microscopy, or a StellarVision microscope using synthetic aperture optics technology to acquire a 3D stack of images for each cell. SmFISH and IF were performed to label mRNAs and corresponding proteins of interest, respectively. We also labeled the microtubule (MT) cytoskeleton, the nucleus, and the micropattern. Representative images of the micropattern and smFISH are shown in Figure 2B. A complete list of the acquired images and descriptions of image acquisition characteristics is presented in Table S1.

To investigate the subcellular localization and colocalization patterns over time, we followed mRNAs and corresponding proteins in polarized fibroblasts at different time points (2, 3, 4, and 5 h for mRNA and 2, 3, 5, and 7 h for protein) after induction of polarization by serum. These time points were chosen as fibroblasts polarize over this timescale on crossbow-shaped micropatterns. To determine whether micropatterning leads to reduced heterogeneity in mRNA distributions, we compared fibroblasts grown in standard culture with those grown on micropatterns. Representative images of standard cultured and micropatterned cells are shown for the *Arhgdia* mRNA in Figure 2B.

First, we compared the reproducibility of *Arhgdia* mRNA distributions in standard cultured and micropatterned cells by using circular quantization in isolines from the periphery to the nucleus envelope. The absolute deviation of the quantized distribution of a randomly selected cell from a pooled average is reduced in micropatterned cells for all pool sizes up to ∼40 cells (Figure 2D). The error profiles are concordant with a previous study, which estimated that ∼20 micropatterned cells were necessary to establish reproducible organelle positions by using the AMISE metric (Schauer et al., 2010). We further investigated the effect of micropatterning on the volume-corrected noise measure *Nm* introduced in (Padovan-Merhar et al., 2015), which corresponds to the coefficient of variation of mRNA density corrected for the cell's volume. Following this study and others (Fomina-Yadlin et al., 2014; Gupta et al., 2012; Jain et al., 2013; Kempe et al., 2015), we modeled the relationship between transcript number and cell size by using polynomial regression, as demonstrated for *Arhgdia* mRNA (Figure 2C). Our data show reduction in transcript number variation between micropatterned cells in comparison with standard cultured cells, in line with Battich et al. (2015), in which constraining the cell phenotypic state resulted in reduction in cell-to-cell variability in cytoplasmic RNA concentration. The dispersion around the linear model fit line in the micropatterned cells as attested by the Pearson square coefficient r^2 (Figure 2C) is lower, leading to a lower *Nm* value (Figure S1C). This comparison revealed a tighter distribution of cell and nuclear sizes in the micropatterned cells, consistent with a mechanism to compensate for cell size fluctuations and maintain the concentration of mRNA in the cell, as proposed previously (Figure 2C; Padovan-Merhar et al., 2015).

Next, we investigated the profiles over time of the *Nm* for a series of mRNA transcripts including *Pkp4* and *Rab13*, which are enriched at the leading edge in polarized fibroblasts (Mili et al., 2008; Moissoglu et al., 2019), *Pard3*, which translates into the Par3 protein that regulates polarity in various cell types and is enriched in developing axons (Hengst et al., 2009; Schmoranzer et al., 2009), *β-Actin*, a well-studied localized mRNA in various cell types (Bassell et al., 1998; Buxbaum et al., 2014; Katz et al., 2012; Kislauskis et al., 1997; Park et al., 2012; Park et al., 2014; Yoon et al., 2016; and others), and *Gapdh*, which to the best of our knowledge is not known to localize to specific subcellular domains. We found for a number of transcripts a reduction in *Nm* over time, up to the 4 h time point (Figure S1B). These data indicated that micropatterns conveyed an advantage for quantitative analysis of RNA subcellular position over standard cell culture.

(C) Relationship between cell size and *Arhgdia* transcript copy number in standard cultured and micropatterned cells. Solid lines in upper graphs show the least-squares fit. Pearson square coefficient values R^2 are 0.48 and 0.32 for micropatterned and cultured cells, respectively. Lower graphs compare cell and nucleus size in the two conditions.
(D) Absolute deviation of *Arhgdia* mRNA distribution of a randomly selected cell from a pooled average of up to ∼40 cells for cultured and micropatterned cells.

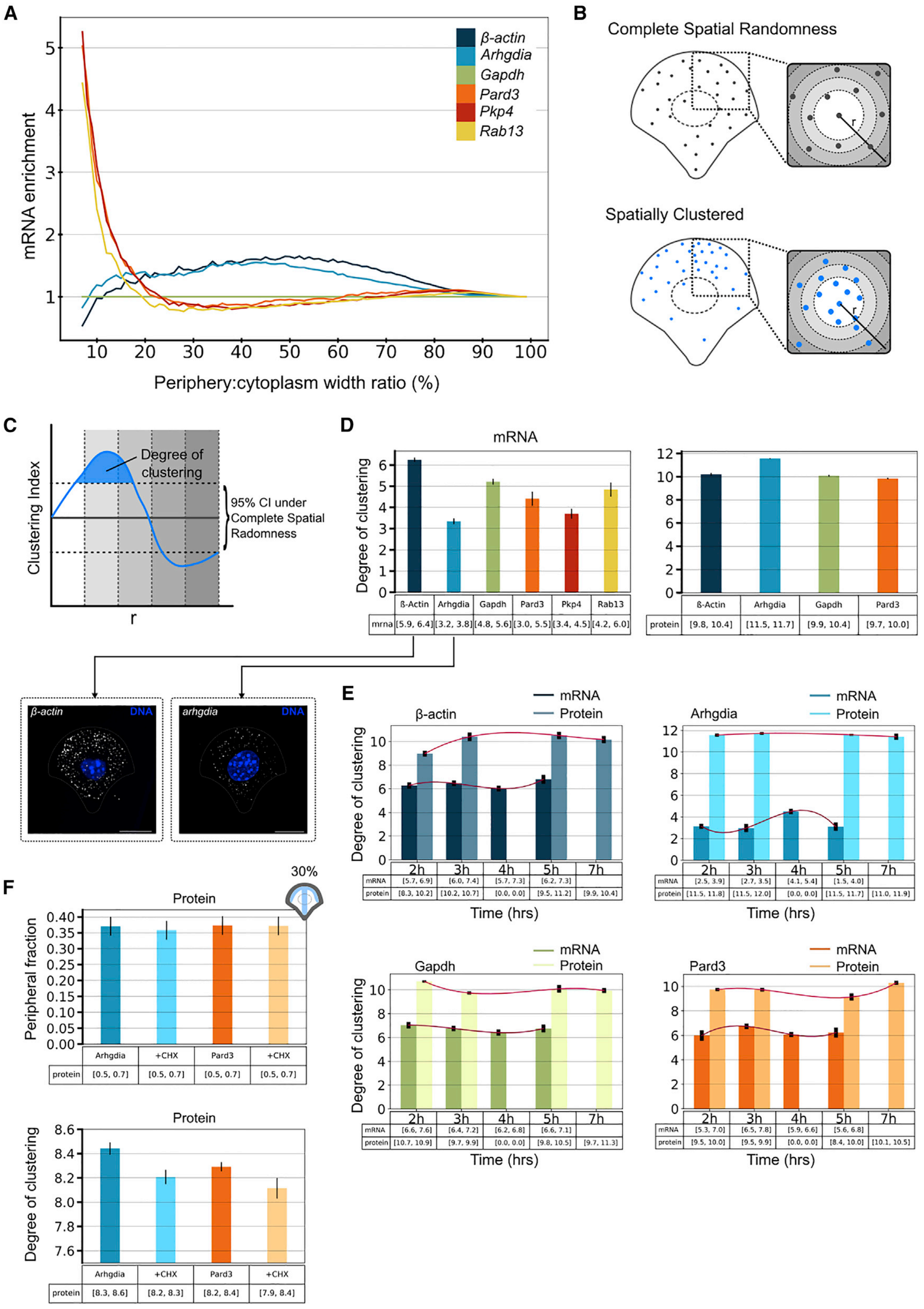

(legend on next page)

A subset of mRNAs and corresponding proteins shows peripheral enrichment and correlated clustering between time points

We investigated the joint localization patterns for mRNA and corresponding protein of four mRNA transcripts (*Arhgdia*, *Pard3*, *β-Actin*, and *Gapdh*) and the mRNA localization dynamics for the remaining two transcripts (*Pkp4* and *Rab13*). We developed a cell quantization method for computing local density statistics and used the MTOC cellular landmark to obtain consistent and alignable cellular segments. We initially characterized whether the mRNA and corresponding proteins, respectively, are enriched in the periphery of polarized cells. To do so, we calculated the fraction of cytoplasmic transcripts found at the boundary of the cell, within an area whose width is a fixed proportion of the radial distance to the nucleus edge (STAR Methods, "peripheral distance map"). Certain transcripts, including *Rab13* and *Pkp4*, as well as *Pard3*, are peripherally enriched for up to 20% of the radial distance (Figure 3A) in polarized cells on microfabricated patterns. Gapdh mRNA and protein distributions were used as controls, as neither the mRNA nor protein were expected to show patterns of enrichment (Mili et al., 2008; Figure S2A). Note that values in the extreme periphery are not stable, due to imprecision in segmentation of the cell mask. Representative smFISH images of *β-Actin* and *Gapdh* are shown in Figure S2B.

We next analyzed clustering of mRNAs and proteins by using a generalization of Ripley's K analysis (Lee et al., 2013; Ripley, 1977) that we first theoretically investigated in Warrell et al., 2016). Ripley's K is a commonly used algorithm to describe the extent of clustering of points, such as mRNAs (Figure 3B). For each mRNA spot and each distance d the number of transcripts lying within a sphere of radius d is counted. Spatial clustering can then be calculated by estimating the probability distribution of this function under a null hypothesis of complete spatial randomness (CSR) and comparing it with the function calculated from observed transcripts. We adjusted the algorithm (STAR Methods, "degree of clustering (Ripley-K)") on the basis of the generalized Ripley K function for evaluating the extent of clustering of both mRNA and protein spatial distributions. This was done by computing the degree of clustering, a unitless measure that can be used to compare clustering between different molecules and conditions. We summed the area where the normalized Ripley K function deviates from the 95% confidence interval of the random distribution (Figure 3C).

We evaluated the degree of clustering of all transcripts and proteins across all time points, revealing high overall values for all proteins and various values for the different mRNA transcripts (Figure 3D). Representative smFISH images for *β-Actin* and *Arhgdia* are shown in Figure 3D. We further calculated the degree of clustering at each individual time point post induction of polarization for *Arhgdia*, *Gapdh*, *β-Actin*, and *Pard3* mRNAs and proteins, and normalized it by mean for value's scale compatibility between mRNA and protein (Figure 3E). A clear peak in the mRNA profile followed by a peak in the corresponding proteins can be seen for Pard3, β-Actin, and Gapdh, suggesting temporal correlation (Figure 3E). A plausible interpretation of this temporal correlation might be local translation—clustering and translation of the transcript, yielding a high local concentration of protein that is observed as a cluster. To test this hypothesis, we used the translation inhibitor cycloheximide (CHX) and compared the peripheral enrichment and the degree of clustering of Arhgdia and Pard3 in inhibited and control cells (see description of image acquisition characteristics for CHX [Table S1] and STAR Methods). As expected, no significant reduction in peripheral enrichment for both *Arhgdia* and *Pard3* mRNAs was observed (Figure S2C), and there was a slightly increased degree of clustering for both mRNAs (Figure S2E). Taken together, these data indicate that *Arhgdia* and *Pard3* are localized to the periphery similarly in inhibited and control cells and cluster slightly more throughout the cytoplasm in inhibited cells. In contrast, a slight decrease was observed in peripheral enrichment and degree of clustering for both RhoGDIα (the protein translated from *Arhgdia*, to which we will refer to as Arhgdia for simplicity) and Pard3 proteins (Figures 3F and S2D). Altogether, these data indicate that in fibroblasts in which protein translation is inhibited, *Arhgdia* and *Pard3* mRNAs localize similarly to control cells; however, the reduced peripheral fraction and degree of clustering of the Arhgdia and Pard3 proteins hint toward potential local translation of these genes at the periphery. As transport of molecules is not expected to be affected in these cells, we do not anticipate the reduction in enrichment and clustering of Arhgdia and Pard3 to be attributable to impaired transport of the proteins.

Figure 3. Peripheral enrichment and clustering dynamics of mRNA-protein pairs

(A) Comparison of the enrichment of five mRNAs with respect to *Gapdh* mRNA in a peripheral cellular region whose width varies from 0% to 100% of the radial distance from the plasma membrane to the nucleus.

(B) Clustering is characterized by comparing observed transcript and protein distributions with complete spatial randomness. For mRNAs, the Ripley K function is estimated for an observed distribution and samples from a homogeneous Poisson process by counting the number of pairs of points lying within a radius r of each event.

(C) To compute the degree of clustering, we defined an estimator of the Ripley K function, called clustering index, by normalizing the observed Ripley K function to the 95th and 5th percentiles under the Poisson process. Significant clustering of mRNAs or proteins is found at radius r where the estimator function is over the 95% confidence interval under the CSR assumption. Values below the 95% confidence interval indicate the dispersion of the molecules. The degree of clustering is the area under the estimator's curve that is above the 95th percentile of the random distribution.

(D) Comparison of log degree of clustering for mRNAs and proteins (all time points, log values shown after scaling by natural log). Error bars show the standard error of the median. Representative single-molecule images for *β-Actin* and *Arhgdia* are shown. Scale bar, 10 μm.

(E) Clustering dynamics for four mRNA-protein pairs by using normalized degree of clustering. Zero values indicate the absence of protein or mRNA data for the given time point. Error bars show the standard error of the mean.

(F) Log degree of clustering (bottom graph), as well as enrichment of Arhgdia and Pard3 proteins in cells treated with CHX compared with control cells at 30% peripheral regions (top graph). Differences in degree of clustering were assessed by using a Mann-Whitney test. p values: Arhgdia versus Arhgdia + CHX = 0.2; Pard3 versus Pard3 + CHX = 0.04.

Localization of a subset of mRNAs and corresponding proteins shows correlation with the MTOC position

In the majority of polarized cell systems, the MTOC is positioned between the nucleus and the leading edge of the cell prior to migration or local cell growth (Gomes et al., 2005; Hale et al., 2011). Whether mRNAs and their corresponding proteins are subject to reorientation in relation to the MTOC is unknown. Having demonstrated that several mRNA transcripts and proteins exhibit peripheral enrichment and spatial clustering in polarized cells, we sought to determine whether these observations were related to MTOC positioning. We divided the cell into quadrants (Figure 4A), computed per-quadrant mRNA and protein relative density, and determined the MTOC localization within these quadrants (Figure 4A). We observed higher enrichment of all cytoplasmic mRNA transcripts in the MTOC-containing quadrant compared with the non-MTOC-containing quadrant, considering differences in their respective density (Figure 4B). Similarly, all peripheral transcripts at 30% of the radial distance, other than *Pkp4*, showed higher enrichment in the MTOC-containing quadrant compared with the non-MTOC-containing quadrant (Figure S3A). All cytoplasmic proteins were also found to be more enriched in the MTOC-containing quadrant compared with the non-MTOC-containing quadrant, with the exception of Pard3 (Figure 4B). All proteins showed enrichment in the MTOC-containing quadrant at 30% of the radial distance (Figure S3A).

On the basis of the above observations, we introduced an MTOC polarity index (MPI) to analyze MTOC-dependent enrichment in mRNA and protein distributions. This indicator lying between −1 and +1 is derived by normalizing the differences of signal concentration between the MTOC-associated quadrant and the other quadrants. Positive MPI values imply MTOC-dependent enrichment of RNA transcripts, negative values imply enrichment away from the MTOC, and a value of zero implies no detectable relationship with the MTOC. Representative smFISH images for *β-Actin* and *Pard3* are shown in Figure 4C.

We calculated the MPI for all transcripts and proteins, computing values for both the whole cytoplasmic population (Figure 4C), and the peripheral population at 10% radial distance (Figure S3B), by using all time points. All mRNAs as well as Gapdh, Arhgdia, and β-Actin proteins show high MPI scores in the cytoplasmic population. The low MPI score for Pard3 protein is in line with low MTOC enrichment for cytoplasmic Pard3 (Figure 4B). Similarly to the cytoplasmic transcripts, all mRNAs show high MPI scores in the peripheral population (Figure S3B). Additionally, we calculated MPI scores across time (Figures 4D, S3C, and S3D) and observed similar wave-like profiles for mRNA and corresponding protein (with a lag in time for the protein) for cytoplasmic Arhgdia, cytoplasmic and peripheral Pard3, and peripheral β-Actin, Arhgdia, and Gapdh, and cytoplasmic β-Actin and Gapdh to lesser extent (Figures S3C and S3D). This suggested a temporal association between mRNA, protein, and MTOC orientation.

We next applied the method presented above to examine the biological relevance of the spatial distribution of a given mRNA. We chose to focus on *Arhgdia* mRNA and characterized changes in its subcellular location, followed by measuring the effects of these changes on the localization and function of *Arhgdia*'s encoded protein. The *Arhgdia* transcript is translated into RhoGDIα, which regulates the Rho family of guanosine triphosphatases (GTPases) (reviewed in Garcia-Mata et al., 2011; Xie et al., 2017). We hypothesized that changes in the subcellular localization of *Arhgdia* would have an effect on the location and/or translation of the transcript to RhoGDIα and subsequent effects on Rho GTPases and related cellular processes, including cellular polarization and migration.

We first used a biochemical pull-down assay to pull down *Arhgdia* and identify its protein binding partners by mass spectrometry (Savulescu et al., 2020). By this process we identified a number of candidate protein-interacting partners. Of these we chose to focus on the PRRC2C protein, given that the PRRC2C subcellular localization at the ER (data not shown) is linked to cell polarity and in addition contains a polyQ motif (Figure S5A), suggesting interactions with RNA (Kunde et al., 2011; Lee et al., 2013; Langdon et al., 2018). We depleted PRRC2C (Figure S5B) to see whether it had convergent phenotypes with *Arhgdia*. We hypothesized that if PRRC2C was important to *Arhgdia* mRNA biology, this would result in quantitative changes detectable by DypFISH. PRRC2C-depleted cells displayed a lower *Arhgdia* mRNA count (Figure S4A), a slightly reduced peripheral fraction (Figure S5C), similar centrality and cytoplasmic spread (Figure S5D), and a slightly lower degree of *Arhgdia* mRNA clustering (Figure S4B). Depletion of PRRC2C also led to reduced peripheral MTOC enrichment, peripheral MPI score (Figure S5E), and a substantially reduced cytoplasmic MPI score (Figures S4C and S5E) for *Arhgdia*. A higher concentration of RhoGDIα was observed in PRRC2C-depleted cells (Figure S4A), suggesting a higher rate of translation in these cells as well as a decreased peripheral fraction and slightly increased centrality and cytoplasmic spread (Figures S5C and S5D). RhoGDIα retains Rho GTPases in an inactive, stable form in the cytosol, preventing their degradation (reviewed in Garcia-Mata et al., 2011). We hypothesized that an elevated concentration of RhoGDIα would induce an increased pool of inactive cytosolic Rho GTPases, leading to inhibition of cell migration. Consistent with our hypothesis, depletion of PRRC2C led to inhibition of migration of 3T3 mouse fibroblasts and human umbilical vein endothelial cells (HUVECs) in a wound assay (Figure S4D; Videos S1 and S2). We then tested whether the inhibition in migration was due to impaired velocity in PRRC2C-depleted cells. Velocity measurements of individual cells in the monolayer did not show differences between control and PRRC2C-depleted cells (Figure S5F); however, we observed a slight decrease in the ability of cells to maintain their directionality, termed directness, in PRRC2C-depleted cells (Figure S5F). Direction of migration is defined by the subcellular positions of the Golgi apparatus and MTOC in the cell, known as axial polarity (Ridley et al., 2003). To test whether it was abolished in the absence of PRRC2C, we performed an axial polarity assay (Carvalho et al., 2019). Loss of Golgi orientation in PRRC2C-depleted cells was observed (Figure S4E), suggesting the loss of axial polarity and, thus, migration defects. In conclusion, our data suggest that PRRC2C-depleted cells are unable to migrate properly.

Altogether, our data suggest that the absence of PRRC2C results in loss of *Arhgdia*'s enrichment in the MTOC vicinity and

CellPress
OPEN ACCESS

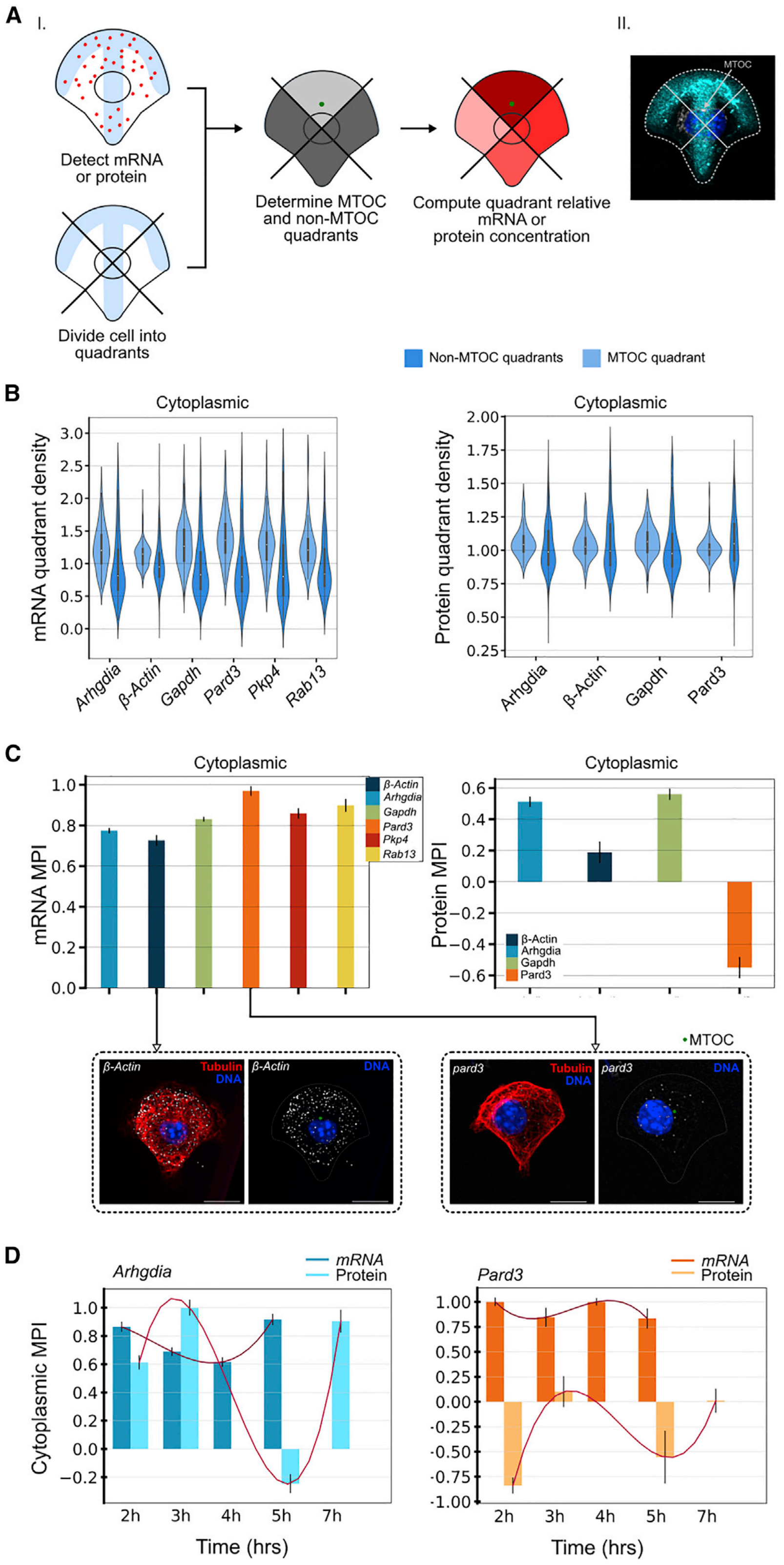

Figure 4. Relationship between cytoplasmic mRNA and protein distributions and MTOC position

(A) (i) Schematic of the MTOC correlative influence analysis and of the analysis to determine the MPI value. (ii) The MTOC position is annotated in projected 3D tubulin IF images. The MTOC polarity index (MPI) measures how frequently the relative concentration within the MTOC quadrants is higher than in the non-MTOC quadrants. MPI takes values between -1 and $+1$, with positive values indicating enriched mRNA or protein concentration in the MTOC quadrant and negative values indicating enrichment away from the MTOC quadrant.

(B) Cytoplasmic mRNA and protein relative density in non-MTOC-containing quadrants (dark blue) and MTOC-containing quadrants (light blue). Enrichment in the MTOC-containing quadrant is defined by differences in means of these distributions.

(C) MPI values for mRNAs and proteins in cytoplasmic populations (all time points). Representative smFISH images are shown for *β-Actin* and *Pard3* (right image), as well as the corresponding tubulin stain (left image). Scale bar, 10 μm.

(D) MPI dynamics for Arhgdia and Pard3 mRNA-protein pairs.

Bar graphs in (C) show median and standard deviation from the median error bars for 100 bootstrapped MPI estimates. Graphs in (D) show median surrounded by envelope indicating standard deviation from the median error bars of 100 bootstrapped estimates fitted to cubic splines.

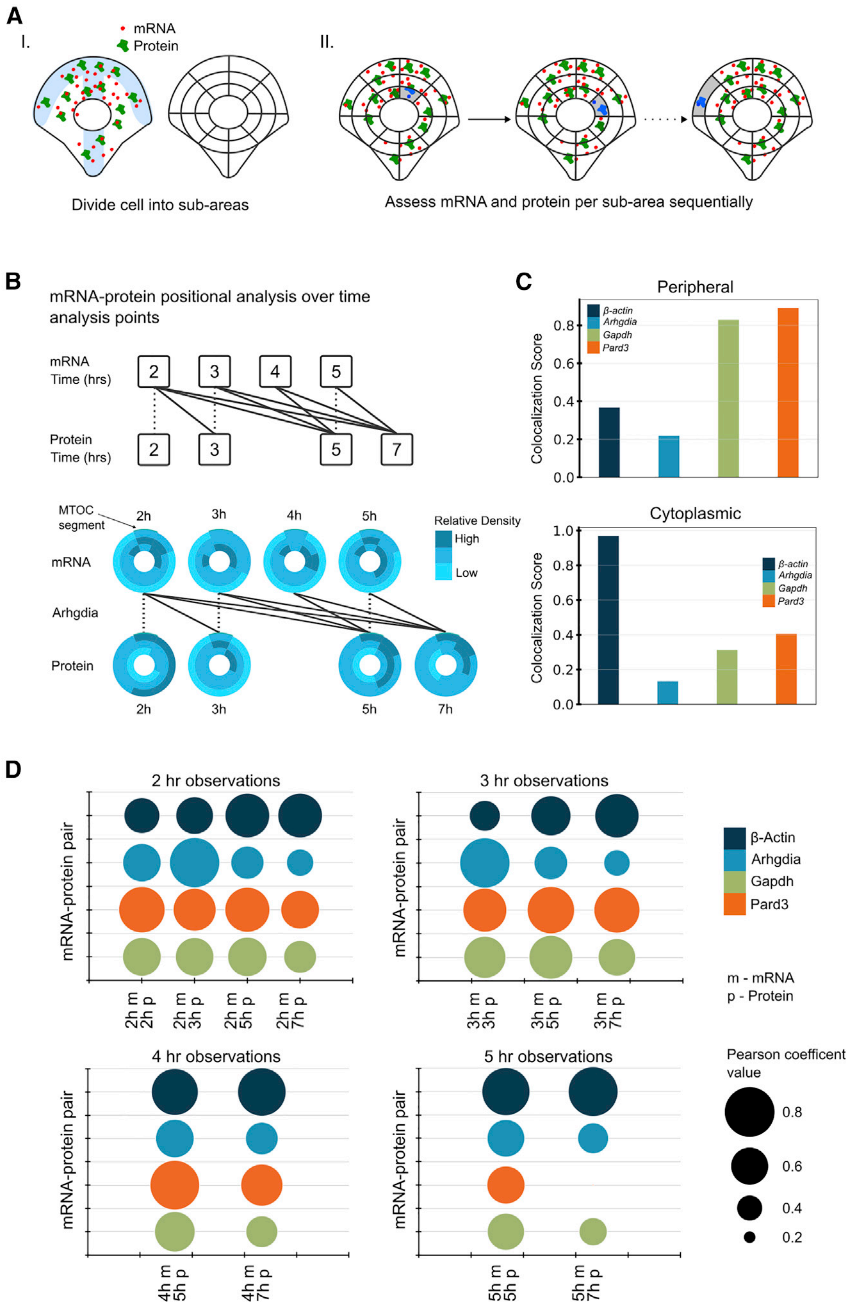

Figure 5. Interdependency of localization dynamics for corresponding mRNAs and proteins

(A) Colocalization score (CS) is a correlation between mRNA and protein spatial distributions for image acquisitions at several time points.

(B) Forward-leading time-point pairs, defined as $t_1<t_2$ (connected by solid lines), were used for calculating CS values. Cytoplasmic density maps representing relative density vectors based on fine-grained quantization for Arhgdia mRNA and protein for each time point are shown. Cellular regions are dark blue if the local relative density is greater than the mean density of all segments in the cytoplasm plus standard deviation, and light blue if the local relative density is smaller than the mean of all segments in the cytoplasm minus standard deviation; intermediate values correspond to the relative density within the $[-\sigma, \sigma]$ interval. The MTOC-containing quadrant is highlighted by a thin turquoise stripe.

(C) CS values were computed by using global correlations of all voxels/segments across the cytoplasmic area and local correlations across subsets of voxels/segments within peripheral regions.

(D) Correlations of relative density distributions for all cytoplasmic mRNA-protein pairs were observed for certain time points by using the fine-grained quantization scheme.

changes in its subcellular localization and degree of clustering pattern, as well as leading to seemingly elevated levels of translation, resulting in a higher concentration of RhoGDIα protein. We suggest that an elevated RhoGDIα concentration, as well as higher MPI score of the RhoGDIα protein in PRRC2C-depleted cells, leads to an imbalanced Rho GTPase cycle and subsequent inhibition in cell migration (Figure S4F). In summary, and most significantly, by using DypFISH, we unearthed variations in polarity and cell migration due to quantifiable changes in the MTOC-related subcellular localization of *Arhgdia* mRNA.

Colocalization of mRNA-protein distributions is consistent with MTOC-related patterns of localized translation

As previously described, several mRNA-protein pairs showed similarities in distributions of basic descriptors, clustering indices, and MTOC polarity indices. Correlations in spatial distributions at different time points could reflect spatially and temporally restricted translation (local translation) (Besse and Ephrussi, 2008) and/or separate localization pathways for mRNAs and proteins to common subcellular locations.

To investigate such scenarios, we performed fine-grained quantization of cells and were able to compute subcellular spatial distribution profiles of mRNAs and proteins (see STAR Methods, "colocalization score" and Figure 5Ai), visualized using density maps for each time point for four corresponding mRNA-protein pairs (Arhgdia, Gapdh, β-Actin, and Pard3) (Figures 5B and S6B).

For a pair of mRNA and protein spatial distributions, their colocalization is measured by the Pearson correlation of subcellular density vectors. We consider "high" or "low" colocalization for an mRNA/protein pair depending on the value of the Pearson correlation. High values (interdependent spatial distributions) might suggest local translation within specific subcellular localizations, although they do not rule out alternative mechanisms such as separate mRNA

and protein localization pathways with delayed protein transport.

To explore the consistency of potential correlations in spatial distributions between mRNA and the corresponding protein over different time points, we derived a colocalization score (CS), which is a value between 0 and 1, computed as the stochastic effect size based on the correlations between the subcellular distributions of mRNA and corresponding protein in a ranking across "forward-leading" time points (for details see STAR Methods, "colocalization score" and Figure 5B). These time points were chosen because we considered the additional time for translation to occur once the mRNA is localized.

The CS provides a global measure of whether the two molecules are distributed in a similar way in cells across all "forward-leading" time points as compared with the other time points. We measured the similarities of relative density vectors (1) globally across the cytoplasmic area by computing global CS values (Figure 5C) and (2) at the periphery using relative density vectors restricted to peripheral regions (Figure 5C). We observed correlated spatial distributions for cytoplasmic mRNA-protein pairs, with various temporal patterns among the different genes (Figures 5D and S6A). Generally, medium to high CS values were observed for all genes at the different time points (Figure 5D); however, we observed variability between the genes in CS values at various time points. For example, β-*Actin* mRNA at all time points showed high CS values with its corresponding protein at most time points, whereas the CS values for *Arhgdia* mRNA at late time points were generally lower than the values at early time points (Figure 5D).

Perturbation of various cytoskeletal components disrupts characteristic mRNA-protein localization and interdependency patterns, and hints at local translation

Our analysis revealed mRNA-protein colocalized distributions for certain time points, which suggested local translation. As we could not rule out independent localization of mRNA and corresponding proteins, we introduced two perturbations to inhibit potential transport pathways of the different molecules. We disrupted microtubule polymerization by using nocodazole (Figure 6A), which we reasoned would lead to disruption of the correlation with the MTOC of spatial distributions of selected mRNA and proteins, as well as potential loss of local translation. We selected Arhgdia and Pard3 mRNA-protein pairs to test this hypothesis, collected data at 3 h and 5 h after exposure to nocodazole, and compared it with untreated data from the equivalent time points.

We calculated the effects of nocodazole treatment on the total mRNA count (Figure S7A), the centrality of the transcript and protein (Figures 6B and S7B), and the 10% peripheral fraction (Figure 6C). *Arhgdia* centrality was increased upon treatment with nocodazole and its peripheral fraction was not significantly modified (Figures 6B and 6C), suggesting that *Arhgdia* mRNA and protein do not make exclusive use of the microtubule network for intracellular transport. Arhgdia protein centrality was slightly decreased and the peripheral fraction of the protein was reduced (Figures 6B and 6C), indicating a partial dependency on the microtubule network for subcellular transport. The centrality of *Pard3* mRNA was increased, whereas its peripheral fraction was slightly decreased upon treatment with nocodazole (Figures 6B and 6C), suggesting that Pard3 mRNA does not make exclusive use of the microtubule network for intracellular transport. A reduction was detected in the centrality of Pard3 protein in the presence of nocodazole (Figure 6B), and there was a slight increase in the peripheral fraction of the protein (Figure 6C). Interestingly, an increase in the *Pard3* mRNA and corresponding protein concentration was observed in the presence of nocodazole (Figure S7A).

Next, we probed the effects of nocodazole treatment on the relationship between the polarization of the cell and the subcellular localization of the mRNA transcripts and their corresponding proteins (Figures 6D and 6E). The MTOC enrichment of *Arhgdia* in nocodazole-treated cells compared with control cells was not substantially modified (Figure 6D) and the MPI of *Arhgdia* in these cells was slightly reduced (Figure 6E), indicating that the orientation of the mRNA subcellular localization was partially lost. An interesting effect was observed for *Pard3*, whereby no significant change was detected in the MTOC enrichment, with a slight increase in the MPI value for the mRNA in the nocodazole-treated cells (Figures 6D and 6E), indicating that the perturbation to the MTOC had the effect of tightening the relationship between the MTOC position and the RNA distribution. Representative smFISH images for *Arhgdia* in control and nocodazole-depleted cells are shown in Figure 6E.

We then probed the effects of nocodazole on mRNA-protein pairs by calculating local CS and maps for control and nocodazole-treated cells, restricted to the 3–5 h time points (Figure 6F). The interdependency of the Arhgdia mRNA-protein pair was disturbed, as observed in altered CS values for all time points in nocodazole-treated compared with control cells (Figure 6F). Nocodazole also affected the interdependency for the Pard3 mRNA-protein pair, mainly observed in the 3 h mRNA to 3 h protein and 5 h protein time points (Figure 6F).

To probe whether other cytoskeletal networks could influence RNA subcellular positioning, we treated the cells with the actin polymerization inhibitor cytochalasin D (cytoD) for 1 h before fixation of cells and compared FISH and IF data for the Arhgdia mRNA-protein pair. *Arhgdia* mRNA's centrality and cytoplasmic spread were slightly increased (Figure S7B), whereas the concentration of the mRNA was slightly lower on the periphery of cytoD-treated cells compared with control cells (Figure S7C). The centrality and cytoplasmic spread of Arhgdia protein were not significantly affected in the presence of cytoD (Figure S7B); however, the peripheral concentration of the protein was slightly decreased in the cytoD-treated cells (Figure S7C). Taken together, these data suggest that a lower Arhgdia mRNA peripheral concentration leads to reduced peripheral Arhgdia protein concentration or that mechanisms anchoring Arhgdia mRNA and protein to the periphery are lost in the presence of the drug.

Sarcomeric mRNAs cluster in a striated pattern in differentiated myofibers

To further validate Dyp-FISH, we sought a cellular model in which RNA subcellular localization and potential local protein translation linked to changes in cells' state could be interrogated. We focused on the unusually large multinucleated muscle cells

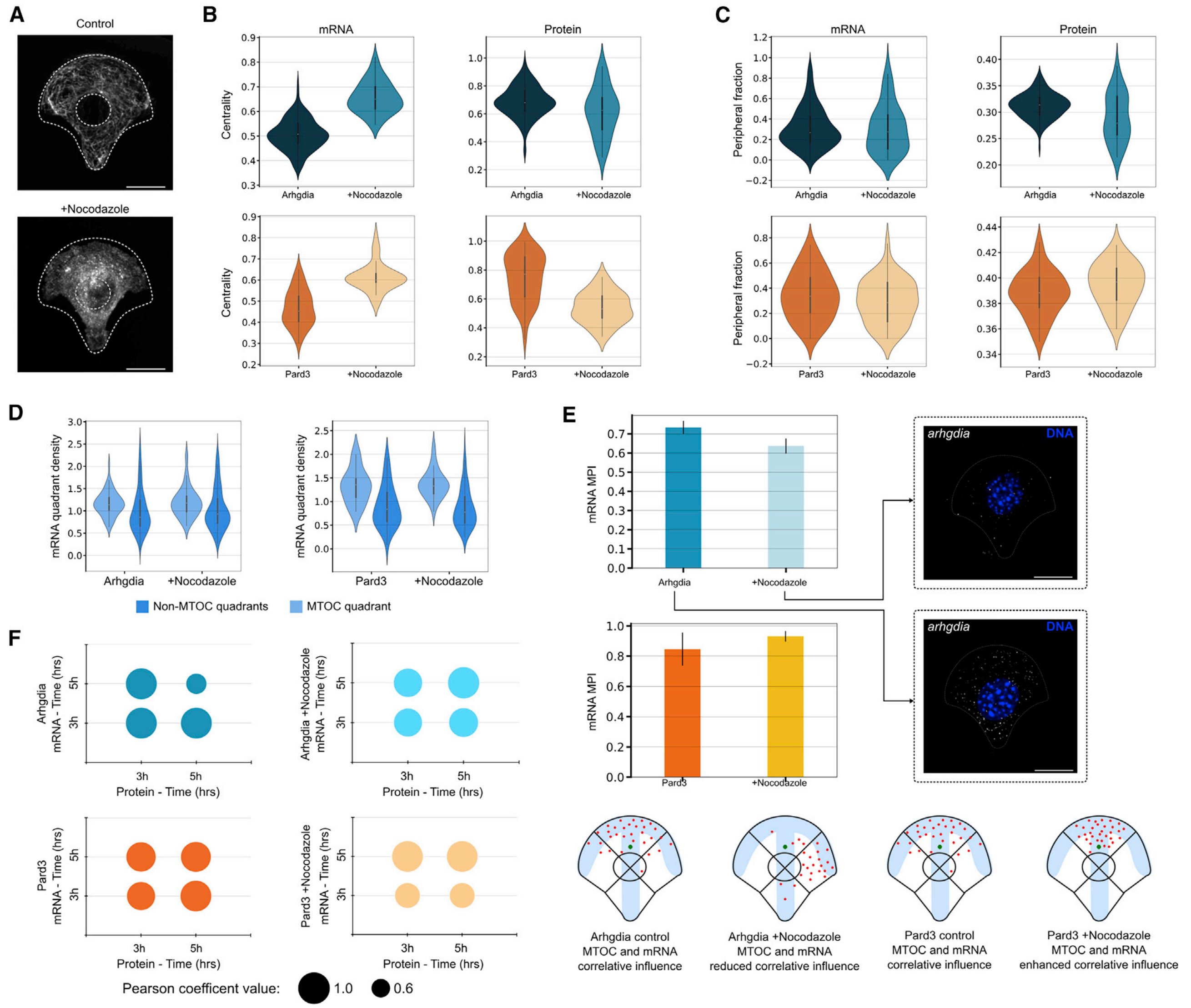

Figure 6. Effects of cytoskeleton disturbance on mRNA-protein localization and interdependent dynamics

(A) Nocodazole was added to cells seeded on micropatterns, inducing inhibition of microtubule polymerization (tubulin IF images). Scale bars, 10 μm.

(B) Centrality of Arhgdia and Pard3 transcripts and proteins (at 3 h and 5 h combined) is defined as a statistic measuring the evenness of a molecule spread across the cell, with the value 1 for even distribution. Error bars show standard error of the mean.

(C) The peripheral fraction of Arhgdia and Pard3 transcripts and proteins (at 3 h and 5 h combined) were calculated similarly to Figure 3B.

(D) mRNA quadrant density (MTOC enrichment profiles) for *Arhgdia* and *Pard3* shown for nocodazole-treated and control cells at 3 h and 5 h combined.

(E) MPI scores shown for control and nocodazole-treated cells for *Arhgdia* and *Pard3* transcripts at 3 h and 5 h combined. Representative smFISH images of Arhgdia in control and nocodazole-treated cells are shown. Scale bars, 10 μm. A cartoon representing the effect of nocodazole treatment on the MPI scores is shown in the bottom panel.

(F) Interdependent dynamics for Arhgdia and Par3 in the presence of nocodazole and in control cells.

termed myofibers that are formed by fusion of mononucleated cells, with the myonuclei being spaced at regular intervals, their main function being to generate mechanical force via contraction (Bruusgaard et al., 2003, 2006). Muscle contraction is achieved by the shortening of sarcomeres that are organized along the length of the myofiber and each sarcomere is flanked by a Z line, the site of anchoring of the actin filaments, resulting in the striation of the myofiber (Franzini-Armstrong and Peachey, 1981). We used skeletal muscle, given that the myofiber has an invariable tubular shape and a highly predictable cytoplasmic organization. *In vitro* differentiation of myofibers allows for high-resolution imaging throughout developmental stages, including the formation of patterned sarcomeres with well-defined Z-line striations, similar to what is observed in neonatal myofibers (Falcone et al., 2014; Pimentel et al., 2017; Vilmont et al., 2016). Thus, the size and regularity of myofiber architecture make them an excellent candidate to compute spatial distribution profiles of mRNAs and proteins.

We analyzed the distribution of an mRNA that encodes a protein found at the Z lines during muscle differentiation. We chose the *actn2* mRNA, which encodes for α-actinin, the main component of Z lines in relation to the sarcomeric Z lines (Figure 7A). The majority of the *actn2* mRNA was found in the vicinity of the Z line in mature myofibers (Figure 7B). To understand whether this depends on the developmental stage, we imaged immature myofibers in which the Z lines are less organized. We also probed the *Gapdh* mRNA distribution as a non-sarcomeric control in both immature and mature myofibers. The degree of mRNA proximity was lower in both cases, suggesting that possibly *actn2* mRNA localization precedes protein organization (Figure 7B) of the Z line. To address this question, we quantized each cell perpendicularly to the cell axis in vertical quadrants by using a number of various divisions (Figures 7C and S8A), similarly to sarcomeres' organization. The mRNA local density was computed in each quadrant by normalizing the counts by the relevant area (Figures 7C, S8A, and S8B). We observed a wave-like clustering for *actn2* in mature compared with immature fibers by using various divisions (Figures 7C, S8A, and S8B). No clustering was observed for *Gapdh* (Figures 7C, S8A, and S8B). Curiously, in adult mouse myofibers, it was recently described that *Gapdh* mRNA also clusters at the Z line, suggesting that myofiber hypertrophy alters mRNA clustering properties (Denes et al., 2021).

The highest degree of clustering was observed for *actn2* mRNA in mature myofibers when compared with the immature counterpart or with *Gapdh* mRNA. These data suggest that the *actn2* mRNA distribution specifically follows the respective protein organization instead of preceding it (Figure 7D). These data shed light on a long-standing question in the field and produce a basis of testable hypotheses for how *actn2* mRNA is directed to the Z line.

DISCUSSION

A wide variety of methods have been proposed for studying RNA localization with subcellular accuracy, including microscopy-based methods, such as those based on FISH (Battich et al., 2013; Chen et al., 2015; Lécuyer et al., 2007). We set out to develop a quantitative, reproducible, and reusable computational method for investigating the spatial distribution of RNA and protein. To achieve this, we hypothesized that the best showcase of our approach would be to use micropatterning of cells in order to reduce cellular heterogeneity and enhance the reproducibility of spatial distributions. We combined this with smFISH, IF labeling, and automated high-content imaging, as well as the development of our DypFISH computational framework. We revealed dependence of mRNA-protein localization and dynamics on MTOC orientation and cell polarization in polarized fibroblasts. Through perturbation studies, we have demonstrated DypFISH's ability to quantitatively detect changes in localization behavior, confirming both the robustness of the approach and its ability to test mechanistic hypotheses. This approach can be applied to other systems in which (1) there is a clear constrained architecture, such as the skeletal muscle shown in Figure 7, or/and (2) cellular landmarks can be stained, similarly to the MTOC landmark in our study.

RNA subcellular localization patterns and the role of the MTOC in subcellular spatial distribution

The association of different mRNA species with MTOC orientation indicates specific subcellular distribution patterns in accordance with broader processes, which are also responsible for controlling the nucleus and MTOC relative orientation during polarization (Kim et al., 2014; Razafsky et al., 2014). We posed the question as to whether proteins translated from mRNAs that consistently localize to similar subcellular locations are translated in these locations. By revealing the existence of correlations of spatial distributions for mRNA-protein pairs at different time points, which are associated with the MTOC orientation and can be impaired by applying perturbations including inhibition of protein translation, our results suggest local translation. Additionally, our methodology can be applied to study variability of localized gene expression in morphologically constrained biological contexts.

We revealed various aspects of the spatial distribution of specific mRNA transcripts and proteins of interest in time, such as *Arhgdia* and its protein product RhoGDIa. We have shown that the majority of the *Arhgdia* mRNA population relates to the MTOC position, with a small fraction being localized to the periphery. The reduced cytoplasmic MPI of *Arhgdia* mRNA, altered CS plots for Arhgdia mRNA-protein in the presence of a cytoskeletal polymerization inhibitor, and the reduction in peripheral enrichment and clustering of Arhgdia protein when translation is inhibited suggest MTOC-dependent peripheral local translation. Furthermore, depletion of PRRC2C, an *Arhgdia* mRNA binding protein, led to changes in the subcellular localization of *Arhgdia* in relation to the MTOC, changes in translation of the corresponding protein, and further downstream effects related to RhoGDIα function as a regulator of Rho GTPases, including inhibition of cell migration. Taken together, these data exemplify the importance of characterizing the spatial distribution of mRNA quantitatively and how measurable changes in the subcellular distribution of the transcript are linked to RNA and protein function.

Our analyses also revealed important aspects of Pard3 spatial and temporal distribution. The transcript and protein show a correlated temporal degree of clustering and a reduction in peripheral enrichment and clustering of the Pard3 protein in the presence of a translational inhibitor. In contrast to *Arhgdia*, there is an increase in the MPI of *Pard3* mRNA upon disruption with nocodazole, indicating MTOC-MT influence on *Pard3* transport. Localization of the Pard3 protein in peripheral clusters at cell-cell adhesions was previously shown in fibroblasts when grown in culture (Schmoranzer et al., 2009), which is concordant with the peripheral enrichment of *Pard3* mRNA and protein in our system.

Resolving spatial distribution of RNA and protein in a quantitative manner across time points

We have shown our approach to be suitable for the identification of patterns of RNA spatial and temporal distribution and RNA-protein interdependent localization, both globally and locally. We demonstrated how our analytical tools can be applied to micropatterned cells for studying RNA subcellular distribution and how it can be used for other constrained cellular geometries.

Several aspects of our approach are suitable as a basis for diverse kinds of spatially resolved omics (Crosetto et al., 2015).

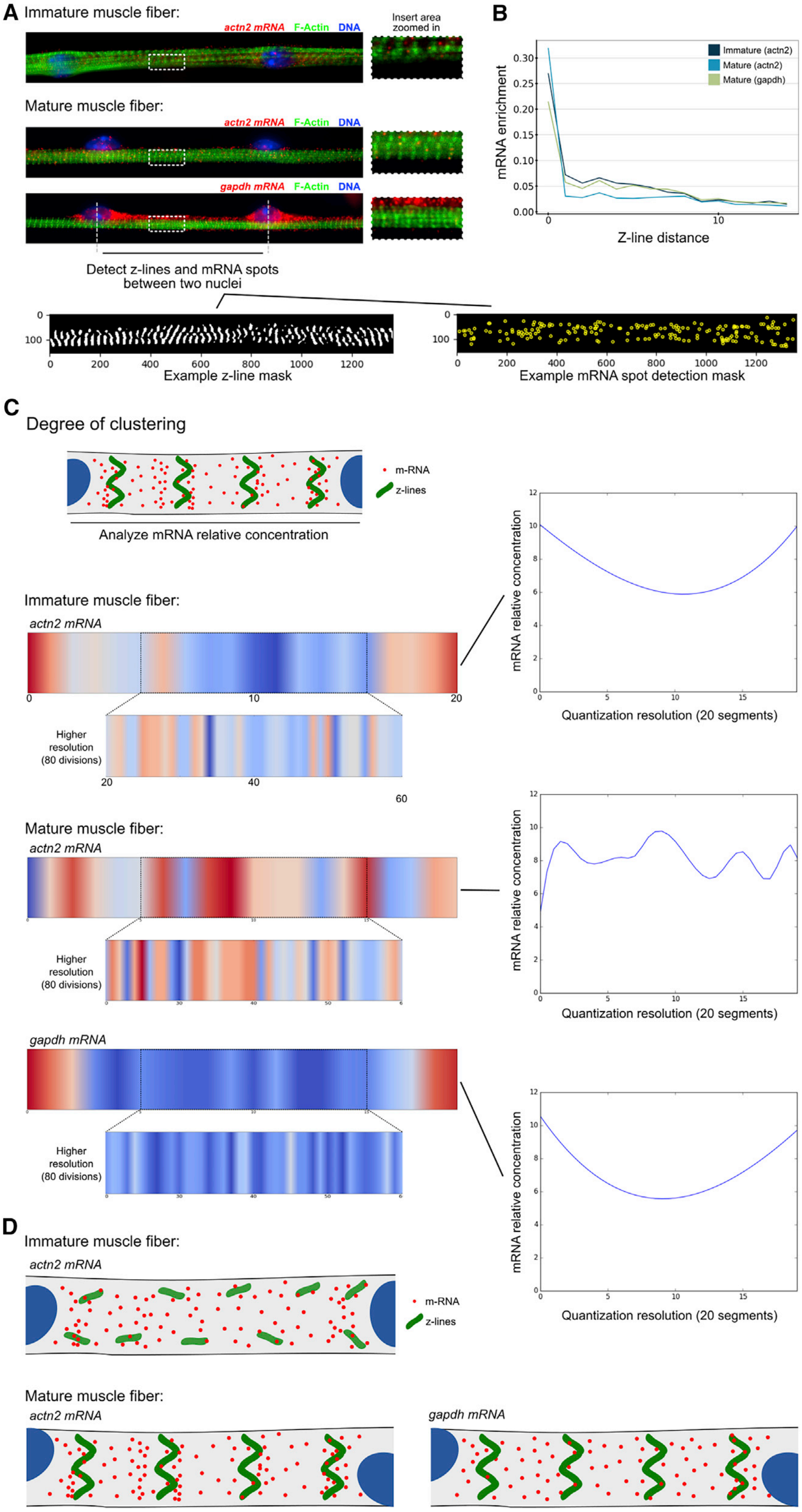

Figure 7. Sarcomeric mRNAs cluster in a striated pattern in differentiated myofibers

(A) Typical epifluorescent images of immature and mature muscle fibers. The DNA was stained with DAPI (blue), F-actin was visualized by using IF (green), and *Actn2/Gapdh* were visualized by using smFISH (red). Z-line and RNA spot detection masks were extracted by using IF and single-molecule data, respectively.

(B) mRNA distance profiles. For each mRNA we computed its distance to the closest Z lines, which allowed us to count the number of mRNAs having a certain distance to Z lines. Normalized median counts are represented on the y axis. A higher number of *actn2* immature mRNA falls inside or close to Z lines in comparison with mature fibers, suggesting greater clustering of mRNA between Z lines for mature *actn2*. The Z-line distance is a Euclidean distance.

(C) The mRNA local density was computed between two nuclei. Each cell was quantized in vertical quadrants, and relative concentration of mRNA in each quadrant was computed by normalizing the counts by the relevant surface. A wave-like clustering is observed for *actn2* in mature compared with immature fibers. No clustering is observed for *Gapdh*.

(D) Model describing *actn2* mRNA distribution in immature and mature fibers.

The quantitative nature of the computational analyses, autonomous image acquisition, and automated features of our data processing make DypFISH highly scalable to high-throughput studies and in mammalian tissue cells. Particularly, the capacity of the approach to investigate changes in localization under perturbation make DypFISH suitable for inferring spatially organized regulatory networks (Crosetto et al., 2015) and capable of being combined with multiplexing techniques (Chen et al., 2015) to reveal dynamic changes in cell state. As well as correlated mRNA-protein localization, our generalized clustering approach can be used to detect interdependent clustering patterns between RBPs and different kinds of RNAs (long noncoding RNAs, mRNAs, microRNAs) or interdependent protein-protein clustering patterns. Finally, we believe that techniques such as those presented here will help to make possible further development of integrated approaches and thus contribute to large-scale multi-omics studies at subcellular resolution, such as the Human Cell Atlas.

Limitations of study

This study provides a toolbox to characterize various aspects of subcellular localization of RNA and protein molecules. These include subcellular location, relation to a cellular marker, clustering patterns, and colocalization between molecules, among others. The limitations of this study include the following: (1) The subcellular localization of a biomolecule is defined in relation to a chosen cellular marker and it is not an absolute parameter. (2) Micropatterned cells and muscles have a specific, defined topology but standard cells in culture do not, and thus the definitions of "peripheral," "central," and so forth are less clear-cut. (3) The annotation of the MTOC has been done manually; automation is difficult, as the signal using the anti-tubulin antibody might be blurry, there is a certain percentage of cells with more than one MTOC, and the annotation often requires looking through a few Z slices. However, we do expect the annotation to be automated in the future. (4) Although many cell lines and some primary cells spread well on micropatterns, not all primary cell types and cell lines spread and adopt the shape of the micropattern, limiting the number of cell types that can be used. (5) The effect of modification of cell quantization parameters (such as number of quadrants and isolines) on the resulting statistical results has not been evaluated.

STAR★METHODS

Detailed methods are provided in the online version of this paper and include the following:

- KEY RESOURCES TABLE
- RESOURCE AVAILABILITY
  - Lead contact
  - Materials availability
- EXPERIMENTAL MODEL AND SUBJECT DETAILS
  - Cell culture and medium
- METHOD DETAILS
  - Cell culture and treatments
  - RNA isolation and analysis
  - Cell micropatterning
  - RNA probes and reagents
  - Immuno-RNA FISH staining
  - Immunofluorescence staining
  - Polarity and migration assays
  - Image acquisition
  - Materials
- QUANTIFICATION AND STATISTICAL ANALYSIS
  - Primary image descriptors
  - Secondary image descriptors
  - Statistical analysis
  - Additional methods for muscle data analysis
  - Additional methods for CHX inhibition analysis

SUPPLEMENTAL INFORMATION

Supplemental information can be found online at https://doi.org/10.1016/j.crmeth.2021.100068.

ACKNOWLEDGMENTS

We thank all members of the Gene Expression and Biophysics Laboratory (Mhlanga Lab). We thank N. Crosetto, and members of the L. Pelkmans lab, members of the J.-C. Olivo-Marin lab, K. Schauer, and members of the B. Goud lab for comments on the manuscript. A.F.S. is a recipient of a postdoctoral fellowship from the Claude Leon Foundation, South Africa. This research has been supported by the following grants, all to M.M.M. V2YGE81 (to A.F.S.) and PG-V2KYPO7, TA 2011 011 from the Council for Industrial and Scientific Research (CSIR) (South Africa) and by a grant from the Emerging Research Area Program of The Department of Science and Technology (South Africa) Department of Science & Technology Center of Competence Grant, SA Medical Research Council SHIP grant, and CSIR Parliamentary Grant. M.M.M. and M.N. are Chan Zuckerberg Investigators of the Chan Zuckerberg Initiative – Human Cell Atlas program.

AUTHOR CONTRIBUTIONS

Conceptualization, M.M.M., M.N., and A.F.S.; methodology, A.F.S., R.B., J.H.W., M.R.P., N.B., I.C.F., S.D., M.B., M.N., E.B., B.D., F.A., C.A.F., E.R.G., and J.-C.O.-M.; software, M.N., E.B., B.D., H.S., J.-C.O.-M., J.H.W., M.B., and S.D.; formal analysis, M.N., E.B., and B.D.; investigation, A.F.S., R.B., M.R.P., J.S., and I.C.F.; data curation, A.F.S., M.N., E.B., and B.D.; writing – original draft, A.F.S., R.B., J.H.W., M.N., M.R.P., and M.M.M.; writing – review and editing, M.N., M.M.M., and A.F.S.; supervision, M.M.M., C.A.F., E.R.G., and M.N.; project administration, M.M.M., M.N., and A.F.S.

DECLARATION OF INTERESTS

The authors declare no competing interests.

INCLUSION AND DIVERSITY

One or more of the authors of this paper self-identifies as an underrepresented ethnic minority in science. The author list of this paper includes contributors from the location where the research was conducted who participated in the data collection, design, analysis, and/or interpretation of the work.

Received: February 8, 2021
Revised: June 15, 2021
Accepted: August 3, 2021
Published: September 13, 2021

REFERENCES

Abràmoff, M.D., Magalhães, P.J., and Ram, S.J. (2004). Image processing with ImageJ. Biophoton. Int. *11*, 36–42.

Azioune, A., Storch, M., Bornens, M., Thery, M., and Piel, M. (2009). Simple and rapid process for single cell micro-patterning. Lab. Chip *9*, 1640–1642.

Bashirullah, A., Cooperstock, R.L., and Lipshitz, H.D. (1998). RNA localization in development. Annu. Rev. Biochem. *67*, 335–394.

Bassell, G.J., Zhang, H., Byrd, A.L., Femino, A.M., Singer, R.H., Taneja, K.L., Lifshitz, L.M., Herman, I.M., and Kosik, K.S. (1998). Sorting of beta-actin mRNA and protein to neurites and growth cones in culture. J. Neurosci. *18*, 251–265.

Batish, M., van den Bogaarda, P., Kramer, F.R., and Tyagi, S. (2012). Neuronal mRNAs travel singly into dendrites. Proc. Natl. Acad. Sci. U S A *109*, 4645–4650.

Battich, N., Stoeger, T., and Pelkmans, L. (2013). Image-based transcriptomics in thousands of single human cells at single-molecule resolution. Nat. Methods *10*, 1127–1133.

Battich, N., Stoeger, T., and Pelkmans, L. (2015). Control of transcript variability in single mammalian cells. Cell *163*, 1596–1610.

Besse, F., and Ephrussi, A. (2008). Translational control of localized mRNAs: restricting protein synthesis in space and time. Nat. Rev. Mol. Cell Biol. *9*, 971–980.

Bouvrette, L.P., Cody, N.A.L., Bergalet, J., Lefebvre, F.A., Diot, C., Wang, X., Blanchette, M., and Lécuyer, E. (2018). CeFra-seq reveals broad asymmetric mRNA and noncoding RNA distribution profiles in *Drosophila* and human cells. RNA *24*, 98–113.

Briley, W.E., Bondy, M.H., Randeria, P.S., Dupper, T.J., and Mirkin, C.A. (2015). Quantification and real-time tracking of RNA in live cells using Sticky-flares. Proc. Natl. Acad. Sci. U S A *112*, 9591–9595.

Bruusgaard, J.C., Liestøl, K., Ekmark, M., Kollstad, K., and Gundersen, K. (2003). Number and spatial distribution of nuclei in the muscle fibres of normal mice studied in vivo. J. Physiol. *551*, 467–478.

Bruusgaard, J.C., Liestøl, K., and Gundersen, K. (2006). Distribution of myonuclei and microtubules in live muscle fibers of young, middle-aged, and old mice. J. Appl. Physiol. *100*, 2024–2030.

Buxbaum, A.R., Wu, B., and Singer, R.H. (2014). Single β-actin mRNA detection in neurons reveals a mechanism for regulating its translatability. Science *343*, 419–422.

Cabili, M.N., Dunagin, M.C., McClanahan, P.D., Biasesch, A., Padovan-Merhar, O., Regev, A., and Raj, A. (2015). Localization and abundance analysis of human lncRNAs at single-cell and single-molecule resolution. Genome Biol. *16*, 20.

Carvalho, J.R., Fortunato, I.C., Franco, C.A., Fonseca, C.G., Pezzarossa, A., Barbacena, P., Dominguez-Cejudo, M.A., Vasconcelos, F.F., Santos, N.C., Carvalho, F.A., and Franco, C.A. (2019). Non-canonical Wnt signaling regulates junctional mechanocoupling during angiogenic collective cell migration. eLife *8*. https://doi.org/10.7554/eLife.45853.

Chen, K.H., Boettiger, A.N., Moffitt, J.R., Wang, S., and Zhuang, X. (2015). RNA imaging. Spatially resolved, highly multiplexed RNA profiling in single cells. Science *348*, aaa6090.

S.N. Chiu, D. Stoyan, W. Kendall, and J. Mecke, eds. (2013). Stochastic Geometry and its Applications, 3rd Edition (Wiley-Blackwell).

Chouaib, R., Safieddine, A., Pichon, X., Imbert, A., Kwon, O.S., Samacoits, A., Traboulsi, A.-M., Robert, M.-C., Tsanov, N., Coleno, A., Poser, I., et al. (2020). A dual protein-mRNA localization screen reveals compartmentalized translation and widespread co-translational RNA targeting. Dev. Cell *54*, 773–791.e5.

Clatterbuck-Soper, S.F., Jackrel, M.E., Shorter, J., and Mili, S. (2017). FUS inclusions disrupt RNA localization by sequestering kinesin-1 and inhibiting microtubule detyrosination. J. Cell Biol. *216*, 1015–1034.

Crosetto, N., Bienko, M., and van Oudenaarden, A. (2015). Spatially resolved transcriptomics and beyond. Nat. Rev. Genet. *16*, 57–66.

de Chaumont, F., Dallongeville, S., Chenouard, N., Hervé, N., Pop, S., Provoost, T., Vannary, M.-Y., Pankajakshan, P., Lecomte, T., Le Montagner, Y., Lagache, T., Dufour, A., and Olivo-Marin, J.-C. (2012). Icy: an open bioimage informatics platform for extended reproducible research. Nat. Methods *9*, 690–696.

Denes, L.T., Kelley, C.P., and Wang, E.T. (2021). Microtubule-based transport is essential to distribute RNA and nascent protein in skeletal muscle. bioRxiv. https://doi.org/10.1101/2021.02.26.433059.

Edelstein, A.D., Tsuchida, M.A., Amodaj, N., Pinkard, H., Vale, R.D., and Stuurman, N. (2014). Advanced methods of microscope control using μManager software. J. Biol. Methods *1*, e10.

Falcone, S., Roman, W., Hnia, K., Gache, V., Didier, N., Lainé, J., Auradé, F., Marty, I., Nishino, I., Charlet-Berguerand, N., Romero, N.B., et al. (2014). N-WASP is required for Amphiphysin-2/BIN1-dependent nuclear positioning and triad organization in skeletal muscle and is involved in the pathophysiology of centronuclear myopathy. EMBO Mol. Med. *6*, 1455–1475.

Fomina-Yadlin, D., Du, Z., and McGrew, J.T. (2014). Gene expression measurements normalized to cell number reveal large scale differences due to cell size changes, transcriptional amplification and transcriptional repression in CHO cells. J. Biotechnol. *189*, 58–69.

Franzini-Armstrong, C., and Peachey, L.D. (1981). Striated muscle-contractile and control mechanisms. J. Cell Biol. *91*, 166s–186s.

Garcia-Mata, R., Boulter, E., and Burridge, K. (2011). The 'invisible hand': regulation of RHO GTPases by RHOGDIs. Nat. Rev. Mol. Cell Biol. *12*, 493–504.

Gomes, E.R., Jani, S., and Gundersen, G.G. (2005). Nuclear movement regulated by Cdc42, MRCK, myosin, and actin flow establishes MTOC polarization in migrating cells. Cell *121*, 451–463.

Gupta, S., Marcel, N., Sarin, A., and Shivashankar, G.V. (2012). Role of actin dependent nuclear deformation in regulating early gene expression. PLoS One *7*, e53031.

Hale, C.M., Chen, W.C., Khatau, S.B., Daniels, B.R., Lee, J.S., and Wirtz, D. (2011). SMRT analysis of MTOC and nuclear positioning reveals the role of EB1 and LIC1 in single-cell polarization. J. Cell Sci. *124*, 4267–4285.

Hengst, U., Deglincerti, A., Kim, J.K., Jeon, N.L., and Jaffrey, S.R. (2009). Axonal elongation triggered by stimulus-induced local translation of a polarity complex protein. Nat. Cell Biol. *11*, 1024–1030.

Jain, N., Iyer, K.V., Kumar, A., and Shivashankar, G.V. (2013). Cell geometric constraints induce modular gene-expression patterns via redistribution of HDAC3 regulated by actomyosin contractility. Proc. Natl. Acad. Sci. U S A *110*, 11349–11354.

Jansen, R.P. (2001). mRNA localization: message on the move. Nat. Rev. Mol. Cell Biol. *2*, 247–256.

Katz, Z.B., Wells, A.L., Park, H.Y., Wu, B., Shenoy, S.M., and Singer, R.H. (2012). β-Actin mRNA compartmentalization enhances focal adhesion stability and directs cell migration. Genes Dev. *26*, 1885–1890.

Kempe, H., Schwabe, A., Crémazy, F., Verschure, P.J., and Bruggeman, F.J. (2015). The volumes and transcript counts of single cells reveal concentration homeostasis and capture biological noise. Mol. Biol. Cell *26*, 797–804.

Kim, D.-H., Cho, S., and Wirtz, D. (2014). Tight coupling between nucleus and cell migration through the perinuclear actin cap. J. Cell Sci. *127*, 2528–2541.

Kislauskis, E.H., Zhu, X., and Singer, R.H. (1997). beta-Actin messenger RNA localization and protein synthesis augment cell motility. J. Cell Biol. *136*, 1263–1270.

Kloc, M., Zearfoss, N.R., and Etkin, L.D. (2002). Mechanisms of subcellular mRNA localization. Cell *108*, 533–544.

Kozachenko, L.F., and Leonenko, N.N. (1987). Sample estimate of the entropy of a random vector. Problemy Peredachi Informatsii. Problems Inform. Transmission *23*, 9–16.

La Manno, G., Soldatov, R., Zeisel, A., Braun, E., Hochgerner, H., Petukhov, V., Lidschreiber, K., Kastriti, M.E., Lönnerberg, P., Furlan, A., Fan, J., Borm, L.E., et al. (2018). RNA velocity of single cells. Nature *560*, 494–498.

Langdon, E.M., Qiu, Y., Ghanbari Niaki, A., McLaughlin, G.A., Weidmann, C.A., Gerbich, T.M., Smith, J.A., Crutchley, J.M., Termini, C.M., Weeks, K.M., Myong, S., and Gladfelter, A.S. (2018). mRNA structure determines specificity of a polyQ-driven phase separation. Science *360*, 922–927.

Lécuyer, E., Yoshida, H., Parthasarathy, N., Alm, C., Babak, T., Cerovina, T., Hughes, T.R., Tomancak, P., and Krause, H.M. (2007). Global analysis of mRNA localization reveals a prominent role in organizing cellular architecture and function. Cell *131*, 174–187.

CellPress
OPEN ACCESS

Lee, C., Zhang, H., Baker, A.E., Occhipinti, P., Borsuk, M.E., and Gladfelter, A.S. (2013). Protein aggregation behavior regulates cyclin transcript localization and cell-cycle control. Dev. Cell *25*, 572–584.

Macdonald, P.M., and Struhl, G. (1988). cis-acting sequences responsible for anterior localization of bicoid mRNA in *Drosophila* embryos. Nature *336*, 595–598.

Kunde, S.A., Musante, L., Grimme, A., Fischer, U., Müller, E., Wanker, E.E., and Kalscheuer, V.M. (2011). The X-chromosome-linked intellectual disability protein PQBP1 is a component of neuronal RNA granules and regulates the appearance of stress granules. Hum. Mol. Genet. *20*, 4916–4931.

Mardakheh, F.K., Paul, A., Kümper, S., Sadok, A., Paterson, H., Mccarthy, A., Yuan, Y., and Marshall, C.J. (2015). Global analysis of mRNA, translation, and protein localization: local translation is a key regulator of cell protrusions. Dev. Cell *35*, 344–357.

Martin, K.C., and Ephrussi, A. (2009). mRNA localization: gene expression in the spatial dimension. Cell *136*, 719–730.

Medioni, C., and Besse, F. (2018). The secret life of RNA: lessons from emerging methodologies. Methods Mol. Biol. *1649*, 1–28.

Mili, S., Moissoglu, K., and Macara, I. (2008). Genome-wide screen reveals APC-associated RNAs enriched in cell protrusions. Nature *453*, 115–119.

Moissoglu, K., Yasuda, K., Wang, T., Chrisafis, G., Mili, S., et al. (2019). Translational regulation of protrusion-localized RNAs involves silencing and clustering after transport. eLife *8*. https://doi.org/10.7554/eLife.44752.

Moor, A.E., Golan, M., Massasa, E.E., Lemze, D., Weizman, T., Shenhav, R., Baydatch, S., Mizrahi, O., Winkler, R., Golani, O., Stern-Ginossar, N., and Itzkovitz, S. (2017). Global mRNA polarization regulates translation efficiency in the intestinal epithelium. Science *357*, 1299–1303.

Olivo-Marin, J.-C. (2002). Extraction of spots in biological images using multiscale products. Pattern Recogn. *35*, 1989–1996.

Padovan-Merhar, O., Gautham, P.N., Biaesch, A.G., Mayer, A., Scarfone, S., Foley, S.W., Wu, A.R.L., Churchman, S., Singh, A., and Raj, A. (2015). Single mammalian cells compensate for differences in cellular volume and DNA copy number through independent global transcriptional mechanisms. Mol. Cell *58*, 339–352.

Park, H.Y., Lim, H., Yoon, Y.J., Follenzi, A., Nwokafor, C., Lopez-Jones, M., Meng, X., and Singer, R.H. (2014). Visualization of dynamics of single endogenous mRNA labeled in live mouse. Science *343*, 422–424.

Park, H.Y., Trcek, T., Wells, A.L., Chao, J.A., and Singer, R.H. (2012). An unbiased analysis method to quantify mRNA localization reveals its correlation with cell motility. Cell Rep. *1*, 179–184.

Pimentel, M.R., Falcone, S., Cadot, B., and Gomes, E.R. (2017). In vitro differentiation of mature myofibers for live imaging. J. Vis. Exp. *119*, 55141. https://doi.org/10.3791/55141.

Raj, A., van den Boogard, P., Rifkin, S.A., van Oudenaarden, A., and Tyagi, S. (2008). Imaging individual mRNA molecules using multiple singly labeled probes. Nat. Methods *5*, 877–879.

Razafsky, D., Wirtz, D., and Hodzic, D. (2014). Nuclear envelope in nuclear positioning and cell migration. Adv. Exp. Med. Biol. *773*, 471–490.

Ridley, A.J., Schwartz, M.A., Burridge, K., Firtel, R.A., Ginsberg, M.H., Borisy, G., Parsons, J.T., Horwitz, A.R., et al. (2003). Cell migration: integrating signals from front to back. Science *302*, 1704–1709.

Ripley, B.D. (1977). Modelling spatial patterns. J. R. Stat. Soc. *39*, 172–192.

Roman, W., Martins, J.P., Carvalho, F.A., Voituriez, R., Abella, J.V., Santos, N.C., Cadot, B., Way, M., and Gomes, E.R. (2017). Myofibril contraction and crosslinking drive nuclear movement to the periphery of skeletal muscle. Nat. Cel. Biol. *19*, 1189–1201.

Samacoits, A., Chouaib, R., Safieddine, A., Traboulsi, A.-M., Ouyang, W., Zimmer, C., Peter, M., Bertrand, E., Walter, T., and Mueller, F. (2018). A computational framework to study sub-cellular RNA localization. Nat. Commun. *9*, 4584.

Savulescu, A.F., Stoychev, S., Mamputha, S., and Mhlanga, M.M. (2020). Biochemical pulldown of mRNAs and long noncoding RNAs from cellular lysates coupled with mass spectrometry to identify protein binding partners. Bio Protoc. *10*, e3639.

Schauer, K., Duong, T., Bleakley, K., Bardin, S., Bornens, M., and Goud, B. (2010). Probabilistic density maps to study global endomembrane organization. Nat. Methods *7*, 560–566.

Schmoranzer, J., Fawcett, J.P., Segura, M., Tan, S., Vallee, R.B., Pawson, T., and Gundersen, G.G. (2009). Par3 and dynein associate to regulate local microtubule dynamics and centrosome orientation during migration. Curr. Biol. *19*, 1065–1074.

Sharp, J.A., Plant, J.J., Ohsumi, T.k., Borowsky, M., and Blower, M.D. (2011). Functional analysis of the microtubule-interacting transcriptome. Mol. Biol. Cell *22*, 4312–4323.

Strell, C., Hilsche, M.M., Laxman, N., Svedlund, J., Wu, C., Yokota, C., and Nilsson, M. (2019). Placing RNA in context and space—methods for spatially resolved transcriptomics. FEBS J. *286*, 1468–1481.

Stueland, M., Wang, T., Park, H.Y., and Mili, S. (2019). RDI calculator: an analysis tool to assess RNA distributions in cells. Sci. Rep. *9*, 8267.

Tautz, D., and Pfeifle, C. (1989). A non-radioactive in situ hybridization method for the localization of specific RNAs in *Drosophila* embryos reveals translational control of the segmentation gene hunchback. Chromosoma *98*, 81–85.

Théry, M. (2010). Micropatterning as a tool to decipher cell morphogenesis and functions. J. Cell Sci. *123*, 4201–4213.

Théry, M., Racine, V., Pepin, A., Piel, M., Chen, Y., Jean-Baptiste, S., and Bornens, M. (2005). The extracellular matrix guides the orientation of the cell division axis. Nat. Cel. Biol. *7*, 947–953.

Théry, M., Racine, V., Piel, M., Pepin, A., Dimitrov, A., Chen, Y., Jean-Baptiste, S., and Bornens, M. (2006). Anisotropy of cell adhesive microenvironment governs cell internal organization and orientation of polarity. Proc. Natl. Acad. Sci. U S A *103*, 19771–19776.

Vargha, A., and Delaney, H.D. (2000). A critique and improvement of the CL common language effect size statistics of McGraw and Wong. J. Educ. Behav. Stat. *25*, 101–132.

Vilmont, V., Cadot, B., Ouanounou, G., and Gomes, E.R. (2016). A system for studying mechanisms of neuromuscular junction development and maintenance. Development *143*, 2464–2477.

Warrell, J.H., Savulescu, A.F., Brackin, R., and Mhlanga, M.M. (2016). Generalized statistical tests for mRNA and protein subcellular spatial patterning against complete spatial randomness. arXiv. http://arxiv.org/abs/1602.06429.

Weis, B.L., Schleiff, E., and Zerges, W. (2013). Protein targeting to subcellular organelles via mRNA localization. Biochim. Biophys. Acta Mol. Cell Res. *1833*, 260–273.

Xie, F., Shao, S., Aziz, A.U.R., Zhang, B., Wang, H., and Liu, B. (2017). Role of Rho-specific guanine nucleotide dissociation inhibitor α regulation in cell migration. Acta Histochem *119* (*3*), 183–189. https://doi.org/10.1016/j.acthis.2017.01.008.

Yamagishi, M., Shirasaki, Y., and Funatsu, T. (2009). Size-dependent accumulation of mRNA at the leading edge of chicken embryo fibroblasts. Biochem. Biophys. Res. Commun. *390*, 750–754.

Yoon, Y.J., Wu, B., Buxbaum, A.R., Das, S., Tsai, A., English, B.P., Grimm, J.B., Lavis, L.D., and Singer, R.H. (2016). Glutamate-induced RNA localization and translation in neurons. Proc. Natl. Acad. Sci. U S A *113*, E6877–E6886.

Zappulo, A., van den Bruck, D., Ciolli Mattioli, C., Franke, V., Imami, K., McShane, E., Moreno-Estelles, M., Calviello, L., Filipchyk, A., Peguero-Sanchez, E., Müller, T., Woehler, A., et al. (2017). RNA localization is a key determinant of neurite-enriched proteome. Nat. Commun. *8*, 583. https://doi.org/10.1038/s41467-017-00690-6.

STAR★METHODS

KEY RESOURCES TABLE

REAGENT or RESOURCE	SOURCE	IDENTIFIER
Antibodies		
Anti-Tubulin	Abcam	# ab6160; RRID:AB_305328
anti RhoGDI antibody	Santa Cruz	# sc-360; RRID:AB_2227516
Anti-PARD3	Abcam	# ab64646; RRID:AB_1603911
Anti β-Actin	Santa Cruz	# sc-81178; RRID:AB_2223230
Anti Gapdh	Santa Cruz	# sc-25778; RRID:AB_10167668
anti-GM130	BD Biosciences	# 610823; RRID:AB_398142
Goat Anti-RABBIT IgG ATTO 550 Conjugated	Rockland	# 611-154-122S; RRID:AB_10894121
Donkey anti Rat AlexaFluor647	Abcam	# ab150151
Donkey anti Mouse Alexa488	Thermo Fisher Scientific	# A21202; RRID:AB_141607
Chemicals, peptides, and recombinant proteins		
Collagenase type I	Sigma-Aldrich	Cat #C0130
Dispase	Roche	Cat #04942078001
Iscove's Modified Dulbecco's Medium (IMDM) with GlutaMAX	Invitrogen	Cat #31980022
Fetal bovine serum (FBS)	Eurobio	Cat #CVFSVF00-01
Penicillin/streptomycin (Penstrep)	Thermo Fisher Scientific	Cat #15140122
Matrigel	Corning	Cat #354230
Recombinant Rat Agrin	R&D systems	Cat #550-AG-100
Horse serum	Thermo Fisher Scientific	Cat #26050088
DMEM/F12 medium	Thermo Fisher scientific (Gibco)	# 11054001
EGM-2 Bulletkit	Lonza	# CC-3162
FBS	Biochrom	# S0613
Cytochalasin D	Sigma	# 22144-77-0
Cycloheximide	Sigma	# 66-81-9
Nocodazole	Sigma	# 31430-18-9
penicillin/streptomycin	Gibco	# 15140122
Hepes	Sigma	# H0887-100 ML
Human umbilical vein endothelial cells (HUVECs)	Lonza	# C2519A
PLL(20)-g[3.5]- PEG(2)	Surface Solutions (SuSoS)	N/A
PBS	Thermo Fisher Scientific (Gibco)	# 10010023
Fibronectin	Thermo Fisher Scientific (Gibco)	# 3010018
Fibrinogen-Alexa Fluor 488	Thermo Fischer Scientiifc (Invitrogen)	# F13191
$NaHCO_3$	Sigma	# 144-55-8
Sodium tetraborate	Sigma	# 1330-43-4
ATTO-565 NHS-ester dye	ATTO-TEC	# AD 565
Formaldehyde	Sigma	# 50-00-0
SSC-Buffer	Sigma	# S6639-1L
Ethanol 99.8 %	Sigma	# 64-17-5
Dextran Sulfate	VWR	# 0198-50G
Ribonucleoside Vanadyl Complex	NEB (New England Labs)	# S1402S

(Continued on next page)

Continued

REAGENT or RESOURCE	SOURCE	IDENTIFIER
tRNA from E. coli MRE 600	Sigma	# TRNAMRE-RO
Formamide	Sigma	# F9037-100ML
BSA	Thermo Fisher Scientific	# AM2616
D-(+)-Glucose solution	Sigma	# 50-99-7
Glucose oxidase	Sigma	# 9001-37-0
Catalase	Sigma	# 9001-05-2
DAPI	Life Technologies	# D1306
Critical commercial assays		
DharmaFECT one reagent	Dharmacon, GE Healthcare	# T-2005-01
RNeasy Mini Kit (Qiagen)	Qiagen	# 74104
Superscript IV First-Strand Synthesis System	Invitrogen	# 8091050
Experimental models: Cell lines		
NIH/3T3	ATCC, Cellonex	# CCL-92
Human umbilical vein endothelial cells (HUVECs)	Lonza	# C2519A
Experimental models: Organisms/strains		
Mouse: C57BL/6 Strain	Charles River	code: 027
Oligonucleotides		
ON-TARGETplus Human PRRC2C (23215) siRNA - SMARTpool, 10 nmol	Dharmacon	# L-014078-00-0010
On-Target*plus* Non-targeting siRNA #1, 20 nM	Dharmacon	# D-001810-01-05
human GAPDH forward sequence GTCAAGGCTGAGAACGGGAA	This manuscript	N/A
human GAPDH reverse sequence TGGACTCCACGACGTACTCA	This manuscript	N/A
human PRRC2C forward sequence GAAGCAGTTCCAGTCAGCC	This manuscript	N/A
human PRRC2C reverse sequence GTTGCGTGGACTGAAGAACC	This manuscript	N/A
Gapdh probes - 24-48 20-mers, each 20-mer containing a mdC(TEG-Amino) 3' modification used to conjugate an NHS-ester ATTO-565 fluorescent dye (ATTO-TEC) to the probe	Biosearch Technologies	N/A
Arhgdia probes - 24-48 20-mers, each 20-mer containing a mdC(TEG-Amino) 3' modification used to conjugate an NHS-ester ATTO-565 fluorescent dye (ATTO-TEC) to the probe	Biosearch Technologies	N/A
β-Actin probes - 24-48 20-mers, each 20-mer containing a mdC(TEG-Amino) 3' modification used to conjugate an NHS-ester ATTO-565 fluorescent dye (ATTO-TEC) to the probe	Biosearch Technologies	N/A
Rab13 probes - 24-48 20-mers, each 20-mer containing a mdC(TEG-Amino) 3' modification used to conjugate an NHS-ester ATTO-565 fluorescent dye (ATTO-TEC) to the probe	Biosearch Technologies	N/A

(*Continued on next page*)

Continued		
REAGENT or RESOURCE	SOURCE	IDENTIFIER
Pkp4 probes - 24-48 20-mers, each 20-mer containing a mdC(TEG-Amino) 3' modification used to conjugate an NHS-ester ATTO-565 fluorescent dye (ATTO-TEC) to the probe	Biosearch Technologies	N/A
Pard3 probes - 24-48 20-mers, each 20-mer containing a mdC(TEG-Amino) 3' modification used to conjugate an NHS-ester ATTO-565 fluorescent dye (ATTO-TEC) to the probe	Biosearch Technologies	N/A
Deposited data		
HDF5 data files of primary and secondary image descriptors for FISH Micropatterned images, CHX, CytoD, Nocodazole, PRRC2C and Muscle data	http://dypfish.org/	https://doi.org/10.5281/zenodo.5155127
Software and algorithms		
Chemotaxis and Migration Tool	Chemotaxis and Migration Tool (https://ibidi.com/chemotaxis-analysis/171-chemotaxis-and-migration-tool.html)	N/A
FiJi/ImageJ (2.0.0)	Schindelin et al., 2012	N/A
GraphPad Prism	GraphPad Prism (https://graphpad.com)	N/A
MATLAB - polarity analysis script to calculate the Polarity Index (PI)	Carvalho et al., 2019	N/A
ICY and μManager	de Chaumont et al., 2012 https://icy.bioimageanalysis.org Edelstein et al., 2014 https://doi.org/10.14440/jbm.2014.36	N/A
SV Control software	Optical Biosystems	N/A
HDF5	The HDF Group, Hierarchical data format version 5, 2000-2010, http://www.hdfgroup.org/HDF5	N/A
DypFISH code	https://github.com/cbib/dypfish	https://doi.org/10.5281/zenodo.5153514

RESOURCE AVAILABILITY

Lead contact

Further information and requests for resources should be directed to and will be fulfilled by the lead contact, Musa M. Mhlanga (belenus@mhlangalalab.org).

Materials availability

This study did not generate new unique reagents.

EXPERIMENTAL MODEL AND SUBJECT DETAILS

Cell culture and medium

NIH/3T3 cells (mouse fibroblast sarcoma cell line) were purchased from ATCC, Cellonex and were cultured in DMEM-F12 (Thermo Fisher Scientific - Gibco, # 11054001) supplemented with 10 % fetal bovine serum (Biochrom, # S0613). Human umbilical vein endothelial cells (HUVECs) were purchased from Lonza, and cultured in EGM-2 Bulletkit (Lonza, # CC-316) supplemented with 1% penicillin/streptomycin (Gibco, # 15140122). Primary muscle cells were isolated from newborn mice and differentiated as described in Pimentel et al., 2017.

METHOD DETAILS

Cell culture and treatments

NIH/3T3 cells were grown at 37°C to 100 % confluence in DMEM/F12 medium supplemented with 10 % FBS in a humidified atmosphere containing 5 % CO_2. Prior to micropatterning cells were serum-starved for 16 hr in DMEM/F12. For disruption of microtubule

polymerization, nocodazole (Sigma) was added to a final concentration of 50 ng/ml to the medium, post removal of unattached cells and incubated for 3/5 hours before fixation of cells. Cytochalasin D (Sigma) was added to a final concentration of 1 mg/ml to the medium post removal of unattached cells and incubated for 1 h before fixation of cells. Cycloheximide (Sigma) was added to a final concentration of 10 μg/ml 24 h prior to seeding on micropatterns, as well as post washes of unattached cells, following standard practice. HUVECs were cultured following the manufacturer's guidelines with complete medium EGM-2 Bulletkit (CC-3162, Lonza) supplemented with 1% penicillin/streptomycin (#15140122, Gibco). HUVECs were transfected with 25 nM of siRNA using the DharmaFECT one reagent (Dharmacon, GE Healthcare) and following the Dharmacon siRNA Transfection Protocol. L-014078-00-0010 ON-TARGETplus Human PRRC2C (23215) siRNA - SMARTpool, 10 nmol and D-001810-01-05 On-Target*plus* Non-targeting siRNA #1 (Dharmacon), 20 nM.

RNA isolation and analysis

RNA extraction from HUVECs was performed using RNeasy Mini Kit (Qiagen) as described by the manufacturer's protocol, followed by cDNA synthesis (Superscript IV First-Strand Synthesis System, Invitrogen) and subsequent quantitative real-time PCR (RT-qPCR). The following primers were used: human GAPDH forward sequence GTCAAGGCTGAGAACGGGAA and reverse sequence TGGACTCCACGACGTACTCA, human PRRC2C forward sequence GAAGCAGTTCCAGTCAGCC and reverse sequence GTTGCGTGGACTGAAGAACC, mouse PRRC2C forward sequence GAAGCAGTTCCAGTCAGCC and reverse sequence GTTGCGTGGACTGAAGAACC.

Cell micropatterning

Micropattern production was performed as previously described (Azioune et al., 2009). Briefly, glass coverslips were exposed to deep UV light using a UVO Cleaner (Jelight Company) for 5 mins. Cleaned coverslips were incubated with 0.1 mg/ml PLL-g-PEG (Surface Solutions) in 10 mM HEPES, pH 7.4 at RT for 1 hr. They were then rinsed once in PBS followed by one rinse in MilliQ water. The pegylated glass coverslips were then placed on a custom designed chromium photomask (Delta mask) (containing the desired micropatterns) and exposed to deep UV light for 5 mins. The patterned glass coverslips were then incubated with a fibronectin/fibrinogen-Alexa Fluor488 mixture (Life Technologies) in 100 mM $NaHCO_3$, pH 8.5, at RT for 1 hr. The coverslips were then rinsed in PBS and used immediately for cell seeding. Serum-starved NIH/3T3 cells were seeded on the micropatterned surfaces at a density of 10,000 cells/cm^2. After 30 mins, unattached cells were removed by gentle aspiration and replacement of the medium to DMEM/F12 medium supplemented with 10% FBS, which induced polarization of cells. Attached micropatterned cells were incubated at 37°C for 2 to 7 hours.

RNA probes and reagents

Design and manufacture of RNA FISH probes for use in the single molecule FISH method were performed according to the protocol by (Raj et al., 2008). Multiple 20-mer oligonucleotide probes targeting the following mRNAs: *Gapdh*, *Arhgdia*, *β-Actin*, *Rab13*, *Pkp4* and *Pard3* were purchased (Biosearch Technologies). Each 20-mer contains a mdC(TEG-Amino) 3' modification used to conjugate an NHS-ester ATTO-565 fluorescent dye (ATTO-TEC) to the probe. In brief, concentrated oligonucleotide probes were resuspended in 0.1 M Sodium tetraborate (Sigma) and mixed with resuspended 0.25 mg of the NHS-ester dye and incubated overnight at 37°C. This was followed by ethanol precipitation of the probes and purification by reverse phase HPLC on a XBRIDGETM OST C18 column to enrich for dye conjugated probes.

Immuno-RNA FISH staining

For experiments utilizing the *Gapdh*, *Arhgdia*, *β-Actin* RNA probes: micropatterned NIH/3T3 cells were fixed in 3.7 % formaldehyde for 10 min at 37°C followed by washes in PBS and overnight permeabilization in 70% ethanol at 4°C. For experiments utilizing the *Rab13*, *Pkp4*, *Pard3* RNA probes: micropatterned NIH/3T3 cells were fixed in pre-chilled methanol for 10 min, followed immediately by RNA FISH. The single molecule FISH method was modified from (Raj et al., 2008) to include immunofluorescence staining to detect the microtubule cytoskeleton. Cells were rehydrated in wash buffer (10 % formaldehyde, 2X SSC) for 5 min. Hybridization was conducted overnight in a humidified chamber at 37°C in Hyb buffer (10 % dextran sulfate, 1 μg/μl E.coli tRNA, 2mM Vanadyl ribonucleoside complex, 0.02 % RNAse-free BSA, 10 % formamide, 2X SSC) combined with 50 ng of the desired RNA probe along with primary antibody - rat monoclonal anti-tubulin antibody (Abcam). Cells were then washed 2X (30 min at room temperature) with antibody wash buffer (10 % formaldehyde, 2X SSC, anti-rat secondary antibody conjugated to Alexa Fluor 647 (Abcam)) followed by 1X wash with wash buffer. Cells were then incubated in equilibration buffer (0.4 % glucose, 2X SSC) for 5 mins and counter stained with 1 μg/ml DAPI (4',6-diamidino-2-phenylindole; Life Technologies). Coverslips were mounted in imaging buffer (3.7 μg/μl glucose oxidase and 1U catalase in equilibration buffer) and imaged.

Immunofluorescence staining

Micropatterned cells were fixed in 3.7 % formaldehyde for 10 min at 37°C, then washed with PBS followed by overnight incubation in 70 % ethanol at 4°C. The cells were then washed with FBS followed by permeabilization for 10 min in 0.25 % Triton-X at room temperature. Following this, the cells were washed thrice with PBS for 5 min each and incubated in blocking buffer (0.2 % BSA/PBS) for 30 min at room temperature. The cells were then incubated in the desired primary antibody solution (diluted in PBS) along with rat

monoclonal anti-tubulin antibody (Abcam) to detect the microtubule cytoskeleton for 1 hr at room temperature. RhoGDIα, Par3, β-Actin and Gapdh proteins were detected using rabbit polyclonal anti-Arhgdia (Santa Cruz), rabbit polyclonal anti-Pard3 (Abcam), mouse monoclonal β-Actin (Santa Cruz) and mouse monoclonal anti-Gapdh (Santa Cruz) respectively. Cells were then washed 3X with PBS following incubation with corresponding anti-rabbit secondary antibody conjugated to ATTO 550 (Rockland) together with anti-rat secondary antibody conjugated to Alexa Fluor 647 (Abcam) for 1 hr at room temperature. A further 3X wash with PBS was conducted followed by incubation in equilibration buffer (0.4 % glucose, 2X SSC) for 5 mins and counter stained with 1 μg/ml DAPI (4',6-diamidino-2-phenylindole; Life Technologies). Coverslips were mounted in imaging buffer (3.7 μg/μl glucose oxidase and 1U catalase in equilibration buffer) and imaged. For immunofluorescence in HUVECs, cells were fixed using 1% Paraformaldehyde (PFA) supplemented with 1 M $MgCl_2$ and 1 M $CaCl_2$ (1 μl/2 ml). Blocking and permealization was made with 3 % BSA in PBS-T (PBS with 0.1 % Triton X-100). Primary antibody - mouse anti-GM130 (BD Biosciences) was diluted in blocking solution and incubated for 2 h. Secondary antibody donkey anti-mouse Alexa488 (Thermo Fisher Scientific) was diluted in the blocking solution [3% BSA in PBS-T (PBS with 0.1 % Triton X-100)] and incubated for 1 h. For nucleus staining, HUVECs were incubated with 1x DAPI and then coverslips were mounted on microscopy glass slides using Mowiol DABCO (Sigma-Aldrich).

Polarity and migration assays

To calculate axial cell polarity and analyze cell migration, scratch-wound assays were performed. The wound was created on the surface of a 12-well-plate for cell migration assessment and on a microscopy glass-slide for axial polarity measurements with a 200 μL pipette tip. HUVECs migration was followed for 16 hours, individual cells were tracked and migration behavior, directness and cell velocity, were analyzed using the FIJI TrackMate plug in and the Chemotaxis and Migration Tool (free software from Ibidi). Tile-scan images of cells stained for Golgi (GM130) and nucleus (DAPI) were imported and analyzed in MATLAB using a polarity analysis script in order to calculate the Polarity Index (PI). PI shows the orientation strength of the cell monolayer and it varies from 0 to +1, where 0 corresponds to random localization of the Golgi and +1 corresponds to same directionality of the Golgi in all.

Image acquisition

Most samples were imaged on a custom built spinning disk confocal Revolution XD system (Andor) comprising of a Zeiss Axio Observer.Z1 microscope with a 63X Plan-Apochromat objective (numerical aperture 1.4) and a cooled EMCCD camera (Andor iXon 897). Z-dimension positioning and control was accomplished by a piezoelectric motor (NanoScanZ, Prior Scientific). Images were captured using a custom developed algorithm based on ICY and μManager that allowed autonomous image acquisition (Figure S1A). In brief, the position of the micropatterns on the micropatterned surface were determined autonomously using the grid detection, alignment and calibration algorithm. This was then followed by sequential autonomous stepping through the micropatterned grid to determine the presence of a cell on the micropattern. If a single cell was detected on the micropattern surface by the algorithm then a *z*-dimension series of images was captured every 0.3 μm in four different fluorescence channels using emission filters for DAPI (DNA), Alexa Fluor 488 (micropatterns), ATTO 565 (mRNA/protein) and Alexa Fluor 647 (tubulin) and exposure times of 10 ms, 350 ms, 1 s (mRNA) or 500 ms (protein) and 350 ms respectively. A few samples were imaged on a custom built Nikon Ti Eclipse widefield TIRF microscope using a 100X N.A. 1.49 Nikon Apochromat TIRF oil immersion objective and equivalent fluorescent channels as above, followed by processing using an automated background noise subtraction algorithm using ImageJ (Abràmoff et al., 2004). Samples were also imaged on a StellarVision microscope using Synthetic Aperture Optics technology (Optical Biosystems) and processed to obtain stacked single cell images for analysis. These are listed in the relevant supplementary tables. All images acquired on the Zeiss Axio Observer.Z1 microscope were 512x512 pixels in size, whereas the ones acquired on the StellarVision were 251x251 pixels. See Tables of image acquisition characteristics for image counts.

Materials

Images were acquired for *Arhgdia*, *Gapdh*, *β-Actin*, *Pard3*, *Pkp4*, and *Rab13* genes. Tables of image acquisition characteristics below recapitulate acquisition conditions, techniques and number of acquired images in each series of FISH and IF data.

The following table recapitulates all the image acquisition series characteristics and numbers for mouse fibroblast cells grown in micropatterned and standard cultures (indicated by *).

Gene	Molecular species	Technique	Time point (number of images)
Arhgdia*	mRNA	FISH	1 h (76); 3 h (24)
Arhgdia (control)	mRNA	FISH	2 h (59); 3 h (45); 4 h (52); 5 h (48)
Arhgdia (control)	protein	IF	2 h (31); 3 h (14); 5 h (61); 7 h (21)
β-Actin	mRNA	FISH	2 h (41); 3 h (42); 4 h (28); 5 h (29)
β-Actin	protein	IF	2 h (9); 3 h (19); 5 h (17); 7 h (24)
Gapdh	mRNA	FISH	2 h (58); 3 h (62); 4 h (50); 5 h (43)
Gapdh	protein	IF	2 h (27); 3 h (31); 5 h (21); 7 h (21)

(*Continued on next page*)

Continued

Gene	Molecular species	Technique	Time point (number of images)
Pard3 (control)	mRNA	FISH	2 h (25); 3 h (13); 4 h (15); 5 h (12)
Pard3 (control)	protein	IF	2 h (25); 3 h (20); 5 h (9); 7 h (26)
Pkp4	mRNA	FISH	2 h (17); 3 h (32); 4 h (11); 5 h (26)
Rab13	mRNA	FISH	2 h (16); 3 h (13); 4 h (22); 5 h (8)

Table describing Image acquisition characteristics for micropatterned and cultured cells.

Image acquisition series characteristics and numbers for mouse fibroblast cells grown in micropatterned cultures in control and drug-disrupted conditions for the CytoD experiment are detailed in the following table.

Gene (condition)	Molecular species	Technique	Time point (number of images)
Arhgdia (Control)	mRNA	FISH	1 h 15 (19)
Arhgdia (Control)	Protein	IF	1 h 15 (19)
Arhgdia (CytoD)	mRNA	FISH	1 h 15 (22)
Arhgdia (CytoD)	protein	IF	1 h 15 (25)
Pard3 (Control)	protein	IF	1 h 15 (24)
Pard3 (CytoD)	protein	IF	1 h 15 (20)

Table describing the image acquisition characteristics for the CytoD experiments.

Below are listed the image acquisition series characteristics and numbers for mouse fibroblast cells grown in micropatterned cultures in control and drug-disrupted conditions for the nocodazole experiment.

Gene (condition)	Molecular species	Technique	Time point (number of images)
Arhgdia (control)	mRNA	FISH	2 h (59); 3 h (45); 4 h (52); 5 h (50)
Arhgdia (control)	protein	IF	2 h (31); 3 h (14); 5 h (61); 7 h (21)
Arhgdia (nocodazole)	mRNA	FISH	3 h (41); 5 h (32)
Arhgdia (nocodazole)	protein	IF	3 h (20); 5 h (20)
Pard3 (control)	mRNA	FISH	2 h (29); 3 h (13); 4 h (15); 5 h (12)
Pard3 (control)	protein	IF	2 h (25); 3 h (20); 5 h (9); 7 h (26)
Pard3 (nocodazole)	mRNA	FISH	3 h (25); 5 h (21)
Pard3 (nocodazole)	protein	IF	3 h (14); 5 h (22)

Table describing the image acquisition characteristics for the Nocodazole experiments.

All image acquisition series characteristics and numbers for mouse fibroblast cells grown in micropatterned cultures in control and drug-disrupted conditions for the CHX experiment are presented in the following table.

Gene (condition)	Molecular species	Technique	Time point (number of images)
Arhgdia (Supp. control)	mRNA	FISH	2 h (19); 3 h (11); 5 h (11)
Arhgdia (Supp. control)	protein	IF	2 h (34); 3 h (25); 5 h (26)
Arhgdia (CHX)	mRNA	FISH	2h (5); 3h (2); 5h (4)
Arhgdia (CHX)	protein	IF	2h (16); 3h (16); 5h (16)
Pard3 (Supp. control)	mRNA	FISH	2h (15); 3h (12)
Pard3 (Supp. control)	protein	IF	2h (34); 3h (24); 5h (18)
Pard3 (CHX)	mRNA	FISH	2h (5); 3h (4); 5h (3)
Pard3 (CHX)	protein	IF	2h (10); 3h (12); 5h (3)

Table describing the image acquisition characteristics for the CHX experiments.

The following table recapitulates the image acquisition series characteristics and numbers for mouse fibroblast cells grown in micropatterned cultures in control and siRNA conditions for the PRRC2C experiment.

Gene	Molecular species	Technique	Time point (number of images)
Arhgdia (PRRC2C)	mRNA	FISH	PRRC2C (20); Control (38)
Arhgdia (PRRC2C)	protein	IF	PRRC2C (36); Control (46)

Table describing the image acquisition characteristics for the PRRC2C experiments.

Myofibers were differentiated and fixed as previously described (Pimentel et al., 2017; Roman et al., 2017).

Images were acquired for Actn2 and Gapdh genes. Acquisition conditions, techniques and number of acquired images in each series are recapitulated in the following table.

Gene	Element	Technique	Number of images
Actn2	Phalloidin mature fibers	FISH / IF	12
Actn2	Phalloidin immature fibers	FISH / IF	8
Gapdh	Phalloidin mature fibers	FISH / IF	14

Table describing the image acquisition characteristics for muscle cells.

In all of our experiments, we stained different cellular elements acquired at the same time as the FISH and IF signals, with different staining as detailed in the Feature staining table.

Feature	Staining
DNA	DAPI - 358⁄461
Micropatterns (only for micropatterned cells)	Fibrinogen - Alexa Fluor 488
Tubulin	Alexa Fluor 647

Table showing the summary of labeled cellular markers in the same images as the FISH and IF signals.

Images were acquired in the TIFF format. Our image processing pipeline transformed images into an HDF5 file (downloadable from the website www.dypfish.org).

QUANTIFICATION AND STATISTICAL ANALYSIS

All computational analysis performed in the DypFISH project and described below were implemented in Python.

Primary image descriptors

Given the TIFF files, we first computed primary image descriptors for each image and stored them in an HDF5 file for each acquisition series.

MTOC and nucleus centroid

FISH and IF images were manually annotated using the γ-Tubulin signal to obtain the coordinates (x, y) of the MTOC. The nucleus centroid was computed as the geometric center of the nucleus mask (see below).

Cell, nucleus and cytoplasm masks

Cell and nucleus masks were computed for all images (FISH and IF) using γ-Tubulin and DAPI signals, respectively.

For each image we obtained the maximum projection of the γ-Tubulin stained z-stack. A vignetting correction is further applied to each resulting image individually by performing a pixel wise multiplication between each pixel value and the vignetting function. The detected cells being in the microscope's focus, we assumed the optical center to be the center of the image and the intensity fall-off to be radially symmetric and the vignetting function is defined for each pixel x, $y$as $e^{-d \,/\, [(w/2)^2 * (h/2)^2]},$ where $d = (x - w/3)^2 + (y - h/2)^2$and $w$and $h$are the image's width and height, respectively. For contrast enhancement we performed histogram stretching by applying a linear normalization in order to stretch the interval of the intensities to the $[0, 255]$interval.

Cell Mask was detected from the γ-Tubulin channel. We applied a local entropy filter to each pixel i as follows: $e(i) = -\sum_{j \in N_i} p_j \, log_2 p_j$, where p_j is the proportion of pixels in the neighborhood N_i having the same intensity as pixel j. The neighborhood

size was set to30 × 30. For certain noisy image series we further applied a percentile thresholding to the resulting entropy histogram. As a last step, we performed Canny edge detection, which detected edges by applying Sobel operators to the smoothed image, followed by hysteresis. However, the resulting edges were usually non-contiguous due to a weak γ-Tubulin signal or a high rate of noise. Thus, we successively applied mathematical morphological operators, such as dilation and closing, followed by erosion conventionally used to fill small gaps.

As the result we obtained a contiguous contour to which we applied the marching squares algorithm in order to obtain a 2D cellular segmentation mask, $M^{cell}(x,y)$, which is 1 for the cellular region and 0 otherwise.

Nucleus mask procedure was very similar using the DAPI signal and yielding $M^{nucleus}(x,y)$, except that the local entropy filter was in most instances replaced by an Otsu filter, depending on the quality of the DAPI signal. Mathematical morphology algorithms were applied to neighborhoods ranging from 16×16 to 20×20 depending on the image acquisition characteristics.

Binary cellular and nucleus masks above were used to define a binary cytoplasm mask of the cell, $M^{cytoplasm}(x,y) = M^{cell}(x,y) \wedge \neg M^{nucleus}(x,y)$

Zero level

An acquired image stack might contain irrelevant slices because the focal field of the microscope is outside the cell (above or below). To determine which slice contained the bottom of the cell and had to be considered as the first relevant bottom slice of the stack, we defined the *zero level* descriptor corresponding to the index of the slice having the maximum summed γ-Tubulin intensity. This zero level reference *z*-slice was used in further analysis such as e.g. the height-map computation or the degree of clustering.

Height-map and cell volume

The height-map was built by segmenting each *z*-slice of a stacked image, which generated the 3D segmentation of the cell. It was performed for all FISH and IF images using the γ-Tubulin signal. Given a *z*-slice above the zero level we applied the cell mask detection procedure previously described, which defined a *z*-slice mask $M_z(x,y)$ with values corresponding to the height of the slice (z) within the mask and 0 outside. This set of slice masks defined the 3D representation, called height-map and denoted $h(x,y)$ where the value at each coordinate (x,y) is the maximum over all slice masks, $max_z(M_z(x,y))$.

Based on the height-map we defined the cell volume$V = \sum_{z=1}^{n} M_z(x,y)$ as the sum of volumes of all pixels within the height-map, where for each pixel $p \in M_z(x,y)$, its volume is$v(p) = (1 \div 9.75 \mu m)^2 \times 0.3 \mu m$, where 9.75 μm is a size coefficient between pixel in μm, and 0.3 $\mu m$is the height of the slice (Specific constants are dependent on the microscope and camera settings).

Protein intensities

Protein signal was computed for each immunofluorescence (IF) image as the sum of intensities across all z-slices and denoted as $I(x,y)$.

mRNA spot detection

To detect transcript positions from FISH data we used the ICY spot detector (Olivo-Marin, 2002). For images having $max(z) \leq 12$ we used the following parameters: 2D wavelets and sensitivity 70 at pixel-scale 2; otherwise the parameters were set to: 1 pixel and 2 pixel length-scales with sensitivity 80. These parameters have been adapted for the detection of transcripts in the PRRC2C analysis, the sensitivity has been set to 50 at pixel-scale 2 when the images have $max(z) \leq 12$; otherwise we used the 2D wavelets and a sensitivity of 70 at the pixel-scale 2.

For cultured cells, as well as for CytoD micropatterned cell series, we have applied a custom-developed spot detection script (these images present a very high noise content preventing efficient use of ICY). First, we applied a background noise subtraction by using Sobel and Gaussian filters, successively. Second, we applied the white top-hat filter in order to enhance bright objects of interest (potential mRNA spots) on a dark background. Finally, we used the Laplacian of Gaussians filter for mRNA spot detection. Furthermore, for the 3D analyses spots detected below the zero level were eliminated. Cells with less than 10 spots and 100 for the PRRC2C data (due to acquisition noise in cytoplasm) were eliminated.

Secondary image descriptors

Based on the primary image descriptors we computed secondary descriptors that corresponded to per image statistics.

Cytoplasmic total counts

Let us denote M the set of all mRNA spots for a given FISH image, $|M| = N$. The cytoplasmic total mRNA descriptor was calculated as the number of transcripts within $M^{cytoplasm}$, that is $T_{mRNA} = \left| \{ m \in M \mid M^{cytoplasm}(x,y) = 1 \} \right|$. The cytoplasmic total IF intensity is the summed IF intensity across the $M^{cytoplasm}$region for protein images: $T_{IF} = \sum \{ I(x,y) \mid M^{cytoplasm}(x,y) = 1 \}$.

Peripheral distance map

For a given image, the peripheral distance map corresponds to a collection of peripheral masks based on $M^{cytoplasm}(x,y)$, where the width of the periphery varies as a proportion of the cytoplasmic radial distance. We segmented $M^{cytoplasm}(x,y)$ into 100 isolines from the nucleus contour to the periphery by projecting a ray from the nucleus centroid to the cell border, which was then segmented in 100 equidistant points. The 100 isolines were then built by constructing polygons that connect 360 points (one ray per degree). These isolines define a symbolic distance map D, where $D(x,y)$ is the isobar value for (x,y) corresponding to the "distance" from the nucleus envelope, with 100 at the nucleus and 0 at the cell edge. Given a fixed percent p between 0 and 100, the mask $M^{periphery}(x,y,p)$ is 1 for $D(x,y)<p$ and 0 otherwise. Hence, the periphery mask for a given p contains a strip at the cell edge whose width is a fixed-proportion of the radial distance. Thus defined peripheral distance map allows to compute in 3D peripheral volumes $V^{periphery} = (V^i)$ at each isoline and consequently distances from any voxel to either cell periphery or nucleus wall in 3D.

Cell quantization

In order to compute localisation statistics over multiple micropatterned images, compatibility of these images is required. We have chosen the MTOC position to be the reference point for the 2D cell geometry.

2D quantization: quadrants and per quadrant statistics. As shown on the schematic in Figures 4A and 4B, given a cell mask $M^{cell}(x,y)$, we generate the tessellation of the image by centering two orthogonal axes at the nucleus centroid and rotating them over 360 degrees, each position of these axes defining a partition of the cell mask into four quadrants, one of them containing the MTOC, Q_M. We retained the orientation that maximizes the mRNA count within a quadrant containing the MTOC, that is $max_d(T_{mRNA} = \left|\{m \in M \mid M^{cytoplasm}(x,y) = 1 \wedge Q_M(x,y) = 1\}\right|)$, $d \in [0,359]$. The resulting four quadrants Q_1, Q_2, Q_3, Q_4 are numbered so that Q_1 always corresponds to $Q_M$and the remaining three quadrants are numbered in a clockwise fashion.

For protein intensities, quadrants are defined in a similar fashion using T_{IF}. Definition of cell mask partitioning in quadrants q, q_2, q_3, q_4 enables cell's quantization in 2D in terms of per quadrant statistics of mRNA and protein signal. Quadrants' respective areas are denoted by a_1, a_2, a_3, a_4. We denoted by t_i the total number of mRNA spots falling in q_i in the case of FISH data, or the summed intensity across q_i in the case of IF data.

Then the local mRNA density was computed as the relative concentration c_i of mRNA in quadrant i and is defined to be $c_i = \frac{t_i/a_i}{T_{mRNA}/A}$, where A is the cell mask area. In the case of protein signal we replaced T_{mRNA} by T_{IF}.

Fine-grained quantization. In the same fashion as for the peripheral distance map we defined an additional subdivision of the cellular mask in isolines, their number being defined by the percent p. Given the previously defined quadrants, we further subdivided each of them in 2, yielding the tessellation in 8 parts that divide the circle in 45 degree sectors. Using the isolines and the 8 sectors we quantized the cell masks into $8 \times p$ segments organized in a concentric fashion starting from the nucleus towards the cell periphery (see the schematic in Figure 5A). Quantization for thus obtained segments was computed in the same fashion as for quadrants, resulting in a $8 \times p$ vectors of per segment signal concentration statistics for each cell, that we denoted $C = (c_i)$.

3D quantization: 3D quadrants and per quadrant statistics. Cell mask's tessellation into quadrants as defined in II.3.A (axes position) is projected onto each z-slice, thus yielding the cell's partition into four 3D quadrants Q_1, Q_2, Q_3, Q_4, their respective volumes being denoted by v_1, v_2, v_3, v_4. The volume of each quadrant is calculated as the sum of volumes of pixels within it using the same coefficients as for the cell volume.

We denoted by t_i the total number of mRNA spots falling in Q_i in the case of FISH data, or the summed intensity across Q_i in the case of IF data.

Then the relative concentration c_i of mRNA in quadrant Q_i is defined to be$c_i = \frac{t_i/v_i}{T_{mRNA}/V}$. In the case of protein signal $T_{mRNA}$is replaced by T_{IF}.

In the same fashion as for 2D, we computed fine-grained quantization statistics in 3D. Specifically, signal densities (mRNA or protein) were computed for 8 quadrants and each $^1/_3$ of isolines, that is for 8×3 volumic segments, yielding signal concentration per volumic region vectors for each cell, that we denoted $C = (c_i)$. Notice that these vectors can be aligned between cells since all the quadrants are anchored by the MTOC position.

Statistical analysis

Primary and secondary image descriptors were used to compute statistics for image acquisition series and to compare them.

Peripheral fraction and enrichment

Based on the cytoplasm masks, we calculated the peripheral fraction of mRNA and proteins at a given percent p of the radial distance between the cell periphery and the cytoplasm. First, the peripheral density is defined as the ratio of the transcript counts (respectively, summed IF intensities) across the $M^{periphery}(x,y,p)$ and volumes of $M^{cytoplasm}$regions. First thanks to the distance map D we define a vector $C = (c_1, \ldots, c_{100})$ containing the counts of mRNAs (resp., summed intensities) at distances smaller or equal to $i \in [0, \ldots, 100]$ from the cell edge. This count vector C is divided by values of corresponding peripheral volumes $V^{periphery}$to obtain spot (or protein) densities in concentric regions from the periphery to each isoline (c_i/V^i). These local peripheral densities are further normalized by the total density of the cytoplasm $d_{cytoplasm} = N/V_{cytoplasm}$, resulting in a vector of relative densities for all regions from the periphery $F = \left(\frac{c_i/V^i}{d_{cytoplasm}}\right)$. The mRNA (resp., protein) fractions for each gene and for each isobar were defined as vector of median relative densities over all FISH images for this gene over all time points.

Volume corrected noise measure

We have measured the cell-to-cell expression variability in mRNA levels that cannot be accounted for by cell-to-cell differences in volume by computing the volume corrected noise measure Nm for micropatterned and standardly cultured cells. It was calculated following the approach of a previous study (Padovan-Merhar et al., 2015):

$$Nm = \left(\frac{\sigma_N}{E(N)}\right)^2 - \left(\frac{b \times E(V)}{a + b \times E(V)}\right)\left(\frac{Cov(m,V)}{E(m)\,E(V)}\right)$$

where N is the total mRNA count,$V$is the cell volume, a, b are the offset and slope of the least-squares best-fit linear regression of $E(N)$ on V, and σ, E and Cov are the notations for standard deviation, expectation and covariance, respectively.

Cytoplasmic spread

Cytoplasmic spread is measured by two statistical parameters that estimate how evenly a molecule is spread across the cell: cytoplasmic centrality and cytoplasmic entropy. The *centrality* statistics for mRNAs corresponds to the average distance from the nucleus envelope of cytoplasmic mRNAs, normalised by the maximal distance from the nucleus (corresponding to the number of isolines used to compute the distance map D). For the protein intensities, computation is the same but for peaks of intensity that are above the average. The higher the value, the closer to the periphery of the cell is the signal (mRNA or protein) distribution, value of 1 corresponding to 100% of the signal being at the periphery. The uniformity of the spread of molecule distributions in the cytoplasm is measured by computing Kozachenko-Leonenko entropy estimates H of a spatial random variable in 2D or in 3D based on the kth-nearest neighbour distances between mRNA (intensity peaks, respectively) coordinates following [Kozachenko and Leonenko, 1987]. Entropy is a unitless measure, to make the results comparable all the values are normalised by the $max(H)$across all cells.

MTOC polarity index

We defined a polarity index $PI_M \in [-1,1]$, termed the *MTOC polarity index*, that measures the enrichment of mRNA or protein signal for a given image acquisition series in the vicinity of the MTOC location.

For the set S of images from an acquisition series under study, we denoted by $S_M = \{S_i\}$ and $S_{\neg M} = \{S_j\}$ the sets of all MTOC containing quadrants and quadrants that do not contain the MTOC, respectively. Intuitively, the MTOC polarity index measures how frequently the relative concentration within the MTOC quadrants is higher than in the non-MTOC quadrants. Formally it is defined as follows:

$$PI_M = \frac{2\,|\{\, S_i \mid c_i > m \,\}|}{|\, S \,|} - 1,$$

where m is the median of relative signal concentrations c_j for quadrants in $S_{\neg M}$.

Positive values of PI_M imply MTOC correlated enrichment of RNA transcripts or proteins, negative values imply enrichment away from the MTOC and a value of zero implies no detectable enrichment.

Statistical relevance of PI_M is measured using the null hypothesis that $\forall i,\ c_i = m$, which corresponds to the complete spatial randomness. Under this hypothesis the population value of $PI_M$is 0. However, we have shown in (Warrell et al., 2016) that the empirical distribution of PI_Mfollows the binomial distribution asymptotically. Thus, the binomial test was used to evaluate the statistical relevance of $PI_M$for a given set of images.

mRNA / protein distribution profile

In order to define a spatial distribution profile of mRNAs and proteins for images acquired at a given time point, we used the fine-grained quantization of the cells (see paragraphs IV.4.B and IV.4.C). A single vector was computed at each time point by averaging across the pool of acquired images, hence estimating its expected value at that time point. Recall, that for each cell we computed a vector $C = (c_i)$ of per segment signal (mRNA or protein) concentration statistics and that these vectors are comparable. Then for a given time point we computed a median spatial profile $\bar{C} = (\bar{c}_i)$ representative of this time point by averaging per segment all C_i for this acquisition series.

Colocalization score

The goal of this analysis is to measure the interdependence between the mRNA and protein spatial distributions. To do this, we defined the *Colocalization Score* (CS) as an effect size computed based on correlations between mRNA and protein spatial distributions for image acquisitions that we applied for several sets of time points: those where protein acquisition time is greater that mRNA acquisition time and the others. Colocalization Score for a given mRNA-protein pair is calculated based on mRNA and protein distribution descriptor vectors $\bar{C}_{mRNA}$ and $\bar{C}_P$ and takes values between 0 and 1.

Notice that CS can be calculated for any measure of correlation between mRNA and protein distributions, which allowed us to examine the interdependence of a molecule's dynamics within specifically defined subcellular regions and at different time points.

More formally, we supposed a 2-measure discrete time process Φ, containing observations (ϕ_{t_i}) and (ψ_{t_j})at time points $T_1 = \{t_i\}$ and $T_2 = \{t_j\}$, respectively. Then, the (empirical) Colocalization Score is defined for pairs of data $S = \{(\phi t_i) \times (\psi_{t_j})\}$at time points T_1 and T_2 using a similarity function γ and the Vargha and Delaney's A12 statistics [Vargha and Delaney, 2000], a measure of stochastic superiority when comparing two distributions (generalized effect size):

$$A_{12} = P(\alpha > \beta) + 0.5P(\alpha = \beta)$$

where α and β are the rank-sums $\alpha = \sum_{t_1,\, t_2} r_{\gamma'}\left((\phi_{t_1}, \psi_{t_2})\right),\ t_1 < t_2$and $\beta = \sum_{t_1,\, t_2} r_{\gamma'}\left((\phi_{t_1}, \psi_{t_2})\right),\ t_1 > t_2$, respectively and where r_γ is the rank of the tuple where the order is given by the γ function. This γ function is the similarity between pairs of observations is computed as $\gamma'\left((t_1,t_2)\right) \rightarrow \gamma(\widehat{E}[\sigma(\phi_{t_1})],\widehat{E}[\sigma(\psi_{t_2})])$. The 'forward-leading' set S_1 is defined as $S_1 = \{(t_1,t_2) \in T_1 \times T_2 \mid t_1 < t_2\}$, and its complement S_2 contains all pairs of time-points such that $t_1 \geq t_2$. Thus, A_{12} can be understood as the effect size between the rank-sum of the similarities $\gamma'(t_1,t_2)$ across all 'forward-leading' time point pairs versus the rank-sum of the similarities $\gamma'(t_1,t_2)$ across all the other time-points. Notice that A_{12} statistics lies in the $[0,1]$ interval.

In practice, for the analysis of mRNA / protein colocalizations, we considered computing the CS for a 2-measure discrete time processes Φ in which ϕ is a point process and ψ a general random measure (representing mRNA locations and protein concentrations

respectively). In the current study we used relative density distributions of mRNA at $T_1 = \{2, 3, 4, 5\}$ and proteins at $T_2 = \{2, 3, 5, 7\}$(the discrete time points representing time in hours), and γ is the Pearson Correlation Coefficient between two relative density distribution vectors (see "mRNA/protein distribution profile"). We note that these distributions can be computed based on a particular quantization of cells and can cover the whole cell (forming a global CS) or a some section of the cell (e.g. the periphery).

Notice that measuring a correlation between a local density of some mRNA in a given voxel and its protein counterpart at a later time point is dependent on the choice of timepoints and how dynamic the molecules are. To alleviate this issue, instead of considering isolated voxels, we consider their direct neighborhoods.

Moreover, from the rank-sum of the similarities $\gamma'(t_1, t_2)$ across S_1 defined previously, an exact permutation test can be derived to calculate significance levels for a given value of A_{12} and a steady-state null hypothesis.

Degree of clustering (Ripley-K)

The degree of clustering statistic has been previously introduced based on the framework of point processes by (Lee et al., 2013). It is a unitless measure that can be used to compare clustering between different molecules and conditions. In (Warrell et al., 2016) we generalized this definition to the framework of continuous random measures, which allows us to calculate the degree of clustering for both FISH and IF data, the former being modelled as point processes, and the latter modelled as a continuous-valued random measure. Our generalized algorithm for calculating the degree of clustering is summarized below. For theoretical considerations please see (Warrell et al., 2016).

A classical tool for the point process analysis is the Ripley's K function defined as the mean number of events that occurred inside a ball of radius r around a randomly selected event normalized by λ, the number of events per unit area (Ripley, B. D. 1977). A classical estimator of the Ripley's K function can be defined as in (Chiu et al., 2013; Ripley 1977):

$$\widehat{K}(r) = \frac{1}{\lambda^2 \nu(w)} \sum_{i=1}^{n} N_i(r)$$

where N_i is the number of event points (mRNA transcripts) in a ball of radius r centered on i, λ is the density, ν is the volume or area (in 3D and 2D, respectively) of the observed region w, and n is the number of points.

We normalized $\widehat{K}$ under a homogeneous Poisson process, which is commonly known as Ripley's H function $\widehat{H}(r) = \sqrt[d]{(3\,\widehat{K}(r))/(4\pi)} - r,\ d \in \{2, 3\}$ where d is equal to 3 in the case of volume-based computation and 2 in the case of 2D.

This in turn makes it possible to define the clustering index H^* as an estimator of $\widehat{H}(r)$ by comparing the Ripley's H function calculated empirically to its distribution under complete spatial randomness (CSR):

$$\widehat{H^*}(r) = \begin{matrix} \widehat{H}(r)/\widehat{H}_{95}(r) \ \ if \ \ \widehat{H}(r) \geq 0 \\ -\widehat{H}(r)/\widehat{H}_5(r) \ \ otherwise \end{matrix}$$

where $\widehat{H}_{95}(r)$ and $\widehat{H}_5(r)$ are the 95th and 5th percentiles respectively of $\widehat{H}(r)$.

CSR is modeled using random permutations of actual data points (100 times in our study), which enabled us to compute the 95% and 5% confidence bounds of CSR. Spatial clustering is considered to be significant at radius r if the computed $\widehat{K}(r)$ is over the upper (95%) or lower (5%) bounds of the random distribution.

In (Warrell et al., 2016) we have introduced a convolution-based $\widehat{H^*}$ estimator based on the random permutation-test. This estimator normalizes $\widehat{H}(r)$ so that $|\widehat{H^*}(r)|$>1 only when $\widehat{H}(r)$ falls outside the 95% confidence interval for a homogeneous Poisson Process. Moreover, we have shown that our permutation test using the convolution-based estimator reduced to the clustering index estimator used by (Lee et al., 2013) for the point process case. This enabled the implementation of a common consistent computational framework for both point and continuous processes. The degree of clustering $\widehat{\delta}(r)$ is then defined as the area of $\widehat{H}^*$ above 1, that is $\widehat{\delta}(r) = \int_{x \in (0,r)} max(\widehat{H}(x) - 1, 0)dx$.

Within this unified framework we can use the same computational approach for mRNA data as in (Lee et al., 2013) to compute the degree of clustering. Below we define the specific procedure for its computation in the case of protein data.

Cells are quantized into voxels $V_1, \ldots, V_n$ where each voxel t the value of the observed quantity $\varphi(V_1), \ldots, \varphi(V_n)$ in the case of 3D analysis (or into pixels in the case of 2D). We denoted by I an array in which each element corresponded to the intensity value for each voxel.

The convolution-based estimator $\widehat{K_c}$ can be computed using the following formula: $\widehat{K_c}(r) = \frac{1}{\lambda^2 V} \int [|x| \leq r](I * I')(x)dx - \frac{1}{\lambda}$ where λ is an estimate of average intensity per unit volume, [.] is the indicator function that is 1 for a true statement and 0 otherwise, $I'(x) = I(-x)$, $*$ is the convolution operator, and V is the volume of the window over which the cell is observed. In practice, given the fact that the cell thickness is quite low, we can approximate the 3D convolution by a 2D convolution.

Thus we have a common computational framework to evaluate the presence or absence of clustering for both mRNA and protein data.

Additional methods for muscle data analysis

In this section we report adaptations of the methods presented in "primary image descriptors," "secondary image descriptors," and "additional methods for muscle data analysis" to the case of muscle cells (see the table describing the Image acquisition characteristics for muscle cells).

Cell and nucleus masks, nucleus centroid

Cell and nucleus masks were computed for all muscle images, using γ-Tubulin and DAPI signals, respectively, using the same general principles as in "peripheral distance map." As these acquisition series benefit from a better segmentation, only Otsu threshold method was necessary to obtain the binarized images.

After these steps, we obtained a contiguous contour with small white spots due to noise in the images. We applied mathematical morphological operators such as dilatation and closing to get a full mask of the muscle cell $M^{cell}(x,y)$. The nucleus mask $M^{nucleus}(x,y)$ detection followed the exact same steps and parameters as for the micropatterned images - the nucleus centroid was computed as the geometric center of the nucleus mask.

mRNA signal detection

mRNA spots detection was done using ICY spot detector (Olivo-Marin 2002) to find transcript positions, the parameters were set to: 1 pixel and 2 pixel length-scales with a fixed sensitivity of 80.

Z-lines' masks

The main component of Z-lines is the Alpha actinin protein. To facilitate the analysis, we have defined an additional secondary descriptor computed from the Phalloidin signal, called Z-lines mask $M^{Z-lines}(x,y)$.

For each z-slice of each image we performed the contour detection for the Z-lines. First, we applied a vertical Sobel operator, which detected the vertical edges of an image, followed by a Gaussian kernel to smooth artifacts of the Sobel filtering and reinforce the Z-line signal. An Otsu binarization was then processed. As a result we obtained a set of Z-lines masks $M^{Z-lines} = \{M_z^{Z-lines}(x,y)\}$ (Figure 7).

For further analysis we restricted the cell to z-slices containing more than 25 mRNA spots (to avoid false positives due to high noise). Notice that the spots falling in the eliminated slices were also excluded from the analysis.

We defined an additional descriptor called Z-line spacing Z that represented the median spacing between two lines. For each z-slice at each y coordinate we computed all the distances $d((x_i,y),\ (x_j,y))$ where x_i and x_j were 2 consecutive Z-lines contours. $Z$was defined as the median of all d for all acquired cells. For our data $Z = 15$ pixels.

Z-line mRNA distance profile

In order to evaluate the mRNA clustering in the vicinity of Z-lines, we computed the average 2D Euclidean distance of mRNA spots to their nearest Z-line for mature and immature cells.

Using the $M^{Z-lines}(x,y)$masks, we computed for each mRNA spot $m \in M$ positioned at (x,y,z_m) the minimal 2D Euclidean distance to a Z-line falling within a disk of radius Z. This computation was performed within the $M_z^{Z-lines}(x,y)$ z-mask such that $z = z_m$. If m fall within $M_z^{Z-lines}(x,y)$, then the minimal distance was set to 0. Thus for each image we obtained a $D = \{d\},\ |D| = N$ the set of all minimal distances between mRNA and Z-lines. In turn this allowed us to define for each image a count vector $\delta = (\delta_i)$ of size Z where δ_i is the number of mRNA spots at each distance $d = i,\ d \le Z$ normalized by N.

For a given image acquisition we defined its Z-line mRNA distance profile to be $\bar{\delta} = (\bar{\delta}_i)$, where $\bar{\delta}_i$ is the median of all δ_i (Figure 7B).

Cell quantization

Given that muscle cells contained more than one nucleus, each cell mask was restricted to be between two consecutive nuclei centroids as shown on the schematic in Figure 7C). Given a cell mask $M^{cell}(x,y)$, definition of cell mask tessellation in n vertical segments $q_i \ldots q_n$ enables cells quantization in 2D in terms of per segment statistics of mRNA concentrations. Quantization was performed with $n = 20$ and $n = 80$, see results in Figure 8C.

mRNA spatial distribution

To estimate mRNA clustering along muscle cells, we computed local mRNA density for each cell using the cell quantization introduced in "cell quantization." We denote by t_i the total number of mRNA spots falling in a given q_i. Then the local mRNA density is computed in the same way as for fibroblast cells (see "peripheral fraction and enrichment") as the relative concentration c_i of mRNA in q_i and is defined to be $c_i = \frac{t_i/a_i}{T_{mRNA}/A}$, where A is the cell mask area. We produced distribution plots and heatmaps representing mRNA local density between two nuclei (Figure 7C).

Additional methods for CHX inhibition analysis

Image processing has been performed for Arhgdia (Supp. control), Arhgdia (CHX), Pard3 (Supp. Control), Pard3 (CHX) (see table describing image acquisition characteristics for CHX) as presented above for primary and secondary image descriptors except for the FISH signal being used to detect cell masks for mRNA images. The number of acquired mRNA images was quite low for these experiments, consequently we have only performed global analysis (compared to the datasets where we could analyse time point by time point). We performed peripheral fraction enrichment and degree of clustering analyses in the same way as described in "peripheral fraction and enrichment" and "degree of clustering (Ripley-K),", respectively, except for 2 points: (i) all the analysis was performed in 2D, (ii) for mRNA the computation was done using the continuous signal in the same way as for the IF, similarly to Stueland et al., 2019.

DypFISH software code (in Python) as well as data (in HDF5 format) used in this study are freely available from http://www.dypfish.org and https://github.com/cbib/Dypfish.

Cell Reports Methods, Volume *1*

Supplemental information

Interrogating RNA and protein spatial subcellular distribution in smFISH data with DypFISH

Anca F. Savulescu, Robyn Brackin, Emmanuel Bouilhol, Benjamin Dartigues, Jonathan H. Warrell, Mafalda R. Pimentel, Nicolas Beaume, Isabela C. Fortunato, Stephane Dallongeville, Mikaël Boulle, Hayssam Soueidan, Fabrice Agou, Jan Schmoranzer, Jean-Christophe Olivo-Marin, Claudio A. Franco, Edgar R. Gomes, Macha Nikolski, and Musa M. Mhlanga

SUPPLEMENTARY FIGURES

Figure S1, related to Figure 2

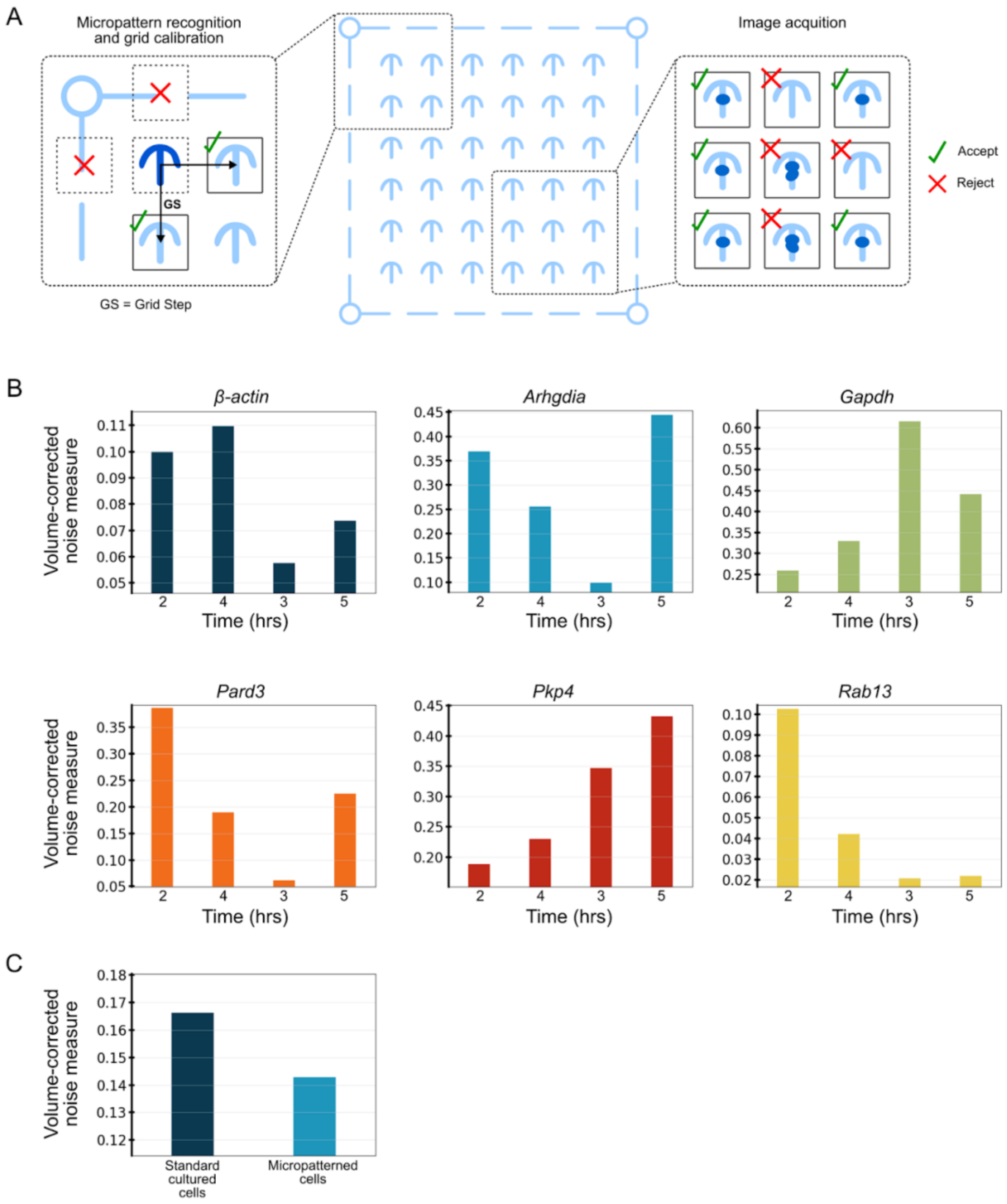

Figure S1, related to Figure 2 | Automated image acquisition and effects of micropatterning on noise. (A) Each coverslip was micro-fabricated to contain multiple 12 by 12 grids of micropatterns to which the cells adhered, facilitating the development of an algorithm to automate the process of image acquisition. The algorithm initially performed grid calibration in which the location of the upper-left micropattern was automatically detected, followed by grid orientation and grid step size determination (left). Images were then collected at each grid position across the full 12 by 12 grid. A 2-class support vector machine was trained to classify cells which have grown normally on the micropatterns versus micropatterns containing no cells, multiple cells, or cells which have failed to fill the micropattern, allowing the automatic rejection of grid positions which cannot be used (right). (B) Mouse fibroblasts were plated on fibronectin-coated micropatterns and induced to polarize by addition of serum. Cells were fixed and single molecule FISH was performed to target mRNAs of interest. The volume-corrected noise measure (Padovan-Merhar et al., 2015) was compared

across time for 6 mRNAs at 2, 3, 4 and 5h time points. (C) The stochasticity, which remains after correcting for the linear relationship between cell-size and transcript number using the volume-corrected noise measure (Padovan-Merhar et. al. 2015).

Figure S2, related to Figure 3

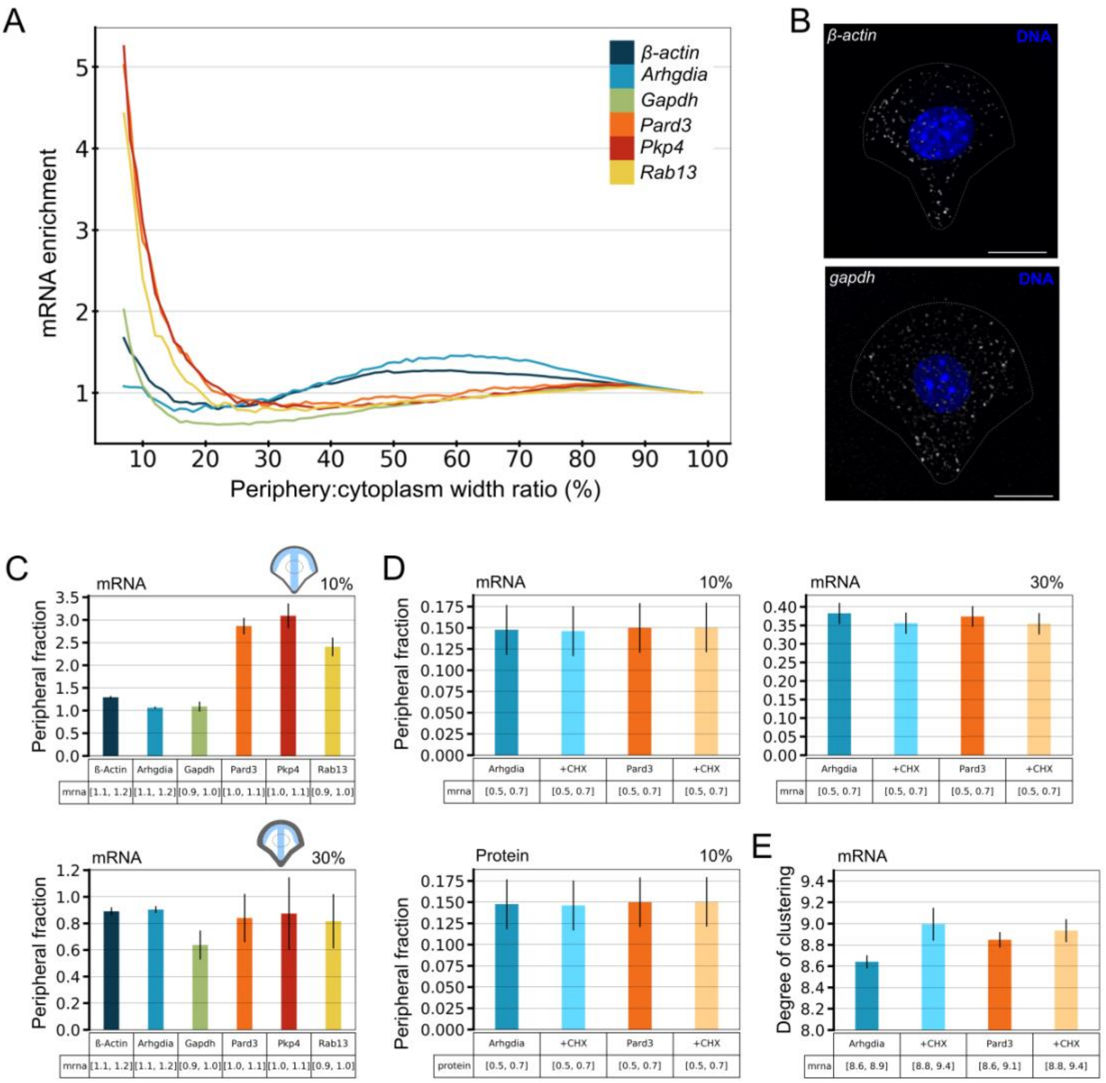

Figure S2, related to Figure 3 | Peripheral enrichment and clustering dynamics of mRNA-protein pairs. (A) Comparison of the enrichment of 5 mRNAs in a peripheral cellular region whose width varies from 0-100% of the radial distance from the plasma membrane to the nucleus. (B) Representative smFISH images for *β-Actin* and *Gapdh* transcripts. (C) Distributions of fractional values for peripheral enrichment of all mRNA transcripts at 10% and 30% of the radial distance from the plasma membrane to the nucleus. (D) Distributions of absolute fractional values for peripheral enrichment of Arhgdia and Pard3 mRNA and proteins in cells treated with CHX compared to control cells at 10% and 30% (only for mRNAs, corresponding graph for proteins in main Figure) of the radial distance from the plasma membrane to the nucleus. (E) Degree of clustering for *Arhgdia* and *Pard3* in cells treated with CHX compared to control cells. Differences in degree of clustering were assessed using a Mann-Whitney test p-value (*Arhgdia* vs *Arhgdia*+CHX = 0.092; Pard3 vs Pard3+CHX = 0.71). Error bars show the standard error of the median.

Figure S3, related to Figure 4

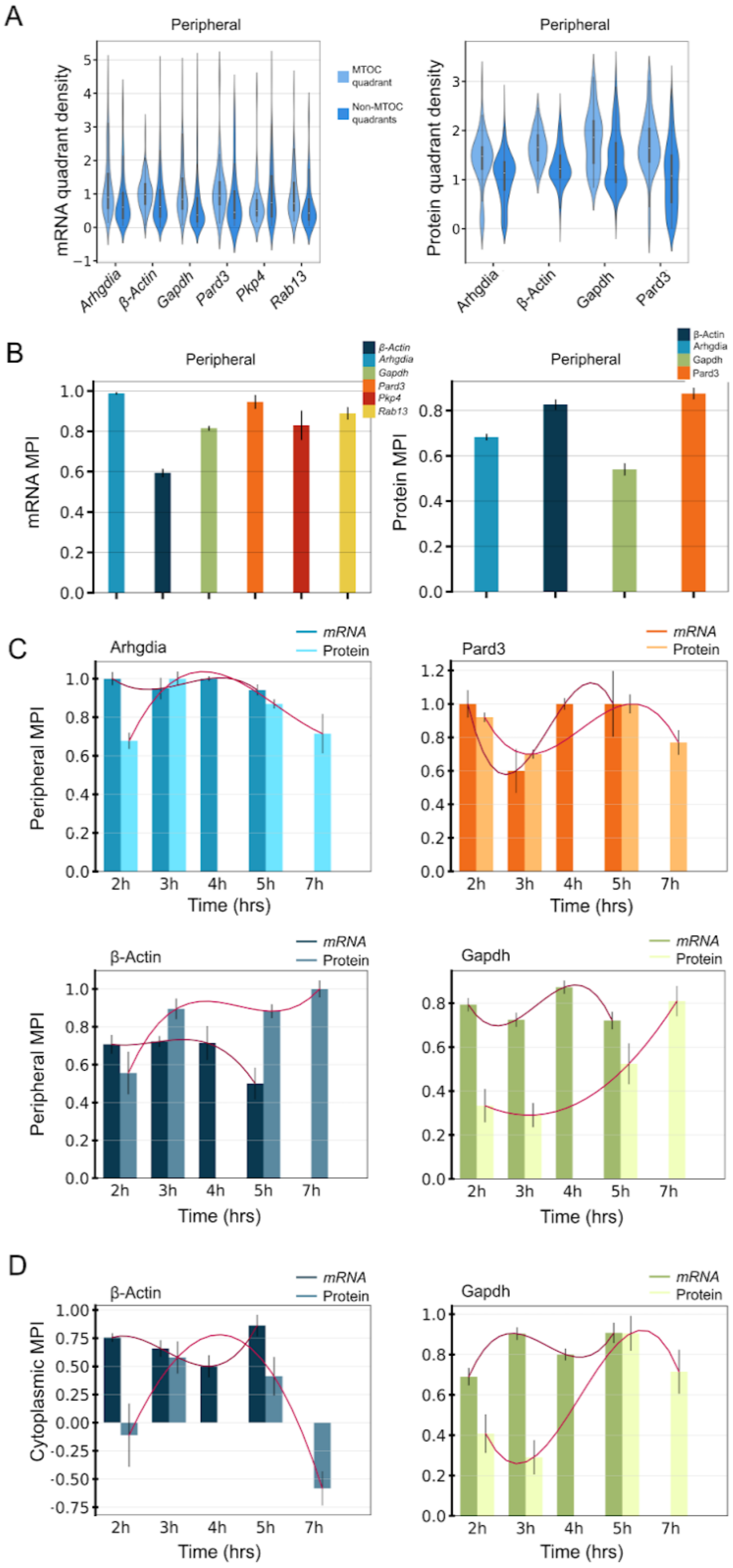

Figure S3, related to Figure 4 | Relationship between peripheral mRNA and protein distributions and MTOC position. (A) The cytoplasmic mRNA and protein relative density in non-MTOC containing quadrants (dark blue) and MTOC-containing quadrants (light blue), enrichment in the MTOC-containing quadrant is defined by differences of means of these distributions. (B) Comparison of MPI values for mRNAs and proteins in peripheral populations (all time points). (C) Comparison of peripheral MPI dynamics for β-Actin, Gapdh

Arhgdia and Pard3 mRNA-protein pairs. (D) Comparison of cytoplasmic MPI dynamics for Gapdh and β-Actin mRNA-protein pairs. Bar graphs in (B) show median and standard deviation from the median error bars for 100 bootstrapped MPI estimates. Graphs in (C) and (D) show median surrounded by envelope indicating standard deviation from the median error bars of 100 bootstrapped estimates fitted to cubic splines.

Figure S4, related to Figure 4

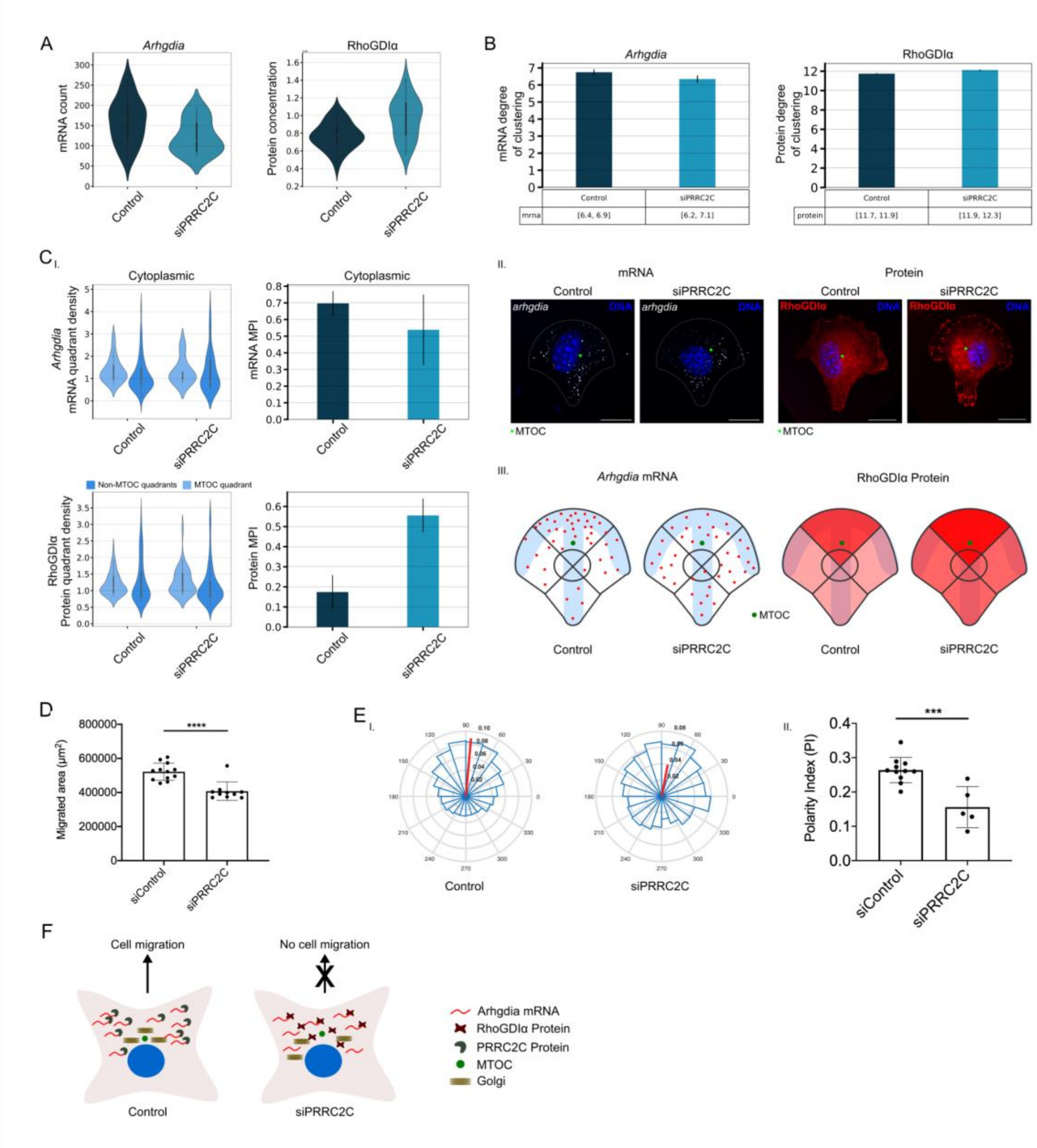

Figure S4, related to Figure 4 | Depletion of PRRC2C, an *Arhgdia* binding protein, decreases *Arhgdia*'s MTOC polarity and causes defects in cell migration. (A) Total *Arhgdia* mRNA count and RhoGDIα (Arhgdia protein) concentration in control and PRRC2C siRNA depleted 3T3 fibroblasts at 3 h post induction of polarization. (B) Comparison of degree of clustering for *Arhgdia* mRNA and protein (RhoGDIα) at 3 h (log values shown after scaling by natural log for mRNAs and proteins). Error bars show the standard error of the mean. (C) The cytoplasmic *Arhgdia* mRNA and protein (RhoGDIα) quadrant density and MPI values in control and PRRC2C-depleted cells (i). Representative images are shown for *Arhgdia* smFISH and RhoGDIα

immunofluorescence, as well as DAPI stain, scale bar 10 μM (ii). The cartoon in (iii) represents the cytoplasmic MPI values in control and PRRC2C-depleted cells for *Arhgdia* and RhoGDIα. (D) Quantification of migrated area from siControl and siPRRC2C transfected HUVECs after 16 hours of migration (n=1; mean ± SD of 3 replicates/condition). (E) Rose plots showing the polarization angles distribution of 1st to 4th rows from siControl (n=2; mean ± SD of 11 replicates) and siPRRC2C (n=2; mean ± SD of 5 replicates) transfected HUVECs. Bar plots with individual data points representing the polarity index of each replicate from siControl (n=2; mean ± SD of 11 replicates) and siPRRC2C (n=2; mean ± SD of 5 replicates) transfected HUVECS. (F) Model describing the effect of PRRC2C depletion on *Arghdia* MTOC polarity, subcellular localization and degree of clustering, RhoGDIα translation and subsequent cell migration.

Figure S5, related to Figure 4

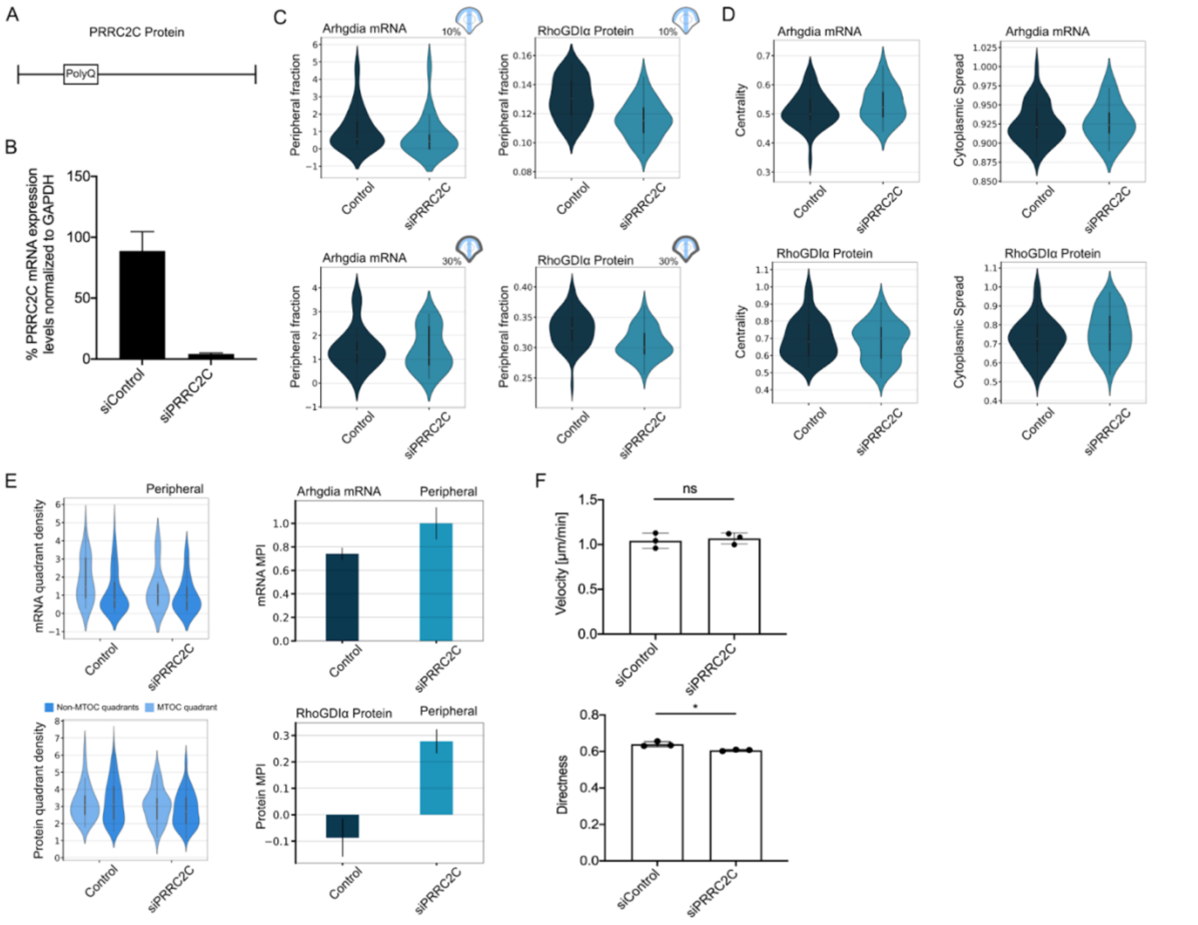

Figure S5, related to Figure 4 | Depletion of PRRC2C, an *Arhgdia* binding protein, decreases *Arhgdia*'s MTOC polarity and causes defects in cell migration. (A) The PRRC2C protein contains a PolyQ motif. (B) Quantification of PRRC2C mRNA levels normalized to GAPDH from siControl (n=1; mean ± SD of 2 replicates) and siPRRC2C (n=1; mean ± SD of 3 replicates) transfected HUVECs. (C) Distributions of fractional values for peripheral enrichment of *Arhgdia* mRNA and protein (RhoGDI) at 3 h post induction of polarization, at 10% and 30% of the radial distance from the plasma membrane to the nucleus. (D) Centrality and cytoplasmic spread of *Arhgdia* transcript and protein (RhoGDI) at 3 h post induction of polarization. The centrality is defined as a statistics measuring the evenness of a molecule spread across the cell, with the value 1 for even distribution. (E) The peripheral *Arhgdia* mRNA and protein (RhoGDI) quadrant density and MPI values in control and PRRC2C-depleted cells. Error bars show the standard error of the mean. (F) Quantification of cell velocity from siControl and siPRRC2C transfected HUVECs during 16 hours of migration (n=1; mean± SD of 3 replicates/condition) (top) and of migration directness from siControl- and siPRRC2C-transfected HUVECs during 16 hours of migration (n=1; mean± SD of 3 replicates/condition) (bottom).

Figure S6, related to Figure 5

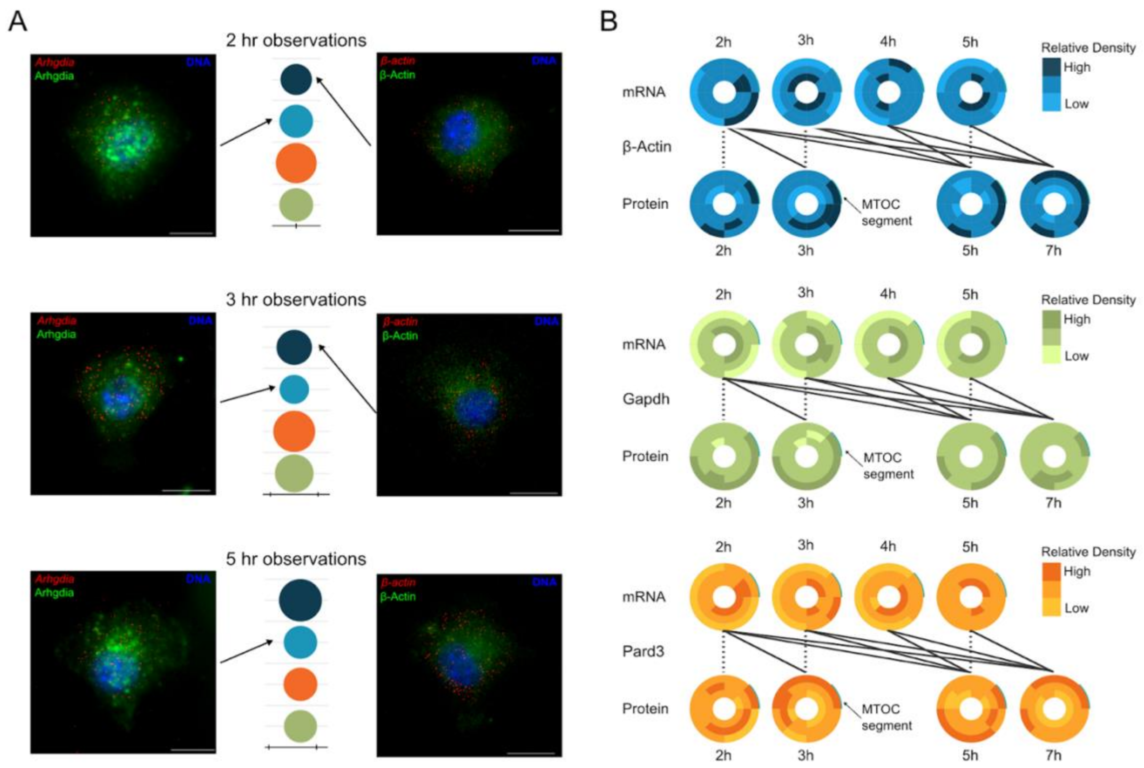

Figure S6, related to Figure 5 | Interdependency of localization dynamics for corresponding mRNAs and proteins. (A) Representative images for Arhgdia and β-Actin where smFISH and IF were performed in the same cell at 2 h, 3 h and 5 h post induction of polarization, scale bar 10 μM. (B) Forward-leading' time point pairs, defined as t1<t2 (connected by solid lines), were used for calculating Colocalization Score values. The 'forward-leading' time points were chosen as we considered the additional time for translation to occur once the mRNA is localized. Cytoplasmic density maps representing relative density vectors based on fine-grained quantization for Gapdh, Pard3 and β-Actin mRNA and protein for each time point are shown. Celular regions are dark blue if the local relative density is greater than the mean density of all segments in the cytoplasm + standard deviation, light blue if the local relative density is smaller than the mean of all segments in the cytoplasm - standard deviation, intermediate values correspond to the relative density within the [-σ, σ]interval. The MTOC-containing quadrant is highlighted by a thin turquoise stripe.

Figure S7, related to Figure 6

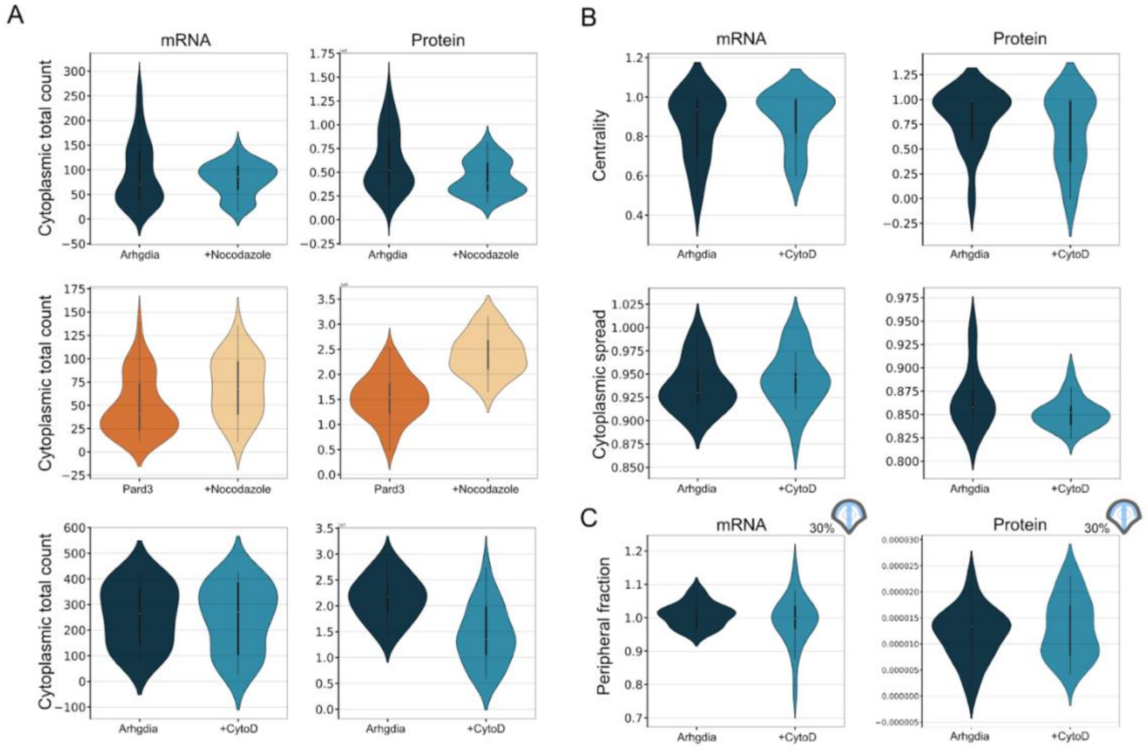

Figure S7, related to Figure 6 | Effects of cytoskeleton disturbance on mRNA-protein centrality, cytoplasmic spread, mRNA and protein counts and peripheral fraction. (A) + (B) Effects of nocodazole and CytoD treatment on cytoplasmic total count, centrality and cytoplasmic spread descriptors for Arhgdia and Pard3 mRNAs and proteins at 3-5 h time-points. Error bars show the standard error of the mean. (C) The peripheral fraction of Arhgdia transcript and protein (at 3 and 5 h combined) were calculated similarly to Figure 6C.

Figure S8, related to Figure 7

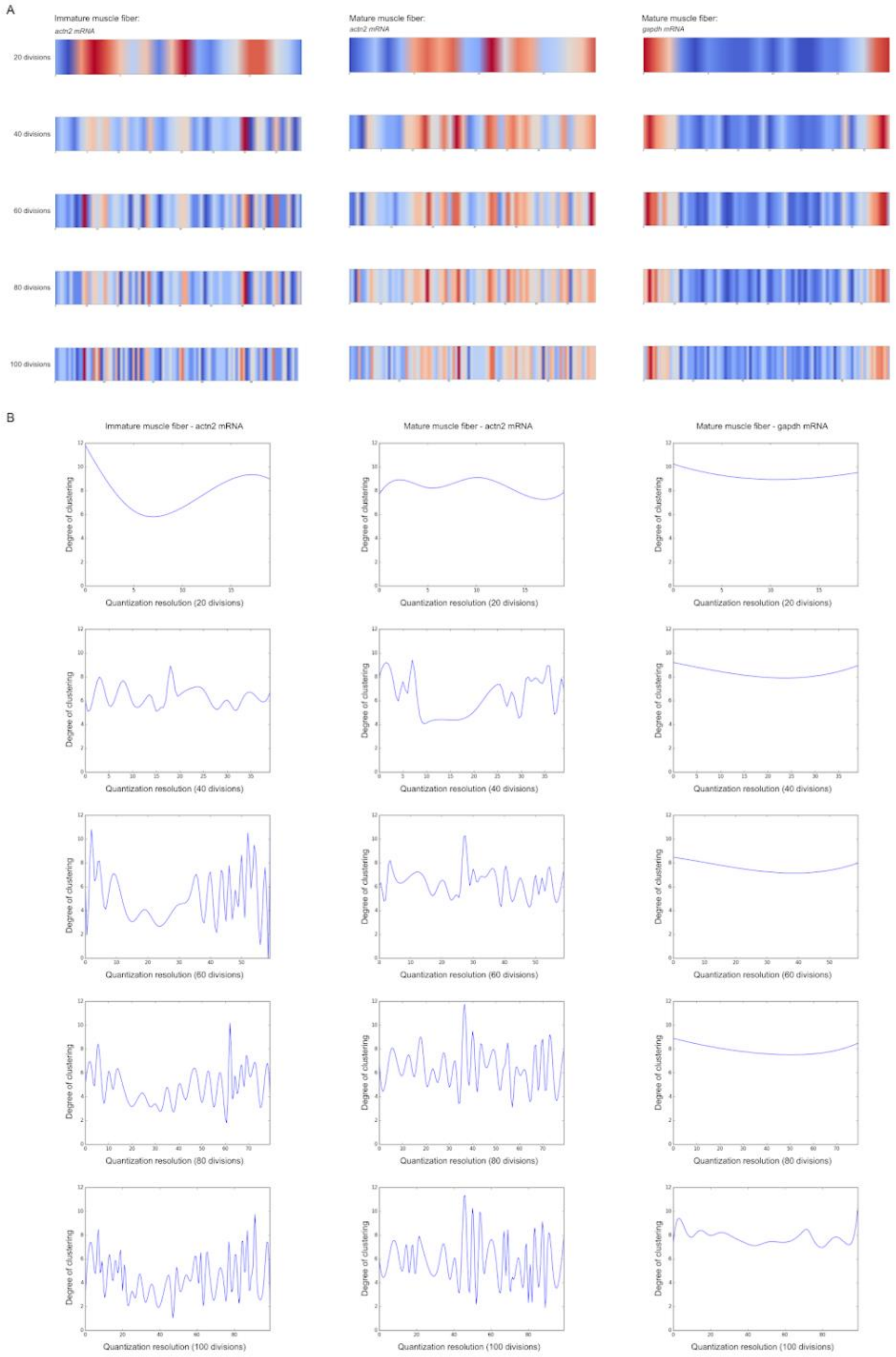

Figure S8, related to Figure 7 | Sarcomeric mRNAs cluster in a striated pattern in differentiated myofibers. (A) + (B) The mRNA local density was computed between two nuclei. Each cell was quantized in vertical quadrants and relative concentration of mRNA in each quadrant was computed by normalizing the counts by the relevant surface. A wave-like clustering is observed for *actn2* in mature compared to immature fibers. No clustering is observed for *Gapdh*.
